# Educating the masses to address a global public health priority: The Preventing Dementia Massive Open Online Course (MOOC)

**DOI:** 10.1371/journal.pone.0267205

**Published:** 2022-05-04

**Authors:** Maree Farrow, Hannah Fair, Shannon Z. Klekociuk, James C. Vickers

**Affiliations:** Wicking Dementia Research and Education Centre, College of Health and Medicine, University of Tasmania, Hobart, Tasmania, Australia; Universita degli Studi della Campania Luigi Vanvitelli, ITALY

## Abstract

Dementia is a global public health priority and risk reduction is an important pillar of the public health response. While 40% of cases are estimated to be attributable to modifiable health and lifestyle risk factors, public awareness of the evidence is low, limiting peoples’ opportunity to adopt risk-reducing behaviours. To address this gap, we designed, implemented, and evaluated an educational intervention, the Preventing Dementia Massive Open Online Course (PDMOOC). This mixed-methods study examined the reach and impact of the free and globally available PDMOOC, to assess its potential to provide effective dementia risk reduction education to a broad international audience. Over 100,000 individuals participated in the PDMOOC across seven iterations from 2016 to 2020, with 55,739 of these consenting to participate in research. Their mean age was 49 years (SD = 15), they came from 167 different countries, and the majority were female (86%), had completed post-secondary education (77%), lived in high-income countries (93%) and worked in health care and social assistance (63%). This demographic profile changed across time, with more men, people with higher education and people from low- and middle-income countries participating in recent course iterations. Two-thirds of participants completed the PDMOOC; completion was associated with being aged 50 to 70 years, residing in a high-income country, having tertiary education, and working in the health sector. Participants reported high levels of satisfaction with the PDMOOC, improved dementia risk reduction understanding and self-efficacy, increased motivation to maintain healthy lifestyles, and, importantly, application of their learning to health behaviour change with the potential to reduce their dementia risk. The PDMOOC educated a large global audience about dementia risk reduction, which contributed to participants making risk-reducing behaviour changes. This suggests MOOCs can be a successful public health strategy to improve dementia risk reduction understanding.

## Introduction

The prevalence and subsequent impact of dementia is rapidly growing and there remains no cure. Consequently, dementia is a global public health priority, and attention is increasingly turning to preventive health strategies to ameliorate increasing prevalence [1, 2]. Recent estimates indicate that 40% of dementia cases globally are attributable to twelve modifiable health and lifestyle risk factors [1], with even higher estimates found by similar analyses for Australia (48% of cases attributable to seven risk factors [3]) and Latin America (56% of cases attributable to nine risk factors [4]). Positively, several studies have demonstrated a decline in the age-specific incidence of dementia in Europe and North America over the past ten to twenty years (for review see [5]). Improved access to early life education and management of cardiovascular risk factors such as hypertension are believed to have contributed to prevention of cases of dementia and/or delayed onset [6]. This provides evidence for the potential impact of population level dementia prevention strategies targeting modifiable risk factors across the life-course [2, 7].

Despite major scientific advancements in understanding dementia risk and prevention, public understanding of the evidence remains poor [8, 9]. A recent global survey revealed that two-thirds of respondents thought dementia was a normal part of ageing, and one-quarter believed nothing could be done to prevent it [10]. Media reports frequently highlight prevention strategies for which there is little or no evidence, with misinformation compounded by the broader public’s limited level of understanding about risk and evidence. As everyone has some risk of developing dementia, there is a need for interventions that are accessible to as many people as possible, and that improve dementia risk knowledge and self-efficacy for risk reduction. Whilst there are more dementia risk reduction initiatives emerging, many reach a limited proportion of the population, often in higher income countries, because their delivery is face-to-face and/or within a research context [11–13]. A vital step in the public health response to dementia is to increase knowledge in the community about the risk factors and the potential for prevention [9, 14]. One of the ways in which knowledge of dementia risk reduction may be increased is through online educational interventions.

Massive Open Online Courses (MOOCs) are a way of providing access to education to a general audience on an unprecedented scale leveraged by the internet [15]. MOOCs are an attractive mode of delivery because they are potentially unlimited with regards to their reach; they are typically free, and open to anyone in the world with an internet connected device and the requisite computer and language skills [16]. Further, the pedagogical structure of MOOCs makes them ideal for increasing knowledge around health, reflected in the increasing number of health and medicine based MOOCs available (https://www.classcentral.com/subject/health), which have completion rates that exceed the global average for MOOCs [17]. Health-related MOOCs can provide education to the general public on specialist topics helping to improve health literacy and self-management of health and wellbeing [18]. MOOCs may be a particularly effective mode to deliver dementia risk reduction education because of the global applicability of the topic and the need to address risk reduction across the life-course.

While MOOCs are being increasingly used to provide health information to the public, there is a paucity of research evaluating the efficacy of these programs [17]. The characteristics of people accessing MOOCs needs to be examined to determine if these courses are indeed reaching target audiences and people from a wide range of backgrounds. People’s ability to complete MOOCs and their subjective experiences of the courses also need to be examined to determine if MOOCs are promoting continued participation and providing accessible, engaging, and useful content. Finally, the outcomes of participating in MOOCs need to be examined to determine the effectiveness of this approach in increasing knowledge and promoting health behaviour change. The current study is a mixed methods examination of the Preventing Dementia (PD) MOOC using participant enrolment and feedback surveys. To evaluate the impact of the PDMOOC against its objective of improving dementia risk reduction awareness, we describe the characteristics of people who undertake the PDMOOC, their course progression and completion, their subjective outcomes from their learning, and the potential impact of that learning on their health and lifestyles.

## Methods

### Participants

This study was approved by the Tasmania Social Sciences Human Research Ethics Committee (Reference number H0015773).

The PDMOOC was actively marketed via a range of strategies including radio and print media interviews, paid print, online and social media advertising, and presentations and presence at community events. The MOOC was open to anyone, anywhere in the world, with an interest in participating and the means to do so (internet access). Interested people could sign up for the course using the customised online learning platform. On enrolment (2016–2017) or on commencing the course (2018–2020), participants were presented with an online information sheet explaining the purpose and requirements of the research and asked to provide research consent by completing an online form. They were able to digitally select a radio button indicating either ‘yes’ they consented to participating in the research or ‘no’ they did not consent, and their choice was recorded in the learning platform database. The research was open to anyone enrolled in the PDMOOC aged 18 years and older who consented to their data being used for research purposes.

### The MOOC

The PDMOOC is a short online course that presents the latest evidence about dementia risk factors and aims to build knowledge and enable management of modifiable dementia risk as well as an ability to evaluate evidence related to such risk (https://www.utas.edu.au/wicking/preventing-dementia). The PDMOOC is comprised of modules released on a weekly basis, with each module consisting of approximately 2 hours of content and activities, plus brief introduction and completion sections. Course material includes pages of text and images, videos, discussion boards and quizzes. Rich learning materials such as explanatory animations are embedded into the course, as well as feedback from content experts on participant discussion posts. The course is free, can be undertaken flexibly on a personal computer, tablet, or mobile phone, and is scalable to very large numbers of participants.

Seven PDMOOC iterations run between July 2016 and May 2020 were included in this study. The course content remained relatively consistent across these iterations, with amendments focusing on the latest evidence. In 2016 and 2017 the course was presented in five modules (1. Can dementia be prevented?; 2. Risk factors for dementia; 3. Dementia risk–it’s not all in your head; 4. A healthy and active mind; 5. Interventions for prevention) released across five weeks, with quizzes at the end of modules two, four and five. A 70% or greater quiz grade was required to progress to the next module. In 2018 the course duration was condensed to allow two offerings per annum, and from 2018 to 2020 there were four modules (1. Can dementia be prevented?; 2. Dementia risk–it’s not all in your head; 3. A healthy and active mind; 4. Interventions for prevention) released across four weeks with a quiz at the end of each module. All iterations provided information about the potential for dementia prevention at the population level and risk reduction at the individual level, summaries of evidence for non-modifiable and modifiable risk factors presented by experts in the respective fields with links to further reading, myth busting of common misconceptions of risk factors for which there is no or inconsistent evidence, information about behaviour change barriers and enablers, and information about current dementia prevention research initiatives. Participants had opportunities to contribute to discussion boards on specific topics, however due to the large numbers of participants they had limited opportunity to have specific questions about their own risk answered by the PDMOOC team.

### Measures

Participants were invited to compete enrolment and background surveys prior to commencing the PDMOOC and a feedback survey after completing the course. Survey responses from consenting participants were collated across the seven PDMOOC iterations. Unless otherwise specified, all survey questions included in this study were asked of participants across all iterations of the MOOC, though the wording of some questions varied slightly across iterations. Each participant’s most complete response to each survey was included in this dataset. Only one enrolment from each consenting participant was included in each analysis. For individuals who enrolled in multiple iterations, enrolment and background surveys, and progression in and completion of the PDMOOC, from their first enrolment were included in this dataset. For the course feedback survey, the survey from each participant’s first completion of the course was included.

Enrolment and background surveys collected demographic data including age, gender, highest level of education, occupation and employment organisation type, and country of residence. Participants who specified ages of less than 18 were excluded from analyses, in accordance with the ethics approval for this project. Age values of greater than 100 were deemed to be user errors and considered missing values. Participants’ specified highest levels of education were converted into the binary categories “post-secondary” (university or vocational qualifications) and “secondary or lower”. Participants’ employment organisation (if specified) or occupation (if no organisation was specified) were converted into the binary categories “health care and social assistance” and “other occupation” based on the ANZSIC Division Q occupation categories [19]. Participants who specified a gender of “other” (n = 63) and participants who stated they would prefer not to specify a gender (n = 65, option introduced in 2019) were grouped together in the category of “no binary gender specified”. This group was omitted from statistical analysis due to the comparatively small group size. The income classification of participants’ countries of residence were determined from World Bank data [20]. Country income classifications were categorised as “high-income” or “low- and middle-income”.

Questions relating to participants’ reasons for interest in the PDMOOC were asked during enrolment in 2016, not asked in 2017, and asked in the background survey in 2018–2020. In 2016 these questions were asked in tick-the-box format, where participants were asked to select all relevant statements. From 2018 onwards these questions were asked on a 5-point Likert scale. Participants who selected “agree” or “strongly agree” in iterations from 2018 onwards were considered to have answered in the affirmative, as were participants who selected statements in 2016.

Questions relating to participant’s family history of dementia were asked in all iterations other than May 2020. In 2016–2017, participants were asked about immediate relatives, with the examples of parents, grandparents, siblings and spouses, with the response options “yes” or “no”. In 2018–2019 this question was split into two parts; one asking about immediate relatives with the specification of parents, siblings, or children only, and one asking about other family members with the examples of spouse, grandparents, mother-in-law, uncle. Response options were “yes”, “no” and “unsure”. A response of “yes” to either question in 2018 and 2019 was considered equivalent to a response of “yes” to the question in 2016 and 2017.

Participants who completed all modules and quizzes of the PDMOOC were invited to complete a feedback survey. This asked about satisfaction with the course, the usefulness of the course and whether they had applied any new knowledge, and suggestions for improving the MOOC. The feedback survey consisted of free-text questions and ratings of agreement with statements using a 5-point Likert scale, with response options strongly agree, agree, neutral, disagree, and strongly disagree. For modelling the association between participant demographics and feedback survey responses, Likert scale items were condensed into two binary categories, affirmed (“agree” or “strongly agree”) and not affirmed (“neutral”, “disagree” or “strongly disagree”), due to small sample sizes for the not affirmed options.

Participants’ progression through the PDMOOC was determined by their completion of the end of module quizzes. Quiz 1 from all iterations was labelled quiz A. Quiz 2 from 2016–2017 and quiz 3 from 2018–2020 were labelled quiz B. Quiz 3 from 2016 and 2017 and quiz 4 from 2018–2020 were labelled quiz C. This equated the content delivered and assessed at these three stages of the respective MOOC iterations. Completion of the PDMOOC was determined by accessing the completion section after achieving a score of 70% or more on quiz C.

### Statistical methods

This analysis used a between-subjects design. Models for enrolment information and completion of the PDMOOC were created using data from each participant’s first MOOC enrolment. Categorical participant demographics in different MOOC iterations were modelled using a series of unadjusted binomial generalised linear models (GLMs)–one model for each demographic variable (gender, occupation, education level, and country of residence wealth). Each analysis was then adjusted for potential confounders (all other demographic characteristics, including the continuous characteristic of age) using binomial generalised additive models (GAMs). Participant age across different MOOC iterations was modelled at the 25th, 50th and 75th quantile using quantile regressions. This analysis was similarly run with and without adjusting for categorical participant demographics.

The associations between MOOC completion and participant characteristics (demographics) and MOOC iteration of enrolment were modelled using unadjusted binomial GLMs for categorical predictor variables and an unadjusted binomial GAM for the continuous predictor variable of age. An adjusted binomial GAM was then fitted to determine which participant characteristics remained significantly associated with completion after adjusting for all other characteristics.

A series of unadjusted binomial GLMs were used to determine if participants’ family experience of dementia and reasons for undertaking the PDMOOC were associated with MOOC completion. Each analysis was then adjusted for participant demographics using a series of adjusted binomial GAMs.

The associations between MOOC experiences/outcomes and participant characteristics (demographics) were modelled using unadjusted binomial GLMs for categorical predictor variables and an unadjusted binomial GAM for the continuous predictor variable of age. An adjusted binomial GAM was then fitted to determine which participant characteristics remained significantly associated with MOOC experiences/outcomes after adjusting for all other characteristics.

GAMs, binomial GLMs and quantile regression models were used to model this data as residuals were not normally distributed, and these robust models do not require normally distributed residuals. Only one entry from each participant was included in each model, ensuring model assumptions of independent observations were met. Model assumptions around multi co-linearity were also met as only one variable was continuous, meaning multi co-linearity could not arise. In all cases, GAMs were used to allow the non-linear relationship between age and outcome variables to be modelled by fitting a thin plate regression spline to age.

No outliers were removed from any analyses, as all variables considered were either categorical or had a pre-determined range. Participants were excluded from models if data was missing for any of the included variables. A p-value of less than 0.05 was considered statistically significant. Results of individual analyses examining demographics across MOOC iterations and characteristics associated with course completion were not adjusted for multiple comparisons as sample sizes are large and this study is descriptive not inferential. A Bonferroni adjustment for 6 comparisons was applied to the results for the models examining MOOC experiences and outcomes, as these 6 analyses were based on a single dataset and sample sizes in some comparison groups were small. Unadjusted p-values are reported, and unless otherwise noted these remained significant after Bonferroni adjustment and in the GAM adjusted for all other demographics. Interaction terms were not included in any models as these analyses aimed to determine the individual impact of each variable on the outcome.

All statistical analysis was completed in R Studio (R Studio version 1.2.5033; R version 3.5.3). GLMs were constructed using the stats package (version 3.5.3), GAMs were constructed using the mgcv package (version 1.8–31) and quantile regressions were constructed using the quantreg package (version 5.55). Analysis code is available in S1 File.

### Topic modelling

Participant’s free text responses to feedback survey questions were analysed based on the co-occurrence of words using topic modelling. Structural topic models were fitted with 5, 10, 15, 21 and 30 topics. The model with the greatest topic exclusivity and semantic coherence is reported for each analysis (feedback survey question). In all cases this was a 21-topic model. The topics in each model were explored further by manual thematic analysis to map topics to their respective meaningful underpinning themes [21]. The five most frequently mentioned topics for each model are reported.

Topic models were fitted in R Studio (R Studio version 1.2.5033; R version 3.5.3) using the stm package (version 1.3.3). Topic modelling code is available in S1 File.

## Results

### PDMOOC participants’ characteristics

A total of 105,280 individuals enrolled in the seven PDMOOC iterations run from July 2016 to May 2020; 11,059 of these individuals enrolled in the PDMOOC multiple times. The current study considers data provided by the 55,739 individuals who gave consent for their data to be used in research. Demographics, reasons for enrolling, and course progression and completion were analysed for participants who consented in the first PDMOOC iteration in which they enrolled (n = 51,388). Feedback survey data were analysed for participants who consented in the first PDMOOC iteration they completed (n = 52,282).

Among those who consented in their first PDMOOC enrolment, the mean age was 49 years (range = 18–100), with most participants being in their late 20s or early 50s. The majority of participants were female, worked in health care and social assistance sectors and had completed post-secondary education (Table 1). Participants came from 167 different countries across every habitable continent. Australia was the country in which the most participants lived (n = 36,453), followed by New Zealand (n = 3,429), Canada (n = 3,013), the United Kingdom (n = 1,908) and the United States of America (n = 1,026). The most commonly affirmed reasons for participating in the PDMOOC were “I want to reduce my risk of dementia”, “I want to improve my memory or thinking skills” and “I worry about my chances of getting dementia” (Table 2). Around one third of MOOC participants reported having a relative who was/is living with dementia (35.9%).

**Table 1:** Participant demographics.

	2016_07 (n = 4730)	2017_03 (n = 5979)	2018_05 (n = 2915)	2018_10 (n = 8509)	2019_05 (n = 7653)	2019_10 (n = 10235)	2020_05 (n = 11317)	Overall (n = 51338)
**Age**								
Mean (standard deviation)	49.7 (13.2)	50.1 (14.4)	48.9 (14.3)	48.6 (14.4)	47.9 (14.9)	50.8 (15.3)	47.0 (15.9)	48.9 (15.0)
Missing, n (%)	596 (12.6)	184 (3.1)	65 (2.2)	213 (2.5)	226 (3.0)	500 (4.9)	599 (5.3)	2383 (4.6)
**Gender, n (%)**								
Female	4178 (88.3)	5109 (85.4)	2526 (86.7)	7502 (88.2)	6686 (87.4)	8519 (83.2)	9582 (84.7)	44102 (85.9)
Male	536 (11.3)	858 (14.4)	381 (13.1)	985 (11.6)	933 (12.2)	1661 (16.2)	1684 (14.9)	7038 (13.7)
Missing or non-binary	16 (0.3)	12 (0.2)	8 (0.3)	22 (0.3)	34 (0.4)	55 (0.5)	51 (0.5)	198 (0.4)
**Occupation, n (%)**								
Health care and social assistance	3573 (75.5)	3962 (66.3)	2039 (69.9)	5794 (68.1)	4493 (58.7)	5452 (53.3)	7112 (62.8)	32425 (63.2)
Other occupation	926 (19.6)	1718 (28.7)	733 (25.1)	2300 (27.0)	1716 (22.4)	3225 (31.5)	4041 (35.7)	14659 (28.6)
Missing	231 (4.9)	299 (5.0)	143 (4.9)	415 (4.9)	1444 (18.9)	1558 (15.2)	164 (1.4)	4254 (8.3)
**Education, n (%)**								
Post-secondary education	3555 (75.2)	4956 (82.9)	2370 (81.3)	6904 (81.1)	5514 (72.1)	7254 (70.9)	9373 (82.8)	39926 (77.8)
Up to secondary education	1076 (22.7)	1023 (17.1)	545 (18.7)	1603 (18.8)	1055 (13.8)	1398 (13.7)	1690 (14.9)	8390 (16.3)
Missing	99 (2.1)	0 (0)	0 (0)	2 (0.0)	1084 (14.2)	1583 (15.5)	254 (2.2)	3022 (5.9)
**Country of residence income classification, n (%)**								
High-income	4582 (96.9)	5741 (96.0)	2798 (96.0)	8093 (95.1)	6990 (91.3)	9364 (91.5)	10138 (89.6)	47706 (92.9)
Low- and middle-income	141 (3.0)	227 (3.8)	108 (3.7)	402 (4.7)	638 (8.3)	842 (8.2)	1157 (10.2)	3515 (6.8)
Missing	7 (0.1)	11 (0.2)	9 (0.3)	14 (0.2)	25 (0.3)	29 (0.3)	22 (0.2)	117 (0.2)

**Table 2:** Percentages of participants responding in the affirmative to statements about the reasons for undertaking the Preventing Dementia MOOC.

Reason for participation	Percent agreement
I feel my memory or other thinking skills are getting worse	23.3
I think I may be getting dementia	4.0
I want information to take to my doctor	13.3
I want to improve my memory or thinking skills	66.6
I think I may inherit dementia from my parent or grandparent	18.6
I want to reduce my risk of dementia	68.8
I worry about my chances of getting dementia	42.1

Data are aggregated across six of the course iterations. Data were unavailable for the 2017 iteration.

### Changes in participants’ demographics across PDMOOC iterations

**Fig 1:**
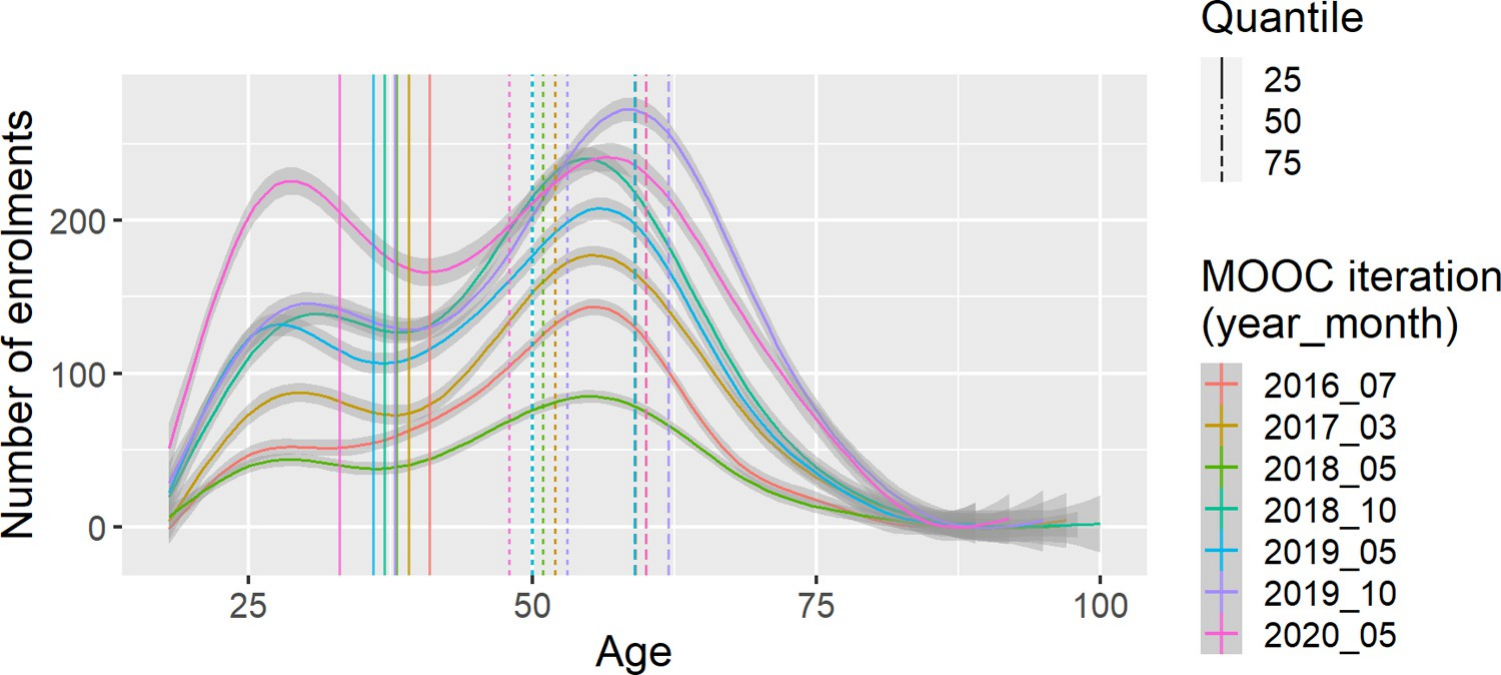
Number of participants with ages from 18 to 100 in each of the seven Preventing Dementia MOOC iterations (year-month). Age at which the 25^th^, 50^th^ and 75^th^ quantiles occurred for each iteration is shown by the vertical lines. The age at which the 25^th^ quantile occurred significantly decreased over time (iteration).

**Fig 2:**
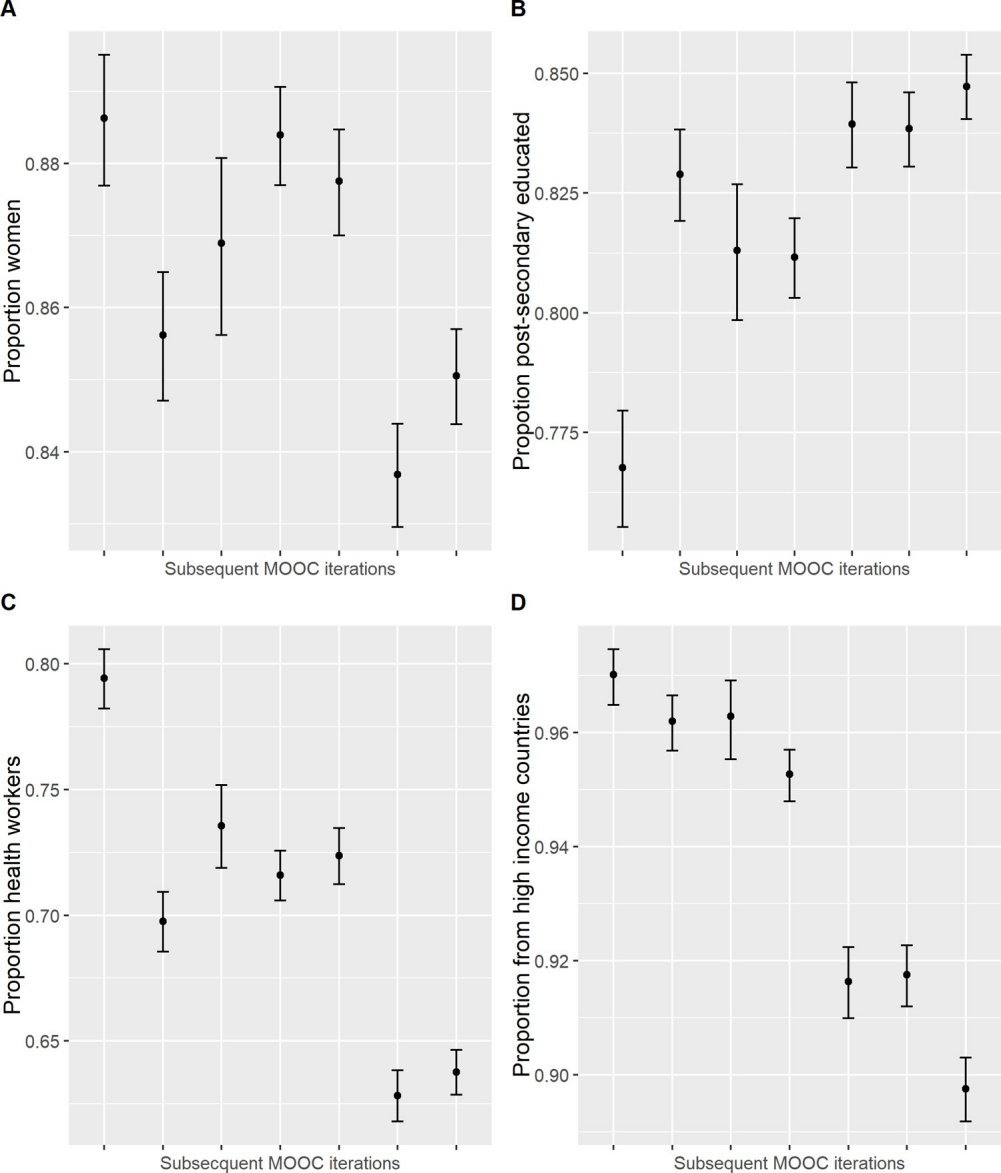
Proportions of participants who were female (A), had completed post-secondary education (B), worked in health care and social assistance sectors (C), and lived in high-income countries (D) in each of the seven Preventing Dementia MOOC iterations (year-month). Increasing representation over time is demonstrated for men (A), those with post-secondary qualifications (B), those working outside health care and social assistance, and those in low- and middle-income countries (D).

The reach of the PDMOOC has changed over time. The number of people enrolling in the PDMOOC has increased, from 11,394 in the first iteration in July 2016 to 25,838 in the May 2020 course offering. Participant’s age, gender, education level, occupation and country of residence varied significantly across MOOC iterations (S1 Table). Generally, there was a larger proportion of younger participants in later iterations of the MOOC, with the age at which the 25^th^ quantile occurred tending to decrease across iterations, while the age at which the 50^th^ and 75^th^ quantile occurred remained relatively consistent (Fig 1 and S1 Table) and mean age fluctuated between 47 and 51 years (Table 1). The proportion of female participants tended to decrease across iterations (Fig 2A and S1 Table) suggesting more males were attracted to undertaking the course in more recent iterations. The proportion of participants with a post-secondary education tended to increase across iterations (Fig 2B and S1 Table), while the proportion of participants employed in health care and social assistance tended to decrease (Fig 2C and S1 Table). The proportion of participants from high-income countries tended to decrease across iterations, with notably lower proportions in the three most recent iterations suggesting increased reach into low- and middle-income countries over time (Fig 2D and S1 Table). These associations remained significant after adjusting for all other demographics under consideration, with the exception of some for gender and country of residence (S1 Table).

### PDMOOC participants’ progression and completion

**Fig 3:**
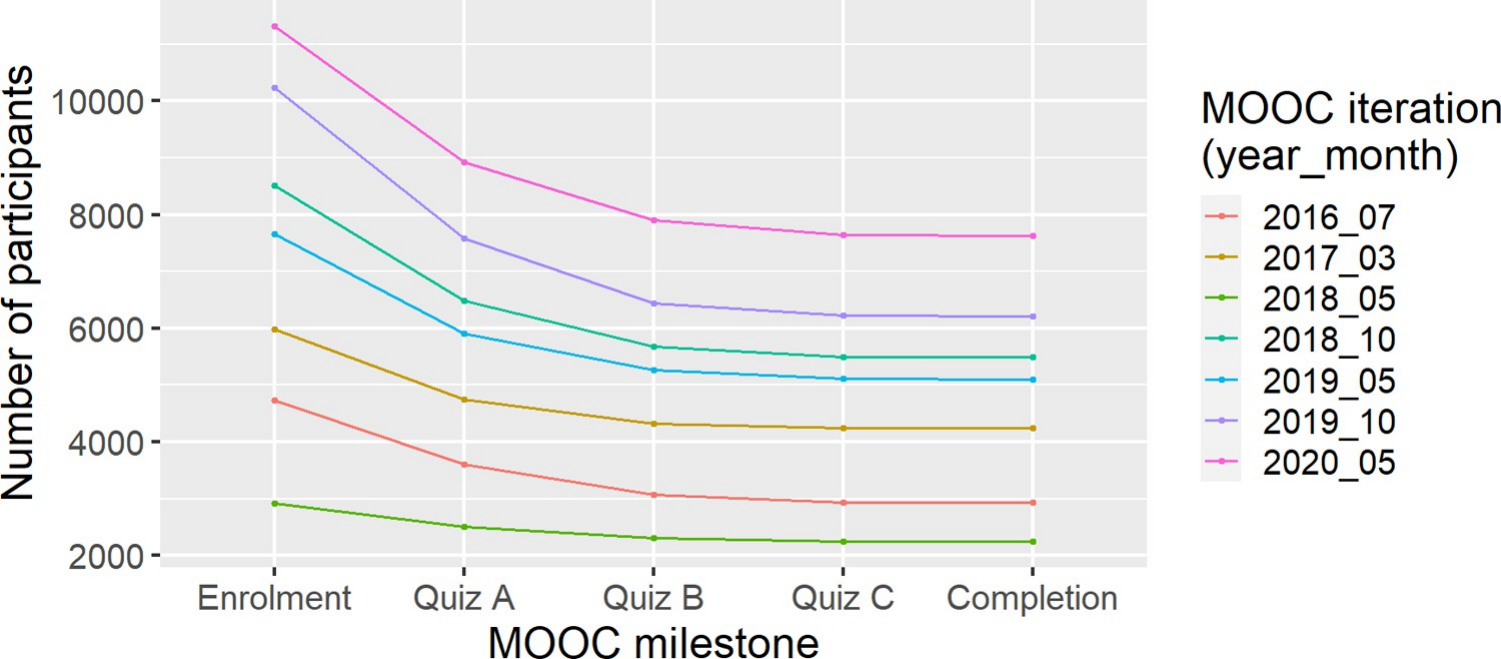
Numbers of participants participating at each stage of the Preventing Dementia MOOC for each of the seven iterations of the course (2016_07, 2017_03, 2018_05, 2018_10, 2019_05, 2019_10, 2020_05). The largest attrition occurred during the first module for all iterations.

Most participants completed the PDMOOC in their first enrolment (65.9%). Most participants who did not complete the MOOC (66.1%) did not attempt the first quiz, demonstrating low levels of engagement with the MOOC from the beginning among non-completers (Fig 3). This trend continued throughout the stages of the MOOC; 93.5% of non-completers did not attempt quiz B and 99.7% of non-completers did not attempt quiz C.

**Fig 4:**
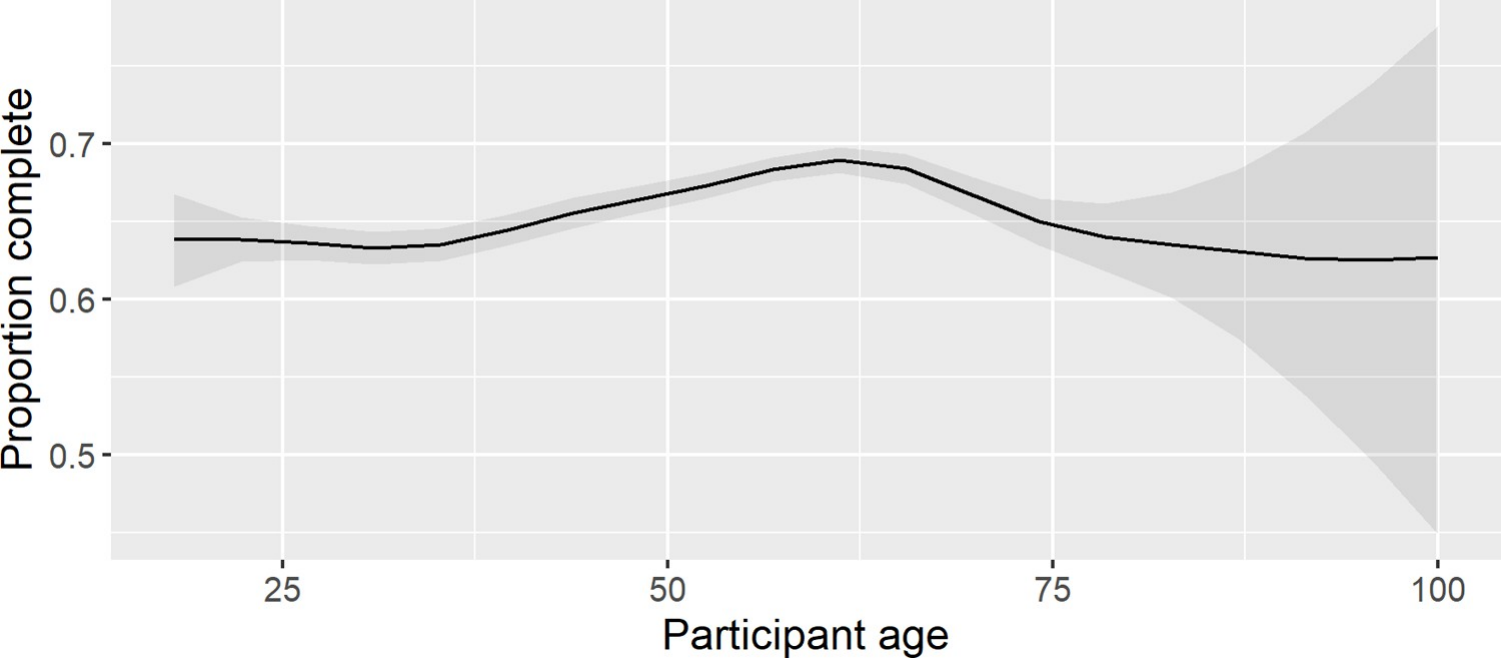
Proportion of participants at different ages who completed the Preventing Dementia MOOC.

**Fig 5:**
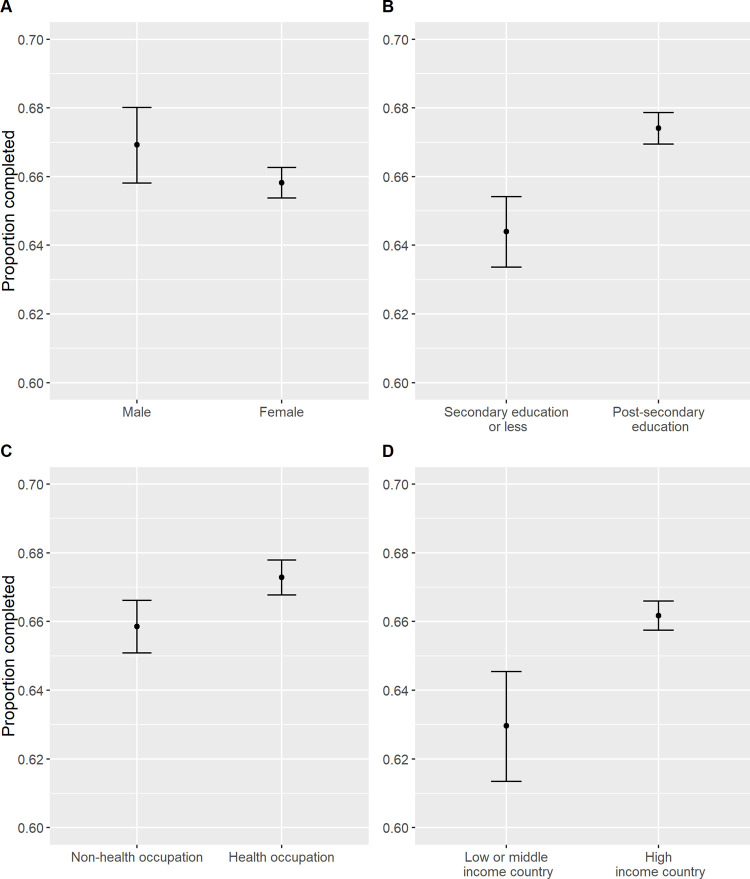
Proportions of participants who completed the Preventing Dementia MOOC by gender (A), highest level of education (B), work in health care and social assistance or other sectors (C), and wealth of country of residence (D) across all seven Preventing Dementia MOOC iterations.

Participants from some demographic groups were more likely to complete the MOOC than others. Age was significantly associated with MOOC completion; participants aged between 50 and 70 were most likely to complete the MOOC (Fig 4 and S2 Table). Participants were significantly more likely to complete the MOOC if they had a male gender, a post-secondary education, a health-related occupation, or a high-income country of residence (Fig 5 and S2 Table). The proportion of participants completing the MOOC varied significantly across iterations, however there was no consistent trend over time (S2 Table).

Participants reasons for enrolling in the MOOC were also associated with MOOC completion. Participants were significantly more likely to complete the MOOC if they responded affirmatively to the statements “I want to reduce my risk of dementia” and “I want to improve my memory or thinking skills”, and significantly less likely to complete the if they responded affirmatively to the statements “I feel my memory or other thinking skills are getting worse” or “I want information to take to my doctor” (S2 Table).

### PDMOOC participants’ experiences

Participants were asked to reflect on their MOOC experiences in the feedback survey offered after course completion. The question "What was the best thing about the Preventing Dementia MOOC?" elicited text responses from 12,076 participants (S3 Table). The most common topic arising in these responses was increased knowledge and positivity around dementia risk reduction: “*Gaining more knowledge and that enables me to feel more positive about dealing with modifiable risks of developing dementia”*. Other frequently mentioned topics included ease of navigation and comprehension: *“Easy to navigate site*, *videos were easy to understand*, *and informative”*; the novelty of the information presented: *“The enjoyment of learning something new”;* and the quality of the information and presentations: *“The information was presented in a most professional but relaxed way*, *encouraging participation*. *The information provided was interesting and which we could relate to”*.

Participants were also asked “What was the worst thing about the Preventing Dementia MOOC?” and 10,436 participants provided a text response to this question (S3 Table). Three of the most common topics arising in these responses related to a lack of negative features: *“Nothing to fault”*, “*I don’t have anything to add here*. . . . *can’t think of anything that bothered me”*. Other frequently mentioned topics included difficulty finding time to do the MOOC during the limited timeframe for completion: *“the time restrictions*. *Due to my busy life*, *a couple of extra weeks to do it at my own pace would have fit into my life better”* and a preference for written information rather than video presentations: *“Some of the lengthy videos*, *however I changed to reading the transcripts and found that I was able to concentrate on the material on offer a lot better”*.

97% of participants agreed or strongly agreed with the statement “I was satisfied with my MOOC learning experience” (n = 17397). Men were significantly less likely to report course satisfaction than women, and non-health workers were significantly more likely to report course satisfaction than health workers (S4 Table).

### PDMOOC participants’ self-reported outcomes

The feedback survey asked participants to respond to statements relating to the outcomes they obtained from participating in the PDMOOC. More than 98% of participants either agreed or strongly agreed (affirmed) that, as a result of the course, their understanding of dementia prevention had improved, and they would recommend the course to others (Table 3). Most also agreed the PDMOOC provided the information needed to reduce dementia risk and had an impact on their behaviour and lifestyle. Around three-quarters agreed they had already applied the knowledge gained from the MOOC.

**Table 3:** Percentages of participants who agreed or strongly agreed with statements about possible outcomes from undertaking the Preventing Dementia MOOC across the seven course iterations.

Feedback survey statement	Percent agreement	Total number of responses
My understanding of dementia prevention has improved	98.4	16,554
The MOOC has given me the information I need to reduce my dementia risk	94.5	16,514
The MOOC has had an impact on my behaviour and lifestyle choices	85.8	16,530
I have already applied the knowledge I have gained from the MOOC	73.0	16,520
I would recommend the MOOC to others	98.7	16,522

Affirmation of the MOOC outcome statements was significantly related to age. The proportion of participants affirming the statement “My understanding of dementia prevention has improved” increased from age 25 to age 50, and from age 50 to age 70, then decreased from age 70 to age 90 (S5 Table). The proportion of participants affirming the four remaining outcome statements increased from age 25 to age 50, then decreased from age 50 to age 70, and decreased again from age 70 to age 90 (S6–S9 Tables).

Men were significantly less likely than women to affirm the MOOC outcome statements “The MOOC has given me the information I need to reduce my dementia risk” (S6 Table) and “The MOOC has had an impact on my behaviour and lifestyle choices” (S7 Table).

Health workers were significantly more likely than non-health workers to affirm the statements “The MOOC has had an impact on my behaviour and lifestyle choices” (S7 Table) and “I have already applied the knowledge I have gained from the MOOC” (S8 Table).

Participants who had not completed post-secondary education were significantly more likely than those who had to affirm the statement “The MOOC has had an impact on my behaviour and lifestyle choices” (S7 Table).

Participants from low- and middle-income countries were significantly more likely to affirm the statements “The MOOC has had an impact on my behaviour and lifestyle choices” (S7 Table) and “I have already applied the knowledge I have gained from the MOOC” (S8 Table) than participants from high-income countries, but significantly less likely to affirm the statement “I would recommend the MOOC to others” (S9 Table).

The feedback survey statement “If you have already applied your MOOC learning, please tell us how” elicited text responses from 8,471 participants (S3 Table). The most common topic arising in these responses related to changes in diet: *“diet modification*. *I’m substituting butter for olive oil and going to eat less red meat and more beans”*. Other frequently mentioned topics included increased physical activity: “*Increased my physical exercise level”*; increased cognitive activity: *“I am more intently looking into educational activities and other things I enjoy doing everyday to improve my cognitive reserves*!*”*; and viewing dementia risk reduction as an additional motivating factor to maintain a healthy lifestyle: *“I have always watched my diet and exercise but after finishing the course I am more aware of why I need to keep on track*. *As I always believed–mind and body*. *It is not just one thing we do*, *it is all connected*.*”* Tertiary prevention was also a common topic: “*Working in an aged care facility is very challenging especially that you have to care for residents with dementia*. *This course help me to understand the people with dementia and it guides me on how to provide quality care to them”*.

## Discussion

The findings presented here demonstrate that the first seven iterations of the Preventing Dementia MOOC reached and had positive impacts on tens of thousands of people all over the world with an interest in dementia risk reduction. The course is now offered biannually with enrolment numbers per course consistently around 20,000.

### Who undertakes the PDMOOC?

PDMOOC participants who consented to research (henceforth called ‘participants’) were diverse, aged from 18 to 100, from 167 different countries, and had a range of previous education and occupations. However, females, the well-educated, those working in health care and social assistance, and those living in high-income countries were over-represented. Health information is typically more often sought by women, those with greater levels of education and those from higher socioeconomic backgrounds [22]. Health research, including dementia prevention initiatives, typically attracts more women than men [23–25], and those with higher levels of education [23, 25–28]. The proportion of male PDMOOC participants rose over time, but remains low at around 15%, despite the positive trend. Other, possibly related, changes in participant demographics over time were observed. The proportion of participants with higher than secondary level education increased slightly over time. This may be related to the concurrent reduction in the proportion of participants working in health care and social assistance. Many participants in this occupational category are aged care workers who enrol in the PDMOOC after undertaking the Understanding Dementia MOOC and often have relatively low levels of formal education. Hence, as the PDMOOC attracts more people with a general interest in dementia prevention, these people may be less likely to work in aged care but more likely to be highly educated.

Participants from low- and middle-income countries increased over time, but representation remains low at around 10%. Those from affluent countries are also over-represented among enrolees and completers of other MOOCs [29]. As the PDMOOC is delivered by an Australian university and the bulk of course promotion is conducted in Australia, it is unsurprising that most participants reside in Australia. As the course is delivered in English, the predominance of participants from English speaking countries is also expected. However, this highlights current inequalities in access to dementia prevention knowledge and reflects the larger issue of lack of data related to dementia risk profiles and dementia prevention intervention efficacy in non-Western regions [2, 7]. Such data could inform adaptation of existing interventions like the PDMOOC for specific populations in culturally appropriate ways. In summary, the under-representation of men, those with lower formal education, those working outside the health sector, and people in low- and middle-income countries remains an issue for optimising the reach of the PDMOOC, although the positive trends in some demographics are encouraging.

The mean age of PDMOOC research participants varied by just a few years across the seven iterations, at around 49 years. The largest peak in the distribution of participant ages occurred in the late fifties, indicating many PDMOOC participants are middle-aged, a time in life when important dementia risk factors may exert their greatest influence [1, 2]. As explained in the PDMOOC, exposure to risk factors such as hypertension, hypercholesterolaemia and obesity during midlife may be associated with the greatest dementia risk [1, 2, 30]. Another peak in the age distribution occurred in the late twenties and the age at which the 25^th^ quantile occurred reduced significantly over time. Anecdotal evidence from participants suggests that younger PDMOOC participants in recent iterations could be due to dementia prevention knowledge being encouraged across university degree programs.

### What motivates interest in the PDMOOC?

Participants endorsed wanting to reduce their dementia risk and improve their cognitive skills as major motivating factors for undertaking the course. Similar priorities have been reported for visitors to a dementia risk reduction website [25]. Motivation to change behaviour toward dementia risk reduction is increased by exposure to information [31], so understanding motivating factors for paying attention to the information in the first place is important for successful dementia prevention in the population.

Having a family history of dementia may also be a motivating factor for people to learn about prevention [32, 33]. More than one third of participants reported having a family member with dementia and around one in five participants agreed thinking they may inherit dementia was a reason for undertaking the PDMOOC. Greater belief in a mostly genetic cause of dementia and feelings of hopelessness that dementia cannot be prevented has been identified among those with a family connection [32, 33]. A family history of dementia could motivate some individuals by increasing concern about one’s own risk, whilst demotivating others who perceive that nothing can be done [32]. Providing information regarding the contributions of and interplay between genetic and modifiable health and lifestyle factors is essential for this audience, and the PDMOOC does this.

Whilst these data are preliminary and we require further research to unpack the nuances around course motivation, they suggest that the target audience, those wishing to reduce their dementia risk, are attracted to the PDMOOC.

### Who completes the PDMOOC?

Participants’ progression through the PDMOOC followed a typical course for MOOCs [29], with most attrition occurring in the first module. PDMOOC completion rates are not typical, however, and far exceed the low completion rate of MOOCs seen in general [29]. Course completion is not the only measure of success of a MOOC and is likely not the best measure of successful learning for participants. However, it does provide a first step in understanding the ability to engage participants and have them persist through the course [34].

PDMOOC completion was significantly associated with some demographic and motivational factors, although these effects were small. Gender did not affect likelihood of completion, but age did, consistent with previous reports of predictors of MOOC completion [34, 35]. PDMOOC completion was most likely for participants aged between 50 and 70 years, suggesting a stronger interest in the information provided among this age group. They are an important key target for dementia prevention given that the brain pathology causing dementia develops over the 20 years prior to symptom onset [36] and that delaying the average age of dementia onset would reduce the prevalence and impact of the condition [7]. Further research is needed to understand why people in the 50 to 70 age range are more likely to complete the PDMOOC compared to other age groups, and how others may be encouraged to complete.

Having a post-secondary education, working in a health-related occupation and living in a high-income country were also associated with significantly greater likelihood of completing the PDMOOC. However, only 1% more of those working in health completed than non-health workers, suggesting little material difference according to occupation. For education and country of residence, the group difference in completion rate was just 3%. Higher formal education levels have predicted MOOC completion in some previous studies [34, 35] but not others [37]. Given that lower educational attainment itself is one of the modifiable risk factors for dementia [1], it is appropriate to ensure that educational initiatives promote accessibility and accommodate those from lower educational backgrounds to maximise completion. Similarly, dementia prevention initiatives targeting participants from low- and middle-income countries need to be culturally appropriate in design in order to enhance completion [7], although the current study indicates that PDMOOC participants from these regions were only slightly less likely to complete the course. Finally, more participants who reported being motivated to undertake the course by cognitive health and dementia risk reduction completed the PDMOOC (5% difference), whereas those motivated by concerns of existing cognitive decline or imminent dementia were less likely to complete the course (2% difference). This may be explained by the fact that the PDMOOC targets principles of primary rather than secondary or tertiary prevention. Those driven to learn about ways to adapt their lifestyle prior to negative changes in their cognitive health may be more likely to complete than those who believe such changes are already occurring.

Retention of participants and their adherence to dementia risk reduction interventions is required for optimal success. Adherence to intensive 2–3 year multidomain dementia risk reduction interventions was reported to be between 19% and 61% (adherence defined as attending at least 66% of sessions), with adherence decreasing with increasing burden of the intervention for participants [38]. The relatively high PDMOOC completion rate for participants in this study (66%) suggests good adherence to this brief educational intervention. A limitation of the current study is the lack of feedback data from participants who did not complete the course, including reasons for non-completion. Further research is needed to understand why some participants drop-out or do not fully engage in MOOCs [34] and other intervention strategies [38], as well as how engagement and completion can be enabled.

### Course satisfaction and outcomes

Feedback from over 20,000 completing participants suggests very high levels of satisfaction with the PDMOOC. Although men and those working in health were significantly less likely to agree they were satisfied with their MOOC experience, only a few percent of these participants did not affirm satisfaction, and differences in the proportion affirming satisfaction between genders and between occupation categories were 1% or less. The vast majority of participants also affirmed that the MOOC had given them the information needed to reduce their dementia risk and that they would recommend the course to others. Men and those over 50 were significantly less likely to affirm these outcomes, but again differences in proportions were very small. Those in low-and middle-income countries were significantly less likely to say they would recommend the PDMOOC. It may be that language and cultural barriers prevent some wanting to tell others about the course, but again only a few percent of participants in these countries did not affirm the course recommendation statement.

When asked about the best thing about the course, participants frequently reported increased knowledge and positive feelings that risk can be reduced. These outcomes align with PD MOOC participants’ motivation to reduce risk and improve cognition. This is also consistent with themes previously identified for web-based health intervention participants [39] and visitors to a dementia prevention website [25]. Several studies have identified that greater knowledge is associated with increased motivation and intention to reduce dementia risk [31, 40, 41], suggesting that providing knowledge about dementia risk and prevention may motivate behaviour change. Other encouraging research suggests that intentions predict behaviour 50% of the time [42].

Most participants agreed that completing the PDMOOC immediately influenced their behaviour and lifestyle choices in some capacity. There were some demographic differences, however. Those older than 50, men, those working outside health, those with post-secondary education and those in high-income countries were less likely to agree the MOOC had an impact on their behaviour and lifestyle choices. Apart from gender and education, the same differences occurred for agreeing they had already applied the knowledge gained. While significant, the differences in proportions affirming these outcomes were small (2 to 6%). It may be that these demographic differences represent retired people from advantaged backgrounds who are already leading healthy lifestyles and therefore do not believe they need to further modify their lifestyle. This is a demographic that is highly represented in the PDMOOC. Nonetheless, across all demographic groups, high proportions of participants affirmed impacts on their behaviour and lifestyle.

The most commonly reported changes were to diet, physical activity and cognitive activity. This is consistent with the dementia risk factors that are most familiar to the public [8] and were of most interest in other brain health interventions [25, 43]. It may also reflect perceptions around control of those risk factors as they are immediately actionable and require minimal input from external sources. This suggests for some participants, knowledge is translated into behaviour change. This may have important implications for dementia prevention given even small changes at the population level could achieve a significant reduction in dementia incidence [7, 44]. While we cannot assume these outcomes reported by only those who completed the course and the feedback survey are representative of all PDMOOC participants, this does suggest the course is providing many participants with increased knowledge and motivation to reduce their dementia risk and enabling behaviour change. Further research is needed to objectively measure behaviour change and its sustainability following educational interventions such as the PDMOOC.

The domains of risk people are least aware of are the vascular factors such as hypertension [8] and despite large sections of the PDMOOC content dedicated to these, participants are perhaps not taking the message on board, or may be already having regular health checks and managing these risk factors, or may be more inclined to address and/or report addressing the lifestyle factors they are more familiar with and have easier control over. More research is needed to discover why people might focus more on particular risk factors, and whether the PDMOOC can be modified to overcome this possible bias.

Aspects of the PDMOOC itself were also reported to contribute to course satisfaction, such as quality of presentation and ease of navigating the platform. Trustworthiness, user-friendliness, and up-to-date information were reported as the most important facilitators for the use of an online brain health program [43]. Participants also commonly reported enjoying the novelty of the information provided and learning something new. That participants found the information novel is consistent with the low levels of knowledge about dementia and its prevention that are prevalent in the general public [8, 10] and previous findings of increased self-reported knowledge post-educational intervention [25]. Participants enjoyed increasing their knowledge which suggests the PDMOOC is providing important information in an engaging way.

High participant satisfaction (80% or more) has been reported in previous evaluations of health and medicine MOOCs [35, 45]. A review of internet-based multidomain brain health programs found generally positive user experiences [44], suggesting that this mode of delivery is appropriate. Some participants reported negative issues such as technical difficulties and struggling to find enough time to complete the PDMOOC, consistent with previous reports for other online interventions [44, 46]. Some participants reported a preference for written information rather than video presentations, which is accommodated in the PDMOOC through the provision of video transcripts. Access to sufficiently up-to-date technology and/or the skills to use it remains a barrier for some MOOC participants.

Mainstream and social media are common sources of health information, but headlines are often misleading and health and medical research findings over-simplified or exaggerated. A ‘panic-blame’ framework has been identified in media reporting of dementia and risk, which catastrophises dementia and treats it as preventable, leading to people with dementia feeling or being blamed for not doing enough to avoid the condition [47, 48]. Public engagement (experts engaging with the community) is needed to ensure accurate messages are conveyed that educate the masses in a way that reduces dementia stigma and enables risk reduction. There is a paucity of data on the impacts of public engagement in dementia and its prevention [47]. The few studies evaluating efforts to raise awareness of dementia risk reduction such as engaging with local communities [49], local public awareness campaigns [28, 31], educational websites [23, 25, 50], and the PDMOOC, report some difficulties reaching and recruiting people. However, among those who did engage in these initiatives, there was high participant satisfaction and improvements in awareness and motivation to change dementia risk-related behaviour, highlighting the potential positive impact of such public health interventions.

## Conclusions

This evaluation demonstrates that the PDMOOC is accessible, informative, and motivating for the majority of participants who completed the course and provided feedback data. The PDMOOC is freely and globally available and has reached over 100,000 people around the world in five years, with participants finding the course gave them the information needed to reduce their dementia risk and many acting on that. Dementia prevention is a global priority, meaning that scalable and sustainable public health interventions are required [1, 7, 14]. While equity of access and uptake by those with likely higher dementia risk need to be addressed, the PDMOOC can make a significant contribution to the public health response to dementia. By providing knowledge on a global scale that participants are able to translate into action to reduce their dementia risk, the PDMOOC may contribute to ameliorating the growing prevalence and impact of dementia.

## Supporting information

S1 File Statistical and topic modelling analysis code. (PDF)

S1 Table Associations between PDMOOC iteration and participant demographics. (DOCX)

S2 Table Associations between completion of the PDMOOC and participant demographics, PDMOOC iteration, having a family history of dementia, and reasons for undertaking the PDMOOC. (DOCX)

S3 Table The five most common themes arising in the structural topic models for the three open-ended feedback questions. (DOCX)

S4 Table Associations between affirmation of the statement “I was satisfied with my MOOC learning experience” and participant demographics. (DOCX)

S5 Table Associations between affirmation of the statement “My understanding of dementia prevention has improved” and participant demographics. (DOCX)

S6 Table Associations between affirmation of the statement “The MOOC has had an impact on my behaviour and lifestyle choices” and participant demographics. (DOCX)

S7 Table Associations between affirmation of the statement “The MOOC has given me the information I need to reduce my dementia risk” and participant demographics. (DOCX)

S8 Table Associations between affirmation of the statement “I have already applied the knowledge I have gained from the MOOC” and participant demographics. (DOCX)

S9 Table Associations between affirmation of the statement “I would recommend the MOOC to others” and participant demographics. (DOCX)
