# Supplementary material for: Educating the masses to address a global public health priority: The Preventing Dementia Massive Open Online Course (MOOC)
Source: PLoS One. 2022 May 4;17(5):e0267205. doi: 10.1371/journal.pone.0267205 (PMC9067672; doi:10.1371/journal.pone.0267205)
Supplement: S1 File — (PDF) [file pone.0267205.s001.pdf]

Packages and versions used:

$`package:reshape2` version 1.4.4

[1] "melt"

$`package:table1` version 1.2

[1] "table1"

$`package:base`

[1] "as.character" "as.factor" "as.numeric" "cbind" "cut" "data.frame" "duplicated" "exp" "expand.grid" "factor" "is.na"

[12] "jitter" "lapply" "length" "list" "load" "log" "matrix" "ncol" "nrow" "paste0" "print"

[23] "rep" "return" "round" "sample" "save" "scale" "seq" "set.seed" "summary" "unlist"

$`package:dplyr` version 1.0.4

[1] "bind_rows" "count" "left_join" "na_if" "recode" "sample_n" "semi_join" "tally" "filter" "group_by" "mutate" "select" "rename"

$`package:emmeans` version 1.4.6

[1] "emmeans"

$`package:ggplot2` version 3.3.5

[1] "aes" "element_blank" "element_text" "facet_wrap" "geom_bar" "geom_errorbar" "geom_jitter" "geom_line"

[9] "geom_point" "geom_ribbon" "geom_smooth" "geom_vline" "ggplot" "ggsave" "guide_legend" "guides"

[17] "labs" "scale_x_discrete" "scale_y_continuous" "theme" "xlab" "xlim" "ylab" "unit"

$`package:ggpubr` version 0.4.0

[1] "ggarrange"

$`package:graphics` version 3.5.3

[1] "plot"

$`package:grDevices` version 3.5.3

[1] "png"

$`package:knitr` version 1.28

[1] "kable"

$`package:mgcv` version 1.8-31

[1] "gam" "s"

$`package:oddsratio` version 2.0.1

[1] "or_gam" "or_glm"

$`package:purrr` version 0.3.4

[1] "map" "map_chr"

$`package:quantreg` version 5.55

[1] "rq"

$`package:stats` version 3.5.3

[1] "binomial" "complete.cases" "confint" "glm" "model.frame" "predict" "qqline" "qqnorm" "model.matrix"

$`package:stm` version 1.3.3

[1] "findThoughts" "manyTopics" "prepDocuments" "textProcessor"

$`package:stmQuality` version 0.0.0.9000

[1] "extractFit"   "findThoughts0"

$`package:tidyr` version 1.0.2

[1] "nest"   "pivot_longer" "pivot_wider" "replace_na"

$`package:tidytext` version 0.2.4

[1] "unnest_tokens"

$`package:tm` version 0.7-7

[1] "stopwords"

$`package:utils` version 3.5.3

[1] "read.csv" "write.csv"

$`package:maps` version 3.3.0

[1] "iso3166"

load these packages

```{r}
library(reshape2)
library(table1)
library(dplyr)
library(emmeans)
library(ggplot2)
library(ggpubr)
library(graphics)
library(grDevices)
library(knitr)
library(mgcv)
library(oddsratio)
library(purrr)
```

```
library(quantreg)
library(stats)
library(stm)
library(stmQuality)
library(tidyr)
library(tidytext)
library(tm)
library(utils)
library(maps)
```

Load data

```{r}
load("combined_first_enrolment")
```

Produce a demographic table for enrolments and completions

This section uses each user's first enrolment, as this gives the most accurate completion:non-completion ratio

```{r}
d <- combined_first_enrolment
d$completed <- as.factor(d$completed) %>%
  dplyr::recode("0" = "Non-complete",
        "1" = "Complete")
```

```
d$Gender <- as.factor(d$gender)
d$Occupation <- as.factor(d$anzsic_div_q_occ) %>%
  dplyr::recode("1" = "Health occupation",
        "0" = "Other occupation")
d$Education <- as.factor(d$post_secondary_edu) %>%
  dplyr::recode("1" = "Post-secondary education",
        "0" = "Lower level of education")
d$MOOC <- as.factor(d$mooc)
d$Quiz_A <- d$quiz_1_percent
d$Quiz_B <- d$quiz_3_percent
d$Quiz_C <- d$quiz_4_percent
d$Age <- d$age

t3 <- table1::table1(~Age + Gender + Occupation + Education + Quiz_A + Quiz_B + Quiz_C + MOOC | completed, data = d,
      render.continuous = c("Mean (standard deviation)" = "Mean (SD)"),
       topclass="Rtable1-zebra")
t3


```

Analyse and graph enrolment data by country and by GDP per capita of country of origin

Again, use each participant's first enrolment for consistency

```{r}
load("combined_first_enrolment")
load("combined_first_completion")

all_users_quiz <- combined_first_enrolment

#country data
```

```
a <- all_users_quiz %>% dplyr::group_by(country) %>% tally()

a <- a %>% dplyr::filter(!is.na(country))

#this was inspected to give numbers of participants from each country, but 2 letter codes are hard to interpret


#country names and three letter codes added to dataset

country_codes <- read.csv("country_codes.csv")

country_codes$country <- as.character(country_codes$country)

country_codes %>% dplyr::filter(duplicated(country)) #8 duplicates removed

country_codes <- country_codes %>% dplyr::filter(!duplicated(country))


countries <- left_join(a, country_codes)


nrow(countries) #people from 171 different countries have enrolled in the mooc

nrow(countries)/nrow(country_codes)*100

#people from 67% of all counties have enrolled in the MOOC


b <- countries %>% dplyr::group_by(continent) %>% tally()

b

#6 continents repressented - every continent except Antarctica


#Include a continuous varible based on country (GDP per capita) for further analysis. Two many levels in country for it to provide useful comparisons.

#GDP data from world bank https://data.worldbank.org/indicator/NY.GDP.PCAP.CD?view=map

gdp_per_capita <- read.csv("gdp_per_capita.csv")


#prepare to join GDP per capita to other data using 2 letter country codes

names <- iso3166

names <- names %>%

  dplyr::mutate(country_name = mapname) %>%

  dplyr::select(country_name, a2, a3)
```

```
gdp_per_capita <- gdp_per_capita %>%
  dplyr::mutate("a3" = Country.Code) %>%
  dplyr::select(a3, X2018)

gdp_per_capita <- left_join(names, gdp_per_capita) %>%
  dplyr::mutate(country = a2) %>%
  dplyr::select(country, X2018)

#inspect GDP per capita across mooc iterations
gdp_mooc <- all_users_quiz %>% dplyr::select(mooc, country)
gdp_mooc$mooc <- gdp_mooc$mooc %>%
  dplyr::recode("2016_07" = "1",
        "2017_03" = "2",
        "2018_05" = "3",
        "2018_10" = "4",
        "2019_05" = "5",
        "2019_10" = "6",
        "2020_05" = "7")
gdp_mooc <- gdp_mooc %>%
  dplyr::group_by(mooc, country) %>%
  tally()

gdp_mooc <- left_join(gdp_mooc, gdp_per_capita)

ggplot(gdp_mooc, aes(x = mooc, y = X2018)) +
  geom_jitter(aes(colour = log(n)))
#possibly more people from lower GDP per capita countries in later iterations

#add a column showing the imcome classification of each country using the current world bank classifications from 10.2.2021 (based on GNI from 2019)
income_categories <- read.csv("income_categories.csv")
```

```
income_categories <- income_categories %>% dplyr::select(a3, country_income_group)

income_categories <- left_join(names, income_categories) %>%
  dplyr::mutate(country = a2) %>%
  dplyr::select(country, country_income_group)

#remove duplicates
income_categories <- income_categories %>% dplyr::filter(!duplicated(country))

#create a data set for further analysis
d1 <- all_users_quiz %>%
  dplyr::select(user_id, mooc, country)

b <- gdp_per_capita %>% dplyr::filter(duplicated(country))
c <- semi_join(gdp_per_capita, b) #some countries are included in this dataset twice, but all duplicates are equivalent
gdp_per_capita <- gdp_per_capita %>% dplyr::filter(!duplicated(country))

d1 <- left_join(d1, gdp_per_capita)

#rescale X2018 to avoid "infintie X" error when calling summary on models
d1 <- d1 %>% dplyr::mutate(gdp_scaled = scale(X2018))

combined_first_enrolment_gdp <- d1

combined_first_enrolment_gdp <- combined_first_enrolment_gdp %>%
  dplyr::select(user_id, X2018, gdp_scaled) %>%
  dplyr::mutate(gdp_country = X2018) %>%
  dplyr::select(-X2018)
```

```
combined_first_enrolment <- left_join(combined_first_enrolment, combined_first_enrolment_gdp)

combined_first_enrolment <- left_join(combined_first_enrolment, income_categories)


#order factor levels

combined_first_enrolment$country_income_group <-
factor(combined_first_enrolment$country_income_group, levels = c("Low income", "Lower middle
income", "Upper middle income", "High income"))



#plot the distribution of enrolments across country income categories

ggplot(combined_first_enrolment, aes(x = country_income_group)) + geom_bar()


#save combined_first_enrolment with country_income_group

save(combined_first_enrolment, file = "combined_first_enrolment")



#add income categories into combined_first_completion too

gdp_mooc_comp <- combined_first_completion %>%

  dplyr::select(user_id, mooc, country)

d1 <- left_join(gdp_mooc_comp, gdp_per_capita)


d1 <- d1 %>% dplyr::mutate(gdp_scaled = scale(X2018))


combined_first_completion_gdp <- d1


combined_first_completion_gdp <- combined_first_completion_gdp %>%

  dplyr::mutate(gdp_country = X2018) %>%

  dplyr::select(-X2018)
```

combined_first_completion_gdp <- left_join(combined_first_completion_gdp, combined_first_enrolment_gdp)

combined_first_completion_gdp <- left_join(combined_first_completion_gdp, income_categories, by = "country")

combined_first_completion_gpd <- combined_first_completion_gdp %>%

  dplyr::select(-gdp_scaled, -gdp_country)

combined_first_completion <- left_join(combined_first_completion, combined_first_completion_gdp)

save(combined_first_completion, file = "combined_first_completion")

```

Explore data - deomgraphics across iterations

```{r}

load("combined_first_enrolment")

#recode gender for table

combined_first_enrolment$gender <- na_if(combined_first_enrolment$gender, "other")

combined_first_enrolment$gender <- factor(combined_first_enrolment$gender)

#Combine relevent data

d1 <- combined_first_enrolment %>%

  dplyr::select(user_id, age, gender, post_secondary_edu, anzsic_div_q_occ, mooc, gdp_country, completed, country_income_group) %>%

  dplyr::mutate(edu = dplyr::recode(post_secondary_edu, yes = "1", no = "0")) %>%

  dplyr::mutate(mooc = as.factor(mooc),

            gender = gender,

            edu = post_secondary_edu,

            occ = as.character(anzsic_div_q_occ)) %>%

```
  dplyr::select(user_id, age, gender, edu, occ, mooc, gdp_country, country_income_group)

d2 <- d1

#Create a demographic table across different MOOC iterations
d <- d2
d$mooc <- as.factor(d$mooc)
d$Gender <- as.factor(d$gender) %>%
  recode("female" = "Female",
        "male" = "Male")
d$Occupation <- as.factor(d$occ) %>%
  dplyr::recode("1" = "Health occupation",
        "0" = "Non-health occupation")
d$Education <- as.factor(d$edu) %>%
  dplyr::recode("1" = "Post-secondary education",
        "0" = "Lower level of education")
d$Age <- d$age
d$`Country of residence` <- d$country_income_group %>% recode("Lower middle income" = "Low or middle income", "Upper middle income" = "Low or middle income", "Low income" = "Low or middle income")
d$`MOOC iteration` <- d$mooc

#reorder as necessary
d$Occupation <- factor(d$Occupation, levels = c("Health occupation", "Non-health occupation"))
d$Education <- factor(d$Education, levels = c("Post-secondary education", "Lower level of education"))
d$`Country of residence` <- factor(d$`Country of residence`, levels = c("High income", "Low or middle income"))

t3 <- table1::table1(~Age + Gender + Occupation + Education + `Country of residence` | `MOOC iteration`, data = d,
      render.continuous = c("Mean (standard deviation)" = "Mean (SD)"),
```

```
        topclass="Rtable1-zebra")
t3


#Create a demographic table across different MOOC iterations - create a table that only has complete cases as the model only used complete cases
d <- d %>% dplyr::filter(complete.cases(d))
d$mooc <- as.factor(d$mooc)
d$Gender <- as.factor(d$gender)
d$Occupation <- as.factor(d$occ) %>%
  dplyr::recode("1" = "Health occupation",
        "0" = "Other occupation")
d$Education <- as.factor(d$edu) %>%
  dplyr::recode("1" = "Post-secondary education",
        "0" = "Lower level of education")
d$Age <- d$age


t3 <- table1::table1(~Age + Gender + Occupation + Education + gdp_country | mooc, data = d,
       render.continuous = c("Mean (standard deviation)" = "Mean (SD)"),
        topclass="Rtable1-zebra")
t3

```

Explore data - demographics of completion

```{r}
load("combined_first_enrolment")


#recode gender for table
combined_first_enrolment$gender <- na_if(combined_first_enrolment$gender, "other")
combined_first_enrolment$gender <- factor(combined_first_enrolment$gender)
```

```
#Combine relevent data
d <- combined_first_enrolment %>%
  dplyr::select(user_id, age, gender, post_secondary_edu, anzsic_div_q_occ, mooc, completed,
country_income_group, gdp_country) %>%
  dplyr::mutate(edu = post_secondary_edu) %>%
  dplyr::mutate(mooc = as.factor(mooc),
                gender = gender,
                edu = post_secondary_edu,
                occ = as.character(anzsic_div_q_occ),
                completed = as.factor(completed)) %>%
  dplyr::select(user_id, age, gender, edu, occ, mooc, completed, gdp_country,
country_income_group)

d$age <- as.numeric(d$age)

#Create a demographic table comparing completions and non-completions
d$completed <- as.factor(d$completed) %>%
  dplyr::recode("1" = "Completed", "0" = "Not completed")
d$mooc <- as.factor(d$mooc)
d$Gender <- as.factor(d$gender) %>%
  recode("female" = "Female",
         "male" = "Male")
d$Occupation <- as.factor(d$occ) %>%
  dplyr::recode("1" = "Health occupation",
         "0" = "Non-health occupation")
d$Education <- as.factor(d$edu) %>%
  dplyr::recode("1" = "Post-secondary education",
         "0" = "Lower level of education")
d$Age <- d$age
```

```
d$`Country of residence` <- d$country_income_group %>% recode("Lower middle income" = "Low or middle income", "Upper middle income" = "Low or middle income", "Low income" = "Low or middle income")

d$`MOOC iteration` <- d$mooc


#reorder as necessary

d$Occupation <- factor(d$Occupation, levels = c("Health occupation", "Non-health occupation"))

d$Education <- factor(d$Education, levels = c("Post-secondary education", "Lower level of education"))

d$`Country of residence` <- factor(d$`Country of residence`, levels = c("High income", "Low or middle income"))


t3 <- table1::table1(~Age + Gender + Occupation + Education + `Country of residence` + `MOOC iteration` | completed, data = d,

      render.continuous = c("Mean (standard deviation)" = "Mean (SD)"),

       topclass="Rtable1-zebra")

t3


#Create a demographic table across different MOOC iterations - create a table that only has complete cases as the model only used complete cases

d <- d %>% dplyr::filter(complete.cases(d))

d$mooc <- as.factor(d$mooc)

d$Gender <- as.factor(d$gender)

d$Occupation <- as.factor(d$occ) %>%

  dplyr::recode("1" = "Health occupation",

        "0" = "Other occupation")

d$Education <- as.factor(d$edu) %>%

  dplyr::recode("1" = "Post-secondary education",

        "0" = "Lower level of education")

d$Age <- d$age


t3 <- table1::table1(~Age + Gender + Occupation + Education + gdp_country + mooc | completed, data = d,
```

````
    render.continuous = c("Mean (standard deviation)" = "Mean (SD)"),
     topclass="Rtable1-zebra")
t3
```
````

Compare demographics across years using simple individual variable glms and adjusted gams

````
```{r}
load("combined_first_enrolment")
windowsFonts("Arial" = windowsFont("Arial"))

#recode variables for analysis
combined_first_enrolment$gender <- combined_first_enrolment$gender %>% recode("male" = "0", "female" = "1")
combined_first_enrolment$gender <- na_if(combined_first_enrolment$gender, "other")
combined_first_enrolment$gender <- factor(combined_first_enrolment$gender, levels = c("0", "1"))
combined_first_enrolment$post_secondary_edu <- factor(combined_first_enrolment$post_secondary_edu, levels = c("0", "1"))
combined_first_enrolment$country_income_group <- combined_first_enrolment$country_income_group %>% dplyr::recode("High income" = "1", "Lower middle income" = "0", "Upper middle income" = "0", "Low income" = "0")
combined_first_enrolment$anzsic_div_q_occ <- factor(combined_first_enrolment$anzsic_div_q_occ, levels = c("0", "1"))

#model occupation profile across moocs
d2 <- combined_first_enrolment

glm1 <- glm(anzsic_div_q_occ ~ mooc, family = binomial(),
        data = d2)
summary(glm1)
````

```
d_stim <- data.frame(mooc = (sample(c("2016_07", "2017_03", "2018_05", "2018_10", "2019_05",
"2019_10", "2020_05"), 7, replace = F)))

#to obtain the correct CIs I have predicted using type = "link", giving mean and se on log scales,
claculated CIs and then back-transformed

fit <- predict(glm1, d_stim, type = "link", se.fit = T)

d_stim$emmean <- exp(fit$fit)/(1+exp(fit$fit))

d_stim$lower.CL <- exp(fit$fit-1.96*fit$se.fit)/(1+exp(fit$fit-1.96*fit$se.fit))

d_stim$upper.CL <- exp(fit$fit+1.96*fit$se.fit)/(1+exp(fit$fit+1.96*fit$se.fit))

d_stim

d_stim_p <- d_stim

d_stim_p1 <- d_stim_p %>% dplyr::rename("p" = "emmean")


gg1 <- ggplot(d_stim_p1, aes(x = mooc, y = p)) +

  geom_point() +

  geom_errorbar(aes(ymin=lower.CL, ymax=upper.CL), width = 0.2) +

  labs(title = "", y = "Proportion health workers", x = "Subsecquent MOOC iterations") +

  theme(text=element_text(family="Arial", size = 12),

        axis.title.x=element_text(size = 9.6, colour = "grey30")) +

  theme(axis.text.x=element_blank())

gg1


#calculate odds ratio

or_glm(data = d2, model = glm1)


#does relationship remain significant if you adjust for other demographics which are potential
confounders?

gam1 <- gam(anzsic_div_q_occ ~ mooc + gender + post_secondary_edu + country_income_group +
s(age, bs = "ts"), family = binomial(),

            data = d2)

summary(gam1) #yes, still significant
```

```
#model gender profile (men vs women across moocs)

d2 <- combined_first_enrolment %>% dplyr::filter(!is.na(gender))


glm2 <- glm(gender ~ mooc, family = binomial(),
         data = d2)

summary(glm2)


d_stim <- data.frame(mooc = (sample(c("2016_07", "2017_03", "2018_05", "2018_10", "2019_05",
"2019_10", "2020_05"), 7, replace = F)))

#to obtain the correct CIs I have predicted using type = "link", giving mean and se on log scales,
claculated CIs and then back-transformed

fit <- predict(glm2, d_stim, type = "link", se.fit = T)

d_stim$emmean <- exp(fit$fit)/(1+exp(fit$fit))

d_stim$lower.CL <- exp(fit$fit-1.96*fit$se.fit)/(1+exp(fit$fit-1.96*fit$se.fit))

d_stim$upper.CL <- exp(fit$fit+1.96*fit$se.fit)/(1+exp(fit$fit+1.96*fit$se.fit))

d_stim

d_stim_p <- d_stim

d_stim_p2 <- d_stim_p %>% dplyr::rename("p" = "emmean")



gg2 <- ggplot(d_stim_p2, aes(x = mooc, y = p)) +

  geom_point() +

  geom_errorbar(aes(ymin=lower.CL, ymax=upper.CL), width = 0.2) +

  labs(title = "", y = "Proportion women", x = "Subsequent MOOC iterations") +

  theme(text=element_text(family="Arial", size = 12),

      axis.title.x=element_text(size = 9.6, colour = "grey30")) +

  theme(axis.text.x=element_blank())

gg2
```

```
#calculate odds ratio

or_glm(data = d2, model = glm2)


#does relationship remain significant if you adjust for other demographics which are potential confounders?

gam2 <- gam(gender ~ mooc + anzsic_div_q_occ + post_secondary_edu + country_income_group + s(age, bs = "ts"), family = binomial(),

          data = d2)

summary(gam2) #only 2017_03 remain significant, and only just! Will not survive Bonferroni.




#model education profile across mooc iterations

d2 <- combined_first_enrolment


glm3 <- glm(post_secondary_edu ~ mooc, family = binomial(),

          data = d2)

summary(glm3)

d_stim <- data.frame(mooc = (sample(c("2016_07", "2017_03", "2018_05", "2018_10", "2019_05", "2019_10", "2020_05"), 7, replace = F)))

#to obtain the correct CIs I have predicted using type = "link", giving mean and se on log scales, claculated CIs and then back-transformed

fit <- predict(glm3, d_stim, type = "link", se.fit = T)

d_stim$emmean <- exp(fit$fit)/(1+exp(fit$fit))

d_stim$lower.CL <- exp(fit$fit-1.96*fit$se.fit)/(1+exp(fit$fit-1.96*fit$se.fit))

d_stim$upper.CL <- exp(fit$fit+1.96*fit$se.fit)/(1+exp(fit$fit+1.96*fit$se.fit))

d_stim

d_stim_p <- d_stim

d_stim_p3 <- d_stim_p %>% dplyr::rename("p" = "emmean")



gg3 <- ggplot(d_stim_p3, aes(x = mooc, y = p)) +
```

```
  geom_point() +
  geom_errorbar(aes(ymin=lower.CL, ymax=upper.CL), width = 0.2) +
  labs(title = "", y = "Propotion post-secondary educated", x = "Subsequent MOOC iterations") +
  theme(text=element_text(family="Arial", size = 12),
        axis.title.x=element_text(size = 9.6, colour = "grey30")) +
  theme(axis.text.x=element_blank())
gg3


#calculate odds ratio
or_glm(data = d2, model = glm3)


#does relationship remain significant if you adjust for other demographics which are potential confounders?
gam3 <- gam(post_secondary_edu ~ mooc + anzsic_div_q_occ + gender + country_income_group + s(age, bs = "ts"), family = binomial(),
            data = d2)
summary(gam3)  #yes, still significant




#model country of origin profile across mooc iterations
d2 <- combined_first_enrolment


glm4 <- glm(country_income_group ~ mooc, family = binomial(),
            data = d2)
summary(glm4)
d_stim <- data.frame(mooc = (sample(c("2016_07", "2017_03", "2018_05", "2018_10", "2019_05", "2019_10", "2020_05"), 7, replace = F)))
#to obtain the correct CIs I have predicted using type = "link", giving mean and se on log scales, claculated CIs and then back-transformed
fit <- predict(glm4, d_stim, type = "link", se.fit = T)
d_stim$emmean <- exp(fit$fit)/(1+exp(fit$fit))
```

```
d_stim$lower.CL <- exp(fit$fit-1.96*fit$se.fit)/(1+exp(fit$fit-1.96*fit$se.fit))

d_stim$upper.CL <- exp(fit$fit+1.96*fit$se.fit)/(1+exp(fit$fit+1.96*fit$se.fit))

d_stim

d_stim_p <- d_stim

d_stim_p4 <- d_stim_p %>% dplyr::rename("p" = "emmean")


gg4 <- ggplot(d_stim_p4, aes(x = mooc, y = p)) +
  geom_point() +
  geom_errorbar(aes(ymin=lower.CL, ymax=upper.CL), width = 0.2) +
  labs(title = "", y = "Proportion of participants from high income countries", x = "Subsequent MOOC iterations") +
  theme(axis.text.x=element_blank()) +
  theme(text=element_text(family="Arial", size = 12),
        axis.title.x=element_text(size = 9.6, colour = "grey30"))
gg4


#calculate odds ratio

or_glm(data = d2, model = glm4)


#does relationship remain significant if you adjust for other demographics which are potential confounders?

gam4 <- gam(country_income_group ~ mooc + anzsic_div_q_occ + gender + post_secondary_edu + s(age, bs = "ts"), family = binomial(),
            data = d2)

summary(gam4)  #2019_05 onwards are significant, others aren't



#model age profile across MOOC iterations

d2 <- combined_first_enrolment %>% dplyr::filter(!is.na(age))


#see how ages changed in the 25th, 50th and 75th quantile

qr1 <- rq(age ~ mooc, data = d2, tau = c(0.25, 0.50, 0.75))
```

```
summary(qr1)



#graph the mean and se age for each quartile

recover_data.rq <- emmeans:::recover_data.lm


emm_basis.rq <- function(object, trms, xlev, grid, se = NULL, ...){
  m <- model.frame(trms, grid, na.action = na.pass, xlev = xlev)
  X <- model.matrix(trms, m, contrasts.arg = object$contrasts)
  bhat <- object$coefficients
  Xmat <- model.matrix(trms, data = object$model)
  V = summary(object, se = se, covariance = TRUE)$cov
  nbasis = matrix(NA)
  dfargs = list(df = nrow(Xmat) - ncol(Xmat))
  dffun = function(k, dfargs) dfargs$df
  list(X = X,
       bhat = bhat,
       nbasis = nbasis,
       V = V,
       dffun = dffun,
       dfargs = dfargs)
}


qr25 <- rq(age ~ mooc, data = d2, tau = c(0.25))
em1 <- emmeans(qr25, ~ mooc)
errors1 <- confint(em1, level = 0.95, type = "response")
errors1
plot(em1, horizontal = F) +
  labs(title = "Age of participants in the lower (25th) quantile", x = "MOOC iteration", y = "Estimated quantile")
```

```
qr50 <- rq(age ~ mooc, data = d2, tau = c(0.50))

em2 <- emmeans(qr50, ~ mooc)

errors2 <- confint(em2, level = 0.95, type = "response")

errors2

plot(em2, horizontal = F) +

  labs(title = "Age of participants in the middle (50th) quantile", x = "MOOC iteration", y = "Estimated quantile")


qr75 <- rq(age ~ mooc, data = d2, tau = c(0.75))

em3 <- emmeans(qr75, ~ mooc)

errors3 <- confint(em3, level = 0.95, type = "response")

errors3

plot(em3, horizontal = F) +

  labs(title = "Age of participants in the upper (75th) quantile", x = "MOOC iteration", y = "Estimated quantile")


#create a dataframe with the point estimates of the quantiles

a <- errors1 %>% dplyr::select(mooc, emmean, lower.CL, upper.CL)  %>% dplyr::mutate(quant = "25") %>% dplyr::rename(age = emmean)

b <- errors2 %>% dplyr::select(mooc, emmean, lower.CL, upper.CL) %>% dplyr::mutate(quant = "50") %>% dplyr::rename(age = emmean)

c <- errors3 %>% dplyr::select(mooc, emmean, lower.CL, upper.CL) %>% dplyr::mutate(quant = "75") %>% dplyr::rename(age = emmean)

quantiles <- bind_rows(a,b,c)


e <- d2 %>% dplyr::group_by(mooc, age) %>% tally()


g1 <- left_join(e, quantiles)

g1a <- sample_n(g1, 20)

g2 <- g1 %>% dplyr::filter(!is.na(quant))


graph_for_paper <- ggplot(g1, aes(x = age, y = n, colour = mooc)) +

  geom_smooth(method = "gam", formula = y ~ s(x, bs = "ts"), size = 0.4) +
```

```
  geom_vline(g2, mapping = aes(xintercept = jitter(age, 0.5), linetype = quant, colour = mooc), alpha = 0.8, size = 0.4) +
  theme(text=element_text(family="Arial", size = 12)) +
  labs(title = "") +
  xlim(18,100) +
  ylab("Number of enrolments") +
  xlab("Age") +
  labs(colour = "MOOC iteration\n(year_month)", linetype = "Quantile")

addSmallLegend <- function(myPlot, pointSize = 0.5, spaceLegend = 0.8) {
    myPlot +
        guides(shape = guide_legend(override.aes = list(size = pointSize)),
               color = guide_legend(override.aes = list(size = pointSize))) +
        theme(legend.key.size = ggplot2::unit(spaceLegend, "lines"))
}

gg5 <- addSmallLegend(graph_for_paper)

ggsave(file = "Fig1.tiff", plot = gg5, width = 6, height = 2.7, dpi = 300, compression = 'lzw')

#does relationship remain significant if you adjust for other demographics which are potential confounders?
qr1 <- rq(age ~ mooc + anzsic_div_q_occ + gender + post_secondary_edu + country_income_group, data = d2, tau = c(0.25, 0.50, 0.75))
summary(qr1)
#lower and middle quartile significantly lower in 2019_10 and 2020_05. A few others only just significant and would not survive Bonferroni

####Create combined plot for catagorical variables
gg5 <- ggarrange(gg2, gg3, gg1, gg4,
```

```
        labels = c("A", "B", "C", "D"),
        font.label = list(size = 12, family = "Ariel"),
        ncol = 2, nrow = 2)
gg5
ggsave(gg5, file = "Fig2.tiff",  width=7.5, height=8.75, dpi=300, compression = 'lzw')
```

Similarly, model all demographics against completion

```{r}
load("combined_first_enrolment")

#recode variables for analysis
combined_first_enrolment$gender <- combined_first_enrolment$gender %>% recode("male" = "0", "female" = "1")
combined_first_enrolment$gender <- na_if(combined_first_enrolment$gender, "other")
combined_first_enrolment$country_income_group <- combined_first_enrolment$country_income_group %>% recode("High income" = "1", "Lower middle income" = "0", "Upper middle income" = "0", "Low income" = "0")
combined_first_enrolment$anzsic_div_q_occ <- as.factor(combined_first_enrolment$anzsic_div_q_occ)
combined_first_enrolment$anzsic_div_q_occ <- factor(combined_first_enrolment$anzsic_div_q_occ, levels = c("0", "1"))
combined_first_enrolment$post_secondary_edu <- factor(combined_first_enrolment$post_secondary_edu, levels = c("0", "1"))

#model all demographic variables and completion
d2 <- combined_first_enrolment
d2$mooc <- as.factor(d2$mooc)

qqnorm(d2$age)
qqline(d2$age)
```

```
#age residuals are not normally distributed so smoothed term needed



#see if fitting a thin plate regression spline does a reasonable job of modeling age
gam_age <- gam(completed ~ s(age, bs = "tp"), d2, family = binomial())
p <- data.frame(age = seq(18, 98, by = 5))
fit <- predict(gam_age, p, type = 'response')
p <- cbind(p, fit)
ggplot(p, aes(x = age, y = fit)) +
  geom_line()

n <- combined_first_enrolment %>% dplyr::filter(!is.na(age)) %>%  dplyr::group_by(age) %>% count()
n <- n %>% dplyr::rename("nage" = "n")
d <- combined_first_enrolment %>% dplyr::filter(!is.na(age)) %>% dplyr::group_by(completed,
age) %>% count()
d <- left_join(d, n)
d <- d %>% dplyr::mutate(perc = n/nage)
ggplot(d, aes(x = age, y = perc)) +
  geom_point() +
  facet_wrap(d$completed) +
  geom_smooth() +
  xlim(18, 89) #only a few people of each age over 90, so including these distorts trends in graph

#bs = "tp" seems to do a reasonable job of fitting age data

#use glms to mdoel each variable seperately
#gender
glm1 <- glm(completed ~ gender, family = binomial(),
        data = d2)
summary(glm1) #not associated
```

```
d_stim <- data.frame(gender = factor(sample(c(0,1), 2, replace = F)))

#to obtain the correct CIs I have predicted using type = "link", giving mean and se on log scales, claculated CIs and then back-transformed

fit <- predict(glm1, d_stim, type = "link", se.fit = T)

d_stim$emmean <- exp(fit$fit)/(1+exp(fit$fit))

d_stim$lower.CL <- exp(fit$fit-1.96*fit$se.fit)/(1+exp(fit$fit-1.96*fit$se.fit))

d_stim$upper.CL <- exp(fit$fit+1.96*fit$se.fit)/(1+exp(fit$fit+1.96*fit$se.fit))

d_stim

d_stim_p <- d_stim

d_stim_p <- d_stim_p %>% dplyr::rename("p" = "emmean")


gg1 <- ggplot(d_stim_p, aes(x = gender, y = p)) +
  geom_point() +
  geom_errorbar(aes(ymin=lower.CL, ymax=upper.CL), width = 0.2) +
  labs(title = "", y = "Proportion completed")  +
  scale_x_discrete(labels = c("Male\n ", "Female\n ")) +
  scale_y_continuous(breaks = c(0.60, 0.62, 0.64, 0.66, 0.68, 0.70), limits = c(0.6, 0.7)) +
  theme(axis.title.x=element_blank()) +
  theme(text = element_text(size = 12, family = "Ariel"))
gg1


#calculate odds ratios

or_glm(data = d2, model = glm1)


#education

glm2 <- glm(completed ~ post_secondary_edu, family = binomial(),
      data = d2)

summary(glm2) #highly associated


d_stim <- data.frame(post_secondary_edu = factor(sample(c(0,1), 2, replace = F)))

#to obtain the correct CIs I have predicted using type = "link", giving mean and se on log scales, claculated CIs and then back-transformed
```

```
fit <- predict(glm2, d_stim, type = "link", se.fit = T)
d_stim$emmean <- exp(fit$fit)/(1+exp(fit$fit))
d_stim$lower.CL <- exp(fit$fit-1.96*fit$se.fit)/(1+exp(fit$fit-1.96*fit$se.fit))
d_stim$upper.CL <- exp(fit$fit+1.96*fit$se.fit)/(1+exp(fit$fit+1.96*fit$se.fit))
d_stim
d_stim_p <- d_stim
d_stim_p <- d_stim_p %>% dplyr::rename("p" = "emmean")

gg2 <- ggplot(d_stim_p, aes(x = post_secondary_edu, y = p)) +
  geom_point() +
  geom_errorbar(aes(ymin=lower.CL, ymax=upper.CL), width = 0.2) +
  labs(title = "") +
  scale_x_discrete(labels = c("Secondary education\n or less", "Post-secondary\n education")) +
  scale_y_continuous(breaks = c(0.60, 0.62, 0.64, 0.66, 0.68, 0.70), limits = c(0.6, 0.7)) +
  theme(axis.title.x=element_blank(),
        axis.title.y=element_blank()) +
  theme(text = element_text(size = 12, family = "Ariel"))
gg2

#calculate odds ratios
or_glm(data = d2, model = glm2)



#occupation
glm3 <- glm(completed ~ anzsic_div_q_occ, family = binomial(),
            data = d2)
summary(glm3) #associated

d_stim <- data.frame(anzsic_div_q_occ = factor(sample(c(0,1), 2, replace = F)))
#to obtain the correct CIs I have predicted using type = "link", giving mean and se on log scales, claculated CIs and then back-transformed
```

```
fit <- predict(glm3, d_stim, type = "link", se.fit = T)

d_stim$emmean <- exp(fit$fit)/(1+exp(fit$fit))

d_stim$lower.CL <- exp(fit$fit-1.96*fit$se.fit)/(1+exp(fit$fit-1.96*fit$se.fit))

d_stim$upper.CL <- exp(fit$fit+1.96*fit$se.fit)/(1+exp(fit$fit+1.96*fit$se.fit))

d_stim

d_stim_p <- d_stim

d_stim_p <- d_stim_p %>% dplyr::rename("p" = "emmean")


gg3 <- ggplot(d_stim_p, aes(x = anzsic_div_q_occ, y = p)) +
  geom_point() +
  geom_errorbar(aes(ymin=lower.CL, ymax=upper.CL), width = 0.2) +
  labs(title = "", x = "Participant occupation", y = "Proportion completed") +
  scale_x_discrete(labels = c("Non-health occupation\n ", "Health occupation\n ")) +
  scale_y_continuous(breaks = c(0.60, 0.62, 0.64, 0.66, 0.68, 0.70), limits = c(0.6, 0.7)) +
  theme(axis.title.x=element_blank()) +
  theme(text = element_text(size = 12, family = "Ariel"))
gg3


#calculate odds ratios
or_glm(data = d2, model = glm3)





#country
glm4 <- glm(completed ~ country_income_group, family = binomial(),
            data = d2)
summary(glm4) #highly associated


d_stim <- data.frame(country_income_group = factor(sample(c(0,1), 2, replace = F)))
#to obtain the correct CIs I have predicted using type = "link", giving mean and se on log scales, claculated CIs and then back-transformed
```

```
fit <- predict(glm4, d_stim, type = "link", se.fit = T)

d_stim$emmean <- exp(fit$fit)/(1+exp(fit$fit))

d_stim$lower.CL <- exp(fit$fit-1.96*fit$se.fit)/(1+exp(fit$fit-1.96*fit$se.fit))

d_stim$upper.CL <- exp(fit$fit+1.96*fit$se.fit)/(1+exp(fit$fit+1.96*fit$se.fit))

d_stim

d_stim_p <- d_stim

d_stim_p <- d_stim_p %>% dplyr::rename("p" = "emmean")


gg4 <- ggplot(d_stim_p, aes(x = country_income_group, y = p)) +
  geom_point() +
  geom_errorbar(aes(ymin=lower.CL, ymax=upper.CL), width = 0.2) +
  labs(title = "", x = "Participant country of residence", y = "Proportion completed") +
  scale_x_discrete(labels = c("Low or middle\n income country", "High\n income country")) +
  scale_y_continuous(breaks = c(0.60, 0.62, 0.64, 0.66, 0.68, 0.70), limits = c(0.6, 0.7)) +
  theme(axis.title.x=element_blank(),
        axis.title.y=element_blank()) +
  theme(text = element_text(size = 12, family = "Ariel"))
gg4


#calculate odds ratios
or_glm(data = d2, model = glm4)




#mooc iteration of enrolment
glm5 <- glm(completed ~ mooc, family = binomial(),
            data = d2)
summary(glm5) #highly associated


d_stim <- data.frame(mooc = (sample(c("2016_07", "2017_03", "2018_05", "2018_10", "2019_05",
"2019_10", "2020_05"), 7, replace = F)))
#to obtain the correct CIs I have predicted using type = "link", giving mean and se on log scales,
claculated CIs and then back-transformed
```

```
fit <- predict(glm5, d_stim, type = "link", se.fit = T)

d_stim$emmean <- exp(fit$fit)/(1+exp(fit$fit))

d_stim$lower.CL <- exp(fit$fit-1.96*fit$se.fit)/(1+exp(fit$fit-1.96*fit$se.fit))

d_stim$upper.CL <- exp(fit$fit+1.96*fit$se.fit)/(1+exp(fit$fit+1.96*fit$se.fit))

d_stim

d_stim_p <- d_stim

d_stim_p <- d_stim_p %>% dplyr::rename("p" = "emmean")


ggplot(d_stim_p, aes(x = mooc, y = p)) +

  geom_point() +

  geom_errorbar(aes(ymin=lower.CL, ymax=upper.CL), width = 0.2) +

  labs(title = "MOOC completion by MOOC iteration of enrolment", x = "MOOC iteration", y =
"Proportion completed")


#calculate odds ratios

or_glm(data = d2, model = glm5)




#age

gam1b <- gam(factor(completed) ~ s(age, bs = "tp"), d2, family = binomial())

b0 <- expand.grid(age = seq(18, 100, length = 20))

fit <- predict(gam1b, b0, se.fit = TRUE)


b0$emmean <- exp(fit$fit)/(1+exp(fit$fit))

b0$lower.CL <- exp(fit$fit-1.96*fit$se.fit)/(1+exp(fit$fit-1.96*fit$se.fit))

b0$upper.CL <- exp(fit$fit+1.96*fit$se.fit)/(1+exp(fit$fit+1.96*fit$se.fit))

b0


age <- ggplot(b0, aes(x = age, y = emmean)) +

  geom_ribbon(aes(ymin = lower.CL, ymax = upper.CL), alpha = .1, colour = NA) +
```

```
  geom_line() +
  labs(x = "Participant age", y = "Proportion complete") +
  theme(text = element_text(size = 12, family = "Ariel")) +
  theme(legend.key.size = unit(0.3, 'cm'))
age

ggsave(age, file = "Fig4.tiff", width = 6, height = 2.7, dpi=300, compression = 'lzw')

#From inspection of the graph, trends change at aproximately age 35, 61, 74 and 91, so calculate OR at each of these points
or_gam(data = d2, model = gam1b, pred = "age", values = c(18,35))
or_gam(data = d2, model = gam1b, pred = "age", values = c(35,61))
or_gam(data = d2, model = gam1b, pred = "age", values = c(61,74))
or_gam(data = d2, model = gam1b, pred = "age", values = c(74, 91))
or_gam(data = d2, model = gam1b, pred = "age", values = c(91, 100))

#calculate odds-ratios at 25 yeah cut-points
or_gam(data = d2, model = gam1b, pred = "age", values = c(25,50))
or_gam(data = d2, model = gam1b, pred = "age", values = c(50,70))
or_gam(data = d2, model = gam1b, pred = "age", values = c(70, 90))


#see what relationships remain significant when you adjust for all confounders
gam1 <- gam(completed ~ s(age, bs = "tp") + mooc + country_income_group + anzsic_div_q_occ + post_secondary_edu + gender, d2, family = binomial())
summary(gam1) #all relationships remain significant!


####Create combined plot for categorical variables
gg5 <- ggarrange(gg1, gg2, gg3, gg4,
        labels = c("A", "B", "C", "D"),
        font.label = list(size = 12, family = "Ariel"),
```

```
    ncol = 2, nrow = 2)

ggsave(gg5, file = "Fig5.tiff",  width=7.5, height=8.75, dpi=300, compression = 'lzw')
```

Plot quiz progression

```{r}
load("combined_first_enrolment")

d <- combined_first_enrolment
d$Quiz_A <- d$quiz_1_percent
d$Quiz_B <- d$quiz_3_percent
d$Quiz_C <- d$quiz_4_percent

d <- d %>% dplyr::select(completed, Quiz_A, Quiz_B, Quiz_C, user_id, mooc)

d1 <- d %>% dplyr::filter(!is.na(Quiz_A))
d1 <- d1 %>% dplyr::mutate(Quiz_A_attempt = "1")
d <- left_join(d, d1)

d1 <- d %>% dplyr::filter(!is.na(Quiz_B))
d1 <- d1 %>% dplyr::mutate(Quiz_B_attempt = "1")
d <- left_join(d, d1)

d1 <- d %>% dplyr::filter(!is.na(Quiz_C))
d1 <- d1 %>% dplyr::mutate(Quiz_C_attempt = "1")
d <- left_join(d, d1)

#percentage of completers who attempt each quiz
d2 <- d %>% dplyr::filter(completed == "0")
```

```
a <- d2 %>% nrow()

b <- d2 %>% dplyr::filter(is.na(Quiz_A_attempt)) %>% nrow()

c <- d2 %>% dplyr::filter(is.na(Quiz_B_attempt)) %>% nrow()

e <- d2 %>% dplyr::filter(is.na(Quiz_C_attempt)) %>% nrow()


b/a #66.1% of the people who don't complete the MOOC don't attempt Quiz A

c/a #93.5% of the people who don't complete the MOOC don't attempt Quiz B

e/a #99.7% of the people who don't complete the MOOC don't attempt Quiz C


#graph this!

a <- d %>% dplyr::group_by(mooc) %>% count() %>% dplyr::rename(n_enrol = n)

b <- d %>% dplyr::filter(Quiz_A_attempt == "1") %>% dplyr::group_by(mooc) %>% count() %>%
dplyr::rename(n_quiz_a = n)

c <- d %>% dplyr::filter(Quiz_B_attempt == "1") %>% dplyr::group_by(mooc) %>% count() %>%
dplyr::rename(n_quiz_b = n)

e <- d %>% dplyr::filter(Quiz_C_attempt == "1") %>% dplyr::group_by(mooc) %>% count() %>%
dplyr::rename(n_quiz_C = n)

f <- d %>% dplyr::filter(completed == "1") %>% dplyr::group_by(mooc) %>% count() %>%
dplyr::rename(completed = n)


progression <- left_join(a,b)

progression <- left_join(progression, c)

progression <- left_join(progression, e)

progression <- left_join(progression, f)


progression_long <- pivot_longer(progression, -mooc, names_to = "mooc_point")


progression_long$mooc_point <- factor(progression_long$mooc_point, levels = c("n_enrol",
"n_quiz_a", "n_quiz_b", "n_quiz_C", "completed"))


progression <- ggplot(progression_long, aes(x = mooc_point, y = value, colour = mooc, group =
mooc)) +
```

```
  geom_point() +
  geom_line() +
  labs(title = "Progression through the MOOC", x = "MOOC milestone", y = "Number of participants", colour = "MOOC iteration (year_month)") +
  scale_x_discrete(labels = c("Enrolment", "Quiz A", "Quiz B", "Quiz C", "Completion")) +
  theme(text=element_text(family="Arial")) +
  theme_set(theme_gray(base_size=10)) +
  theme(plot.title = element_text(size = 12)) +
  theme(axis.text.y = element_text(size = 10)) +
  theme(axis.text.x = element_text(size = 10)) +
  theme(legend.title = element_text(size = 10)) +
  theme(legend.text = element_text(size = 10))
progression

ggsave(progression, file = "Fig3.tiff", width = 1500, height = 600, units = "px", dpi=300, compression = 'lzw')

##try a different way of formetting for Plos
#Basic plot with key info
progression <- ggplot(progression_long, aes(x = mooc_point, y = value, colour = mooc, group = mooc)) +
  geom_point(size = 0.4) +
  geom_line(size = 0.4) +
  labs(x = "MOOC milestone", y = "Number of participants", colour = "MOOC iteration\n(year_month)") +
  scale_x_discrete(labels = c("Enrolment", "Quiz A", "Quiz B", "Quiz C", "Completion")) +
  theme(text = element_text(size = 12, family = "Ariel")) +
  theme(legend.key.size = unit(0.4, 'cm'))

ggsave(progression, file = "Fig3.tiff", width = 6, height = 2.7, dpi=300, compression = 'lzw')
```

```

Feedback on the MOOC - general survey summary

```{r}
load("combined_first_completion")

#count responses to outcome_understanding, strongly agree coded 1 and agree coded 2
a <-combined_first_completion %>%
  dplyr::filter(outcome_understanding == "1" | outcome_understanding == "2") %>% nrow()
b <- combined_first_completion %>%
  dplyr::filter(!is.na(outcome_understanding)) %>% nrow()
c <- a/b*100
c
#98% of the 16626 people who completed this question on the feedback survey agreed or strongly agreed that "My understanding of dementia has improved".

#count responses to outcome_info_risk_reduction
a1 <-combined_first_completion %>%
  dplyr::filter(outcome_info_risk_reduction == "1" | outcome_info_risk_reduction == "2") %>% nrow()
b1 <- combined_first_completion %>%
  dplyr::filter(!is.na(outcome_info_risk_reduction)) %>% nrow()
c1 <- a1/b1*100
#95% of the 16587 people who completed this question on the feedback survey agreed or strongly agreed that "The MOOC has given me the information I need to reduce my dementia risk.".

#count responses to outcome_behaviour_lifestyle
a2 <-combined_first_completion %>%
  dplyr::filter(outcome_behaviour_lifestyle == "1" | outcome_behaviour_lifestyle == "2") %>% nrow()
b2 <- combined_first_completion %>%
  dplyr::filter(!is.na(outcome_behaviour_lifestyle)) %>% nrow()

```
c2 <- a2/b2*100

#86% of the 16603 people who completed this question on the feedback survey agreed or strongly agreed that "The MOOC has had an impact on my behaviour and lifestyle choices.".

#count responses to outcome_already_applied

a3 <-combined_first_completion %>%

  dplyr::filter(outcome_already_applied == "1" | outcome_already_applied == "2") %>% nrow()

b3 <- combined_first_completion %>%

  dplyr::filter(!is.na(outcome_already_applied)) %>% nrow()

c3 <- a3/b3*100

#73% of the 16593 people who completed this question on the feedback survey agreed or strongly agreed that "I have already applied the knowledge I have gained from the MOOC.".

#count responses to outcome_recommend

a4 <-combined_first_completion %>%

  dplyr::filter(outcome_recommend == "1" | outcome_recommend == "2") %>% nrow()

b4 <- combined_first_completion %>%

  dplyr::filter(!is.na(outcome_recommend)) %>% nrow()

c4 <- a4/b4*100

#99% of the 16595 people who completed this question on the feedback survey agreed or strongly agreed that "I would recommend the MOOC to others.".

#count the number of complete responses to feedback survey

a5 <- combined_first_completion %>% dplyr::filter(feedback_completed == "1") %>% nrow()

a5

b5 <- combined_first_completion %>% dplyr::filter(completed == "1") %>% nrow()

c5 <- a5/b5*100

#57% of users who completed the MOOC attempted the feedback survey

#count the number of application Q text responses

a6 <- combined_first_completion %>% dplyr::filter(!is.na(outcome_already_applied_text)) %>% nrow
```

```
b6 <- combined_first_completion %>% dplyr::filter(feedback_completed == "1") %>% nrow()

c6 <- a6/b6*100

#41% of people who attempted the feedback survey provided a response to the text question about knowledge application: 8521 people provided a response


#count the number of impression_best text responses

a7 <- combined_first_completion %>% dplyr::filter(!is.na(impression_best)) %>% nrow

b7 <- combined_first_completion %>% dplyr::filter(feedback_completed == "1") %>% nrow()

c7 <- a7/b7*100

#59% of people who attempted the feedback survey provided a response to the text question about best features of the MOOC


#count the number of impression_worst text responses

a8 <- combined_first_completion %>% dplyr::filter(!is.na(impression_worst)) %>% nrow

b8 <- combined_first_completion %>% dplyr::filter(feedback_completed == "1") %>% nrow()

c8 <- a8/b8*100

#51% of people who attempted the feedback survey provided a response to the text question about worst features of the MOOC



#tabulate these results

statement <- c("My understanding of dementia has improved", "The MOOC has given me the information I need to reduce my dementia risk", "The MOOC has had an impact on my behaviour and lifestyle choices", "I have already applied the knowledge I have gained from the MOOC", "I would recommend the MOOC to others")

percentage <- c(c,c1,c2,c3,c4)

responses <- c(a,a1,a2,a3,a4)


tabulate <- data.frame(statement, percentage, responses)


tabulate$`Feedback survey statement` <- tabulate$statement

tabulate$`Percentage "agree" or "strongly agree"` <- tabulate$percentage

tabulate$`Total number of responses` <- tabulate$responses
```

tabulate <- tabulate %>% dplyr::select(`Feedback survey statement`, `Percentage "agree" or "strongly agree"`, `Total number of responses`)

tabulate

write.csv(tabulate, file = "Feedback survey response table.csv")

```

###HERE!!!!!!!!!!!!!

From peer review: which demographics are associted with MOOC outcomes

```{r}

load("combined_first_completion")

#recode variables for analysis

combined_first_completion$outcome_understanding <- combined_first_completion$outcome_understanding %>% recode("1" = "1", "2" = "1", "3" = "0", "4" = "0", "5" = "0") %>% as.factor()

combined_first_completion$outcome_info_risk_reduction <- combined_first_completion$outcome_info_risk_reduction %>% recode("1" = "1", "2" = "1", "3" = "0", "4" = "0", "5" = "0") %>% as.factor()

combined_first_completion$outcome_behaviour_lifestyle <- combined_first_completion$outcome_behaviour_lifestyle %>% recode("1" = "1", "2" = "1", "3" = "0", "4" = "0", "5" = "0") %>% as.factor()

combined_first_completion$outcome_already_applied <- combined_first_completion$outcome_already_applied %>% recode("1" = "1", "2" = "1", "3" = "0", "4" = "0", "5" = "0") %>% as.factor()

combined_first_completion$outcome_recommend <- combined_first_completion$outcome_recommend %>% recode("1" = "1", "2" = "1", "3" = "0", "4" = "0", "5" = "0") %>% as.factor()

combined_first_completion$learning_satisfied <- combined_first_completion$learning_satisfied %>% recode("1" = "1", "2" = "1", "3" = "0", "4" = "0", "5" = "0") %>% as.factor()

combined_first_completion$gender <- combined_first_completion$gender %>% recode("male" = "1", "female" = "0")

```
combined_first_completion$gender <- na_if(combined_first_completion$gender, "other")

combined_first_completion$gender <- factor(combined_first_completion$gender, levels = c("0", "1"))

combined_first_completion$country_income_group <- combined_first_completion$country_income_group %>% recode("High income" = "1", "Lower middle income" = "0", "Upper middle income" = "0", "Low income" = "0")

combined_first_completion$anzsic_div_q_occ <- as.factor(combined_first_completion$anzsic_div_q_occ)

combined_first_completion$anzsic_div_q_occ <- factor(combined_first_completion$anzsic_div_q_occ, levels = c("0", "1"))

combined_first_completion$post_secondary_edu <- factor(combined_first_completion$post_secondary_edu, levels = c("0", "1"))

combined_first_completion$gender <- factor(combined_first_completion$gender, levels = c("0", "1"))

combined_first_completion$country_income_group <- factor(combined_first_completion$country_income_group, levels = c("0", "1"))

d2 <- combined_first_completion

qqnorm(d2$age)

qqline(d2$age)

#age residuals are not normally distributed so smoothed term needed

#see if fitting a thin plate regression spline does a reasonable job of modeling age

gam_age <- gam(outcome_understanding ~ s(age, bs = "tp"), d2, family = binomial())

p <- data.frame(age = seq(18, 98, by = 5))

fit <- predict(gam_age, p, type = 'response')

p <- cbind(p, fit)

ggplot(p, aes(x = age, y = fit)) +

  geom_line()
```

n <- d2 %>% dplyr::filter(!is.na(age), !is.na(outcome_understanding)) %>% dplyr::group_by(age) %>% count()

n <- n %>% dplyr::rename("nage" = "n")

d <- d2 %>% dplyr::filter(!is.na(age), !is.na(outcome_understanding)) %>% dplyr::group_by(outcome_understanding, age) %>% count()

d <- left_join(d, n)

d <- d %>% dplyr::mutate(perc = n/nage)

ggplot(d, aes(x = age, y = perc)) +

 geom_point() +

 facet_wrap(d$outcome_understanding) +

 geom_smooth() +

 xlim(18, 89) #only a few people of each age over 90, so including these distorts trends in graph

#bs = "tp" seems to do a reasonable job of fitting age data

#start by seeing if anything is significant in fully adjusted models

gam1 <- gam(outcome_understanding ~ s(age, bs = "tp") + country_income_group + anzsic_div_q_occ + post_secondary_edu + gender, d2, family = binomial())

summary(gam1) #age significant: older people more likley to agree "My understanding of dementia prevention has improved"

gam2 <- gam(outcome_info_risk_reduction ~ s(age, bs = "tp") + country_income_group + anzsic_div_q_occ + post_secondary_edu + gender, d2, family = binomial())

summary(gam2) #age, gender and country significnat: older poeple and women more likley to agree that "The MOOC has given me the information I need to reduce my dementia risk", people from high income countries are less likley

gam3 <- gam(outcome_behaviour_lifestyle ~ s(age, bs = "tp") + country_income_group + anzsic_div_q_occ + post_secondary_edu + gender, d2, family = binomial())

summary(gam3) #age, gender, education, occupation and country significant: older poeple, women and health workers more likley to agree that "The MOOC has had an impact on my behaviour and lifestyle choices", people with post-secondary education and people from high income countries are less likley

gam4 <- gam(outcome_already_applied ~ s(age, bs = "tp") + country_income_group + anzsic_div_q_occ + post_secondary_edu + gender, d2, family = binomial())

summary(gam4) #age, education, occupation and country significant: older poeple, people with post-secondary education and health workers more likley to agree that "I have already applied the knowledge I have gained from the MOOC", people from high income countries are less likley

gam5 <- gam(outcome_recommend ~ s(age, bs = "tp") + country_income_group + anzsic_div_q_occ + post_secondary_edu + gender, d2, family = binomial())

summary(gam5) #age and country significant: older poeple and people from high income countries are more likley to agree that "I would recommend the MOOC to others"

gam6 <- gam(learning_satisfied ~ s(age, bs = "tp") + country_income_group + anzsic_div_q_occ + post_secondary_edu + gender, d2, family = binomial())

summary(gam6) #occupation and gender significant: women more likley to agree that "I was satisfied with my MOOC learning experience", health workers less likley

#set up repeatable code to model demographics and outcomes

individual_demographic_models <- function(d2, outcome, outcome_title) {

#use glms to mdoel each variable seperately

#gender

d2$dem <- d2$gender

glm1 <- glm(outcome ~ dem, family = binomial(),

        data = d2)

summary(glm1) #not associated

model <- summary(glm1)

pa <- model$coefficients[,4] %>% as.data.frame()

p <- pa[2,1]

#look at predicted plots

```
d_stim <- data.frame(dem = factor(sample(c(0,1), 2, replace = F)))

d_stim <- d_stim[order(d_stim$dem),] %>% as.data.frame()

colnames(d_stim) <- "dem"

#to obtain the correct CIs I have predicted using type = "link", giving mean and se on log scales, claculated CIs and then back-transformed

fit <- predict(glm1, d_stim, type = "link", se.fit = T)

d_stim$emmean <- exp(fit$fit)/(1+exp(fit$fit))

d_stim$lower.CL <- exp(fit$fit-1.96*fit$se.fit)/(1+exp(fit$fit-1.96*fit$se.fit))

d_stim$upper.CL <- exp(fit$fit+1.96*fit$se.fit)/(1+exp(fit$fit+1.96*fit$se.fit))

d_stim

d_stim_p <- d_stim

d_stim_p <- d_stim_p %>% dplyr::rename("p" = "emmean")


gg1 <- ggplot(d_stim_p, aes(x = dem, y = p)) +

  geom_point() +

  geom_errorbar(aes(ymin=lower.CL, ymax=upper.CL), width = 0.2) +

  labs(title = "MOOC outcome by participant gender (model predictions)", y = "Proportion agreed")  +

  scale_x_discrete(labels = c("Female", "Male")) +

  theme(axis.title.x=element_blank())

gg1


#compare to raw data

t1 <- table(d2$outcome, d2$dem)

#proportion who agree

a <- t1[2,1]/(t1[1,1] + t1[2,1]) #women

b <- t1[2,2]/(t1[1,2] + t1[2,2]) #men

d3 <- data.frame(variable = c("dem0", "dem1"), value = c(a,b))


gg2 <- ggplot(d3, aes(x = variable, y = value)) +

  geom_point() +

  labs(title = "MOOC outcome by participant gender (actual)", y = "Proportion agreed")  +
```

```
  scale_x_discrete(labels = c("Female", "Male")) +
  theme(axis.title.x=element_blank())
gg2

#calculate odds ratios
or <- or_glm(data = d2, model = glm1)

#prepare OR and CI for results table
or$oddsratio <- or$oddsratio %>% round(2)
or$`ci_low (2.5)` <- or$`ci_low (2.5)` %>% round(2)
or$`ci_high (97.5)` <- or$`ci_high (97.5)` %>% round(2)
or$results <- paste0(or$oddsratio, " (", or$`ci_low (2.5)`, " - ", or$`ci_high (97.5)`, ")")

#prepare predicted proportions for table
d_stim_p$p <- d_stim_p$p %>%  round(2)
d_stim_p$lower.CL <- d_stim_p$lower.CL %>%  round(2)
d_stim_p$upper.CL <- d_stim_p$upper.CL %>%  round(2)
d_stim_p$results <- paste0(d_stim_p$p, " (", d_stim_p$lower.CL, " - ", d_stim_p$upper.CL, ")")



#build results table section
results_gender <- data.frame(Gender = c("Male", "Female", "Missing"),
                       Agreed = c(nrow(d2 %>% filter(outcome == 1 & dem == 1)),
                                  nrow(d2 %>% filter(outcome == 1 & dem == 0)),
                                  nrow(d2 %>% filter(outcome == 1 & is.na(gender)))),
                       Not_agreed = c(nrow(d2 %>% filter(outcome == 0 & dem == 1)),
                                  nrow(d2 %>% filter(outcome == 0 & dem == 0)),
                                  nrow(d2 %>% filter(outcome == 0 & is.na(gender)))),
                       Proportion_agreed_confidence_interval = c(d_stim_p$results[2],
d_stim_p$results[1], ""),
```

```
                p_value = c(round(p, 5), "reference", ""),
                Odds_ratio_confidence_interval = c(or$results[1], "reference", "")
                )


#anzsic_div_q_occ
d2$dem <- d2$anzsic_div_q_occ
glm1 <- glm(outcome ~ dem, family = binomial(),
      data = d2)
summary(glm1) #not associated
model <- summary(glm1)
pa <- model$coefficients[,4] %>% as.data.frame()
p <- pa[2,1]

#look at predicted plots
d_stim <- data.frame(dem = factor(sample(c(0,1), 2, replace = F)))
d_stim <- d_stim[order(d_stim$dem),] %>% as.data.frame()
colnames(d_stim) <- "dem"
#to obtain the correct CIs I have predicted using type = "link", giving mean and se on log scales, claculated CIs and then back-transformed
fit <- predict(glm1, d_stim, type = "link", se.fit = T)
d_stim$emmean <- exp(fit$fit)/(1+exp(fit$fit))
d_stim$lower.CL <- exp(fit$fit-1.96*fit$se.fit)/(1+exp(fit$fit-1.96*fit$se.fit))
d_stim$upper.CL <- exp(fit$fit+1.96*fit$se.fit)/(1+exp(fit$fit+1.96*fit$se.fit))
d_stim
d_stim_p <- d_stim
d_stim_p <- d_stim_p %>% dplyr::rename("p" = "emmean")

gg1 <- ggplot(d_stim_p, aes(x = dem, y = p)) +
  geom_point() +
  geom_errorbar(aes(ymin=lower.CL, ymax=upper.CL), width = 0.2) +
```

```
  labs(title = "MOOC outcome by participant occupations (model predictions)", y = "Proportion agreed")  +
  scale_x_discrete(labels = c("Other", "Health")) +
  theme(axis.title.x=element_blank())
gg1

#compare to raw data
t1 <- table(d2$outcome, d2$dem)
#proportion who agree
a <- t1[2,1]/(t1[1,1] + t1[2,1])
b <- t1[2,2]/(t1[1,2] + t1[2,2])
d3 <- data.frame(variable = c("dem0", "dem1"), value = c(a,b))

gg2 <- ggplot(d3, aes(x = variable, y = value)) +
  geom_point() +
  labs(title = "MOOC outcome by participant occupations (actual)", y = "Proportion agreed")  +
  scale_x_discrete(labels = c("other", "Health")) +
  theme(axis.title.x=element_blank())
gg2

#calculate odds ratios
or <- or_glm(data = d2, model = glm1)

#prepare OR and CI for results table
or$oddsratio <- or$oddsratio %>% round(2)
or$`ci_low (2.5)` <- or$`ci_low (2.5)` %>% round(2)
or$`ci_high (97.5)` <- or$`ci_high (97.5)` %>% round(2)
or$results <- paste0(or$oddsratio, " (", or$`ci_low (2.5)`, " - ", or$`ci_high (97.5)`, ")")

#prepare predicted proportions for table
d_stim_p$p <- d_stim_p$p %>%  round(2)
```

```
d_stim_p$lower.CL <- d_stim_p$lower.CL %>%  round(2)

d_stim_p$upper.CL <- d_stim_p$upper.CL %>%  round(2)

d_stim_p$results <- paste0(d_stim_p$p, " (", d_stim_p$lower.CL, " - ", d_stim_p$upper.CL, ")")




#build results table section

results_occupation <- data.frame(Occupation = c("Health Occupation", "Non-health occupation", 
"Missing"),

                    Agreed = c(nrow(d2 %>% filter(outcome == 1 & dem == 1)),

                          nrow(d2 %>% filter(outcome == 1 & dem == 0)),

                          nrow(d2 %>% filter(outcome == 1 & is.na(dem)))),

                    Not_agreed = c(nrow(d2 %>% filter(outcome == 0 & dem == 1)),

                          nrow(d2 %>% filter(outcome == 0 & dem == 0)),

                          nrow(d2 %>% filter(outcome == 0 & is.na(dem)))),

                    Proportion_agreed_confidence_interval = c(d_stim_p$results[2], 
d_stim_p$results[1], ""),

                    p_value = c(round(p, 5), "reference", ""),

                    Odds_ratio_confidence_interval = c(or$results[1], "reference", "")

                    )



#Education

d2$dem <- d2$post_secondary_edu

glm1 <- glm(outcome ~ dem, family = binomial(),

        data = d2)

summary(glm1) #not associated

model <- summary(glm1)

pa <- model$coefficients[,4] %>% as.data.frame()

p <- pa[2,1]


#look at predicted plots
```

```
d_stim <- data.frame(dem = factor(sample(c(0,1), 2, replace = F)))

d_stim <- d_stim[order(d_stim$dem),] %>% as.data.frame()

colnames(d_stim) <- "dem"

#to obtain the correct CIs I have predicted using type = "link", giving mean and se on log scales, claculated CIs and then back-transformed

fit <- predict(glm1, d_stim, type = "link", se.fit = T)

d_stim$emmean <- exp(fit$fit)/(1+exp(fit$fit))

d_stim$lower.CL <- exp(fit$fit-1.96*fit$se.fit)/(1+exp(fit$fit-1.96*fit$se.fit))

d_stim$upper.CL <- exp(fit$fit+1.96*fit$se.fit)/(1+exp(fit$fit+1.96*fit$se.fit))

d_stim

d_stim_p <- d_stim

d_stim_p <- d_stim_p %>% dplyr::rename("p" = "emmean")


gg1 <- ggplot(d_stim_p, aes(x = dem, y = p)) +

  geom_point() +

  geom_errorbar(aes(ymin=lower.CL, ymax=upper.CL), width = 0.2) +

  labs(title = "MOOC outcome by participant education (model predictions)", y = "Proportion agreed")  +

  scale_x_discrete(labels = c("Lower edu", "Post-secondary edu")) +

  theme(axis.title.x=element_blank())

gg1


#compare to raw data

t1 <- table(d2$outcome, d2$dem)

#proportion who agree

a <- t1[2,1]/(t1[1,1] + t1[2,1])

b <- t1[2,2]/(t1[1,2] + t1[2,2])

d3 <- data.frame(variable = c("dem0", "dem1"), value = c(a,b))


gg2 <- ggplot(d3, aes(x = variable, y = value)) +

  geom_point() +

  labs(title = "MOOC outcome by participant education (actual)", y = "Proportion agreed")  +
```

```
  scale_x_discrete(labels = c("Lower edu", "Post-secondary edu")) +
  theme(axis.title.x=element_blank())
gg2

#calculate odds ratios
or <- or_glm(data = d2, model = glm1)

#prepare OR and CI for results table
or$oddsratio <- or$oddsratio %>% round(2)
or$`ci_low (2.5)` <- or$`ci_low (2.5)` %>% round(2)
or$`ci_high (97.5)` <- or$`ci_high (97.5)` %>% round(2)
or$results <- paste0(or$oddsratio, " (", or$`ci_low (2.5)`, " - ", or$`ci_high (97.5)`, ")")

#prepare predicted proportions for table
d_stim_p$p <- d_stim_p$p %>%  round(2)
d_stim_p$lower.CL <- d_stim_p$lower.CL %>%  round(2)
d_stim_p$upper.CL <- d_stim_p$upper.CL %>%  round(2)
d_stim_p$results <- paste0(d_stim_p$p, " (", d_stim_p$lower.CL, " - ", d_stim_p$upper.CL, ")")



#build results table section
results_education <- data.frame(Education = c("Post-secondary education", "Lower education level",
"Missing"),
                      Agreed = c(nrow(d2 %>% filter(outcome == 1 & dem == 1)),
                            nrow(d2 %>% filter(outcome == 1 & dem == 0)),
                            nrow(d2 %>% filter(outcome == 1 & is.na(dem)))),
                      Not_agreed = c(nrow(d2 %>% filter(outcome == 0 & dem == 1)),
                            nrow(d2 %>% filter(outcome == 0 & dem == 0)),
                            nrow(d2 %>% filter(outcome == 0 & is.na(dem)))),
                      Proportion_agreed_confidence_interval = c(d_stim_p$results[2],
d_stim_p$results[1], ""),
```

```
                    p_value = c(round(p, 5), "reference", ""),
                    Odds_ratio_confidence_interval = c(or$results[1], "reference", "")
                    )



#Country: high or low income
d2$dem <- d2$country_income_group
glm1 <- glm(outcome ~ dem, family = binomial(),
        data = d2)
summary(glm1) #not associated
model <- summary(glm1)
pa <- model$coefficients[,4] %>% as.data.frame()
p <- pa[2,1]

#look at predicted plots
d_stim <- data.frame(dem = factor(sample(c(0,1), 2, replace = F)))
d_stim <- d_stim[order(d_stim$dem),] %>% as.data.frame()
colnames(d_stim) <- "dem"
#to obtain the correct CIs I have predicted using type = "link", giving mean and se on log scales,
claculated CIs and then back-transformed
fit <- predict(glm1, d_stim, type = "link", se.fit = T)
d_stim$emmean <- exp(fit$fit)/(1+exp(fit$fit))
d_stim$lower.CL <- exp(fit$fit-1.96*fit$se.fit)/(1+exp(fit$fit-1.96*fit$se.fit))
d_stim$upper.CL <- exp(fit$fit+1.96*fit$se.fit)/(1+exp(fit$fit+1.96*fit$se.fit))
d_stim
d_stim_p <- d_stim
d_stim_p <- d_stim_p %>% dplyr::rename("p" = "emmean")

gg1 <- ggplot(d_stim_p, aes(x = dem, y = p)) +
  geom_point() +
```

```
  geom_errorbar(aes(ymin=lower.CL, ymax=upper.CL), width = 0.2) +
  labs(title = "MOOC outcome by participant country (model predictions)", y = "Proportion agreed")
+
  scale_x_discrete(labels = c("low income", "high income")) +
  theme(axis.title.x=element_blank())
gg1

#compare to raw data
t1 <- table(d2$outcome, d2$dem)
#proportion who agree
a <- t1[2,1]/(t1[1,1] + t1[2,1])
b <- t1[2,2]/(t1[1,2] + t1[2,2])
d3 <- data.frame(variable = c("dem0", "dem1"), value = c(a,b))

gg2 <- ggplot(d3, aes(x = variable, y = value)) +
  geom_point() +
  labs(title = "MOOC outcome by participant country (actual)", y = "Proportion agreed")  +
  scale_x_discrete(labels = c("low income", "high income")) +
  theme(axis.title.x=element_blank())
gg2

#calculate odds ratios
or <- or_glm(data = d2, model = glm1)

#prepare OR and CI for results table
or$oddsratio <- or$oddsratio %>% round(2)
or$`ci_low (2.5)` <- or$`ci_low (2.5)` %>% round(2)
or$`ci_high (97.5)` <- or$`ci_high (97.5)` %>% round(2)
or$results <- paste0(or$oddsratio, " (", or$`ci_low (2.5)`, " - ", or$`ci_high (97.5)`, ")")

#prepare predicted proportions for table
```

```
d_stim_p$p <- d_stim_p$p %>%  round(2)

d_stim_p$lower.CL <- d_stim_p$lower.CL %>%  round(2)

d_stim_p$upper.CL <- d_stim_p$upper.CL %>%  round(2)

d_stim_p$results <- paste0(d_stim_p$p, " (", d_stim_p$lower.CL, " - ", d_stim_p$upper.CL, ")")




#build results table section

results_country <- data.frame(`Country of residence` = c("High income", "Low or middle income",
"Missing"),

                    Agreed = c(nrow(d2 %>% filter(outcome == 1 & dem == 1)),

                          nrow(d2 %>% filter(outcome == 1 & dem == 0)),

                          nrow(d2 %>% filter(outcome == 1 & is.na(dem)))),

                    Not_agreed = c(nrow(d2 %>% filter(outcome == 0 & dem == 1)),

                          nrow(d2 %>% filter(outcome == 0 & dem == 0)),

                          nrow(d2 %>% filter(outcome == 0 & is.na(dem)))),

                    Proportion_agreed_confidence_interval = c(d_stim_p$results[2],
d_stim_p$results[1], ""),

                    p_value = c(round(p, 5), "reference", ""),

                    Odds_ratio_confidence_interval = c(or$results[1], "reference", "")

                    )





#age

gam1b <- gam(outcome ~ s(age, bs = "tp"), d2, family = binomial())

b0 <- expand.grid(age = seq(18, 100, length = 20))

fit <- predict(gam1b, b0, se.fit = TRUE)


b0$emmean <- exp(fit$fit)/(1+exp(fit$fit))

b0$lower.CL <- exp(fit$fit-1.96*fit$se.fit)/(1+exp(fit$fit-1.96*fit$se.fit))
```

```
b0$upper.CL <- exp(fit$fit+1.96*fit$se.fit)/(1+exp(fit$fit+1.96*fit$se.fit))

b0


#extract p vallue for smooothed age

model <- summary(gam1b)

p <- model$s.table[,4]


ageplot <- ggplot(b0, aes(x = age, y = emmean)) +

  geom_ribbon(aes(ymin = lower.CL, ymax = upper.CL), alpha = .1, colour = NA) +

  geom_line() +

  labs(title = "MOOC outcome by participant age", x = "Participant age", y = "Proportion complete")

ageplot


#calculate odds-ratios at 25 yeah cut-points

a <- or_gam(data = d2, model = gam1b, pred = "age", values = c(25,50))

a$comparison <- paste0(a$value1, " vs ", a$value2)

a$or <- paste0(round(a$oddsratio, 2), " (", round(a$`CI_low (2.5%)`, 2), " - ", round(a$`CI_high
(97.5%)`, 2), ")")


b <- or_gam(data = d2, model = gam1b, pred = "age", values = c(50,70))

b$comparison <- paste0(b$value1, " vs ", b$value2)

b$or <- paste0(round(b$oddsratio, 2), " (", round(b$`CI_low (2.5%)`, 2), " - ", round(b$`CI_high
(97.5%)`, 2), ")")




c <- or_gam(data = d2, model = gam1b, pred = "age", values = c(70, 90))

c$comparison <- paste0(c$value1, " vs ", c$value2)

c$or <- paste0(round(c$oddsratio, 2), " (", round(c$`CI_low (2.5%)`, 2), " - ", round(c$`CI_high
(97.5%)`, 2), ")")


#caluclate mean and st dev age for agree, along with %na

agree <- d2 %>% filter(outcome == "1" )
```

```
mean_agree <- mean(agree$age, na.rm = T)
sd_agree <- sd(agree$age, na.rm = T)
mean_sd_agree <- paste0(round(mean_agree, 2), " (", round(sd_agree, 2), ")")

n_missing_agree <- agree %>% filter(is.na(age)) %>% nrow()
perc_missing_agree <- n_missing_agree/(nrow(agree))*100
missing_agree <- paste0(n_missing_agree, " (", round(perc_missing_agree, 2), ")")



#caluclate mean and st dev age for not agree, along with %na
disagree <- d2 %>% filter(outcome == "0" )
mean_disagree <- mean(disagree$age, na.rm = T)
sd_disagree <- sd(disagree$age, na.rm = T)
mean_sd_disagree <- paste0(round(mean_disagree, 2), " (", round(sd_disagree, 2), ")")

n_missing_disagree <- disagree %>% filter(is.na(age)) %>% nrow()
perc_missing_disagree <- n_missing_agree/(nrow(disagree))*100
missing_disagree <- paste0(n_missing_disagree, " (", round(perc_missing_disagree, 2), ")")

#build results table section
results_age <- data.frame(`Age` = c("Age", "Mean (standard deviation)", "Missing, n (%)"),
                          Agreed = c("", mean_sd_agree, missing_agree),
                        Not_agreed = c("", mean_sd_disagree, missing_disagree),
                        p_value = c(round(p, 5), "", ""),
                        Age_comparison_years = c(a$comparison, b$comparison, c$comparison),
                        Odds_ratio_confidence_interval = c(a$or, b$or, c$or))




#save outputs into a new folder
if(!dir.exists(paste0(outcome_title, "_analysis"))){
```

```
dir.create(paste0(outcome_title, "_analysis"))}


write.csv(results_gender, file = file.path(getwd(), paste0(outcome_title, "_analysis"),
"results_gender.csv"))

write.csv(results_age, file = file.path(getwd(), paste0(outcome_title, "_analysis"), "results_age.csv"))

write.csv(results_country, file = file.path(getwd(), paste0(outcome_title, "_analysis"),
"results_country.csv"))

write.csv(results_occupation, file = file.path(getwd(), paste0(outcome_title, "_analysis"),
"results_occupation.csv"))

write.csv(results_education, file = file.path(getwd(), paste0(outcome_title, "_analysis"),
"results_education.csv"))


}


###run for each outcome

#assign outcome variable

d2$outcome <- d2$outcome_understanding

#run function

individual_demographic_models(d2 = d2, outcome = outcome, outcome_title =
"outcome_understanding")


#assign outcome variable

d2$outcome <- d2$outcome_info_risk_reduction

#run function

individual_demographic_models(d2 = d2, outcome = outcome, outcome_title =
"outcome_info_risk_reduction")


#assign outcome variable

d2$outcome <- d2$outcome_behaviour_lifestyle

#run function

individual_demographic_models(d2 = d2, outcome = outcome, outcome_title =
"outcome_behaviour_lifestyle")
```

```
#assign outcome variable

d2$outcome <- d2$outcome_already_applied

#run function

individual_demographic_models(d2 = d2, outcome = outcome, outcome_title =
"outcome_already_applied")


#assign outcome variable

d2$outcome <- d2$outcome_recommend

#run function

individual_demographic_models(d2 = d2, outcome = outcome, outcome_title =
"outcome_recommend")


#assign outcome variable

d2$outcome <- d2$learning_satisfied

#run function

individual_demographic_models(d2 = d2, outcome = outcome, outcome_title = "learning_satisfied")



#see what relationships remain significant when you adjust for all confounders

gam1 <- gam(completed ~ s(age, bs = "tp") + mooc + country_income_group + anzsic_div_q_occ +
post_secondary_edu + gender, d2, family = binomial())

summary(gam1) #all relationships remain significant!



####Create combined plot for categorical variables

gg5 <- ggarrange(gg1, gg2, gg3, gg4,

        labels = c("A", "B", "C", "D"),

        ncol = 2, nrow = 2)

#####################################


#use glms to mdoel each variable seperately

#gender
```

```
glm1 <- glm(completed ~ gender, family = binomial(),
            data = d2)
summary(glm1) #not associated


d_stim <- data.frame(gender = factor(sample(c(0,1), 2, replace = F)))
#to obtain the correct CIs I have predicted using type = "link", giving mean and se on log scales, claculated CIs and then back-transformed
fit <- predict(glm1, d_stim, type = "link", se.fit = T)
d_stim$emmean <- exp(fit$fit)/(1+exp(fit$fit))
d_stim$lower.CL <- exp(fit$fit-1.96*fit$se.fit)/(1+exp(fit$fit-1.96*fit$se.fit))
d_stim$upper.CL <- exp(fit$fit+1.96*fit$se.fit)/(1+exp(fit$fit+1.96*fit$se.fit))
d_stim
d_stim_p <- d_stim
d_stim_p <- d_stim_p %>% dplyr::rename("p" = "emmean")


gg1 <- ggplot(d_stim_p, aes(x = gender, y = p)) +
  geom_point() +
  geom_errorbar(aes(ymin=lower.CL, ymax=upper.CL), width = 0.2) +
  labs(title = "MOOC completion by participant gender", y = "Proportion completed")  +
  scale_x_discrete(labels = c("Male", "Female")) +
  scale_y_continuous(breaks = c(0.60, 0.62, 0.64, 0.66, 0.68, 0.70), limits = c(0.6, 0.7)) +
  theme(axis.title.x=element_blank())
gg1


#calculate odds ratios
or <- or_glm(data = d2, model = glm1)


#prepare OR and CI for results table
or$oddsratio <- or$oddsratio %>% round(2)
or$`ci_low (2.5)` <- or$`ci_low (2.5)` %>% round(2)
or$`ci_high (97.5)` <- or$`ci_high (97.5)` %>% round(2)
```

```r
or$results <- paste0(or$oddsratio, " (", or$`ci_low (2.5)`, " - ", or$`ci_high (97.5)`, ")")

#build results table section
results_gender <- data.frame(Gender = c("Male, Female", "Missing"),
                             Agree = c())

#education
glm2 <- glm(completed ~ post_secondary_edu, family = binomial(),
            data = d2)
summary(glm2) #highly associated

d_stim <- data.frame(post_secondary_edu = factor(sample(c(0,1), 2, replace = F)))
#to obtain the correct CIs I have predicted using type = "link", giving mean and se on log scales,
claculated CIs and then back-transformed
fit <- predict(glm2, d_stim, type = "link", se.fit = T)
d_stim$emmean <- exp(fit$fit)/(1+exp(fit$fit))
d_stim$lower.CL <- exp(fit$fit-1.96*fit$se.fit)/(1+exp(fit$fit-1.96*fit$se.fit))
d_stim$upper.CL <- exp(fit$fit+1.96*fit$se.fit)/(1+exp(fit$fit+1.96*fit$se.fit))
d_stim
d_stim_p <- d_stim
d_stim_p <- d_stim_p %>% dplyr::rename("p" = "emmean")

gg2 <- ggplot(d_stim_p, aes(x = post_secondary_edu, y = p)) +
  geom_point() +
  geom_errorbar(aes(ymin=lower.CL, ymax=upper.CL), width = 0.2) +
  labs(title = "MOOC completion by participant education level")  +
 scale_x_discrete(labels = c("Secondary education or less", "Post-secondary education")) +
  scale_y_continuous(breaks = c(0.60, 0.62, 0.64, 0.66, 0.68, 0.70), limits = c(0.6, 0.7)) +
  theme(axis.title.x=element_blank(),
        axis.title.y=element_blank())
gg2
```

```
#calculate odds ratios

or_glm(data = d2, model = glm2)



#occupation

glm3 <- glm(completed ~ anzsic_div_q_occ, family = binomial(),

          data = d2)

summary(glm3) #associated


d_stim <- data.frame(anzsic_div_q_occ = factor(sample(c(0,1), 2, replace = F)))

#to obtain the correct CIs I have predicted using type = "link", giving mean and se on log scales,
claculated CIs and then back-transformed

fit <- predict(glm3, d_stim, type = "link", se.fit = T)

d_stim$emmean <- exp(fit$fit)/(1+exp(fit$fit))

d_stim$lower.CL <- exp(fit$fit-1.96*fit$se.fit)/(1+exp(fit$fit-1.96*fit$se.fit))

d_stim$upper.CL <- exp(fit$fit+1.96*fit$se.fit)/(1+exp(fit$fit+1.96*fit$se.fit))

d_stim

d_stim_p <- d_stim

d_stim_p <- d_stim_p %>% dplyr::rename("p" = "emmean")


gg3 <- ggplot(d_stim_p, aes(x = anzsic_div_q_occ, y = p)) +

  geom_point() +

  geom_errorbar(aes(ymin=lower.CL, ymax=upper.CL), width = 0.2) +

  labs(title = "MOOC completion by participant occupation", x = "Participant occupation", y =
"Proportion completed") +

  scale_x_discrete(labels = c("Non-health occupation", "Health occupation")) +

  scale_y_continuous(breaks = c(0.60, 0.62, 0.64, 0.66, 0.68, 0.70), limits = c(0.6, 0.7)) +

  theme(axis.title.x=element_blank())

gg3


#calculate odds ratios
```

```
or_glm(data = d2, model = glm3)




#country

glm4 <- glm(completed ~ country_income_group, family = binomial(),
            data = d2)

summary(glm4) #highly associated


d_stim <- data.frame(country_income_group = factor(sample(c(0,1), 2, replace = F)))

#to obtain the correct CIs I have predicted using type = "link", giving mean and se on log scales, claculated CIs and then back-transformed

fit <- predict(glm4, d_stim, type = "link", se.fit = T)

d_stim$emmean <- exp(fit$fit)/(1+exp(fit$fit))

d_stim$lower.CL <- exp(fit$fit-1.96*fit$se.fit)/(1+exp(fit$fit-1.96*fit$se.fit))

d_stim$upper.CL <- exp(fit$fit+1.96*fit$se.fit)/(1+exp(fit$fit+1.96*fit$se.fit))

d_stim

d_stim_p <- d_stim

d_stim_p <- d_stim_p %>% dplyr::rename("p" = "emmean")


gg4 <- ggplot(d_stim_p, aes(x = country_income_group, y = p)) +
  geom_point() +
  geom_errorbar(aes(ymin=lower.CL, ymax=upper.CL), width = 0.2) +
  labs(title = "MOOC completion by participant country of residence wealth", x = "Participant country of residence", y = "Proportion completed") +
  scale_x_discrete(labels = c("Low or middle income country", "High income country")) +
  scale_y_continuous(breaks = c(0.60, 0.62, 0.64, 0.66, 0.68, 0.70), limits = c(0.6, 0.7)) +
  theme(axis.title.x=element_blank(),
        axis.title.y=element_blank())

gg4


#calculate odds ratios
```

```
or_glm(data = d2, model = glm4)



#mooc iteration of enrolment

glm5 <- glm(completed ~ mooc, family = binomial(),
            data = d2)

summary(glm5) #highly associated


d_stim <- data.frame(mooc = (sample(c("2016_07", "2017_03", "2018_05", "2018_10", "2019_05", "2019_10", "2020_05"), 7, replace = F)))

#to obtain the correct CIs I have predicted using type = "link", giving mean and se on log scales, claculated CIs and then back-transformed

fit <- predict(glm5, d_stim, type = "link", se.fit = T)

d_stim$emmean <- exp(fit$fit)/(1+exp(fit$fit))

d_stim$lower.CL <- exp(fit$fit-1.96*fit$se.fit)/(1+exp(fit$fit-1.96*fit$se.fit))

d_stim$upper.CL <- exp(fit$fit+1.96*fit$se.fit)/(1+exp(fit$fit+1.96*fit$se.fit))

d_stim

d_stim_p <- d_stim

d_stim_p <- d_stim_p %>% dplyr::rename("p" = "emmean")


ggplot(d_stim_p, aes(x = mooc, y = p)) +
  geom_point() +
  geom_errorbar(aes(ymin=lower.CL, ymax=upper.CL), width = 0.2) +
  labs(title = "MOOC completion by MOOC iteration of enrolment", x = "MOOC iteration", y = "Proportion completed")


#calculate odds ratios

or_glm(data = d2, model = glm5)




#age
```

```
gam1b <- gam(factor(completed) ~ s(age, bs = "tp"), d2, family = binomial())

b0 <- expand.grid(age = seq(18, 100, length = 20))

fit <- predict(gam1b, b0, se.fit = TRUE)


b0$emmean <- exp(fit$fit)/(1+exp(fit$fit))

b0$lower.CL <- exp(fit$fit-1.96*fit$se.fit)/(1+exp(fit$fit-1.96*fit$se.fit))

b0$upper.CL <- exp(fit$fit+1.96*fit$se.fit)/(1+exp(fit$fit+1.96*fit$se.fit))

b0


age <- ggplot(b0, aes(x = age, y = emmean)) +

  geom_ribbon(aes(ymin = lower.CL, ymax = upper.CL), alpha = .1, colour = NA) +

  geom_line() +

  labs(title = "MOOC completion by participant age", x = "Participant age", y = "Proportion complete")

age


ggsave(file = "age.png", plot = age, device = png(), width = 8, height = 4)


#From inspection of the graph, trends change at aproximately age 35, 61, 74 and 91, so calculate OR at each of these points

or_gam(data = d2, model = gam1b, pred = "age", values = c(18,35))

or_gam(data = d2, model = gam1b, pred = "age", values = c(35,61))

or_gam(data = d2, model = gam1b, pred = "age", values = c(61,74))

or_gam(data = d2, model = gam1b, pred = "age", values = c(74, 91))

or_gam(data = d2, model = gam1b, pred = "age", values = c(91, 100))


#calculate odds-ratios at 25 yeah cut-points

or_gam(data = d2, model = gam1b, pred = "age", values = c(25,50))

or_gam(data = d2, model = gam1b, pred = "age", values = c(50,70))

or_gam(data = d2, model = gam1b, pred = "age", values = c(70, 90))
```

#see what relationships remain significant when you adjust for all confounders

gam1 <- gam(completed ~ s(age, bs = "tp") + mooc + country_income_group + anzsic_div_q_occ + post_secondary_edu + gender, d2, family = binomial())

summary(gam1) #all relationships remain significant!

####Create combined plot for categorical variables

gg5 <- ggarrange(gg1, gg2, gg3, gg4,

labels = c("A", "B", "C", "D"),

ncol = 2, nrow = 2)

ggsave(gg5, file = "Fig5.tiff", width=11, height=9, dpi=300, compression = 'lzw')

```

Summary stats and models for interest and experience variables

```{r}

load("combined_first_enrolment")

#recode variables for analysis

combined_first_enrolment$gender <- combined_first_enrolment$gender %>% recode("male" = "0", "female" = "1")

combined_first_enrolment$gender <- na_if(combined_first_enrolment$gender, "other")

combined_first_enrolment$gender <- factor(combined_first_enrolment$gender, levels = c("0", "1"))

combined_first_enrolment$post_secondary_edu <- factor(combined_first_enrolment$post_secondary_edu, levels = c("0", "1"))

combined_first_enrolment$country_income_group <- combined_first_enrolment$country_income_group %>% recode("High income" = "1", "Lower middle income" = "0", "Upper middle income" = "0", "Low income" = "0")

```
combined_first_enrolment$anzsic_div_q_occ <-
factor(combined_first_enrolment$anzsic_div_q_occ, levels = c("0", "1"))

d <- combined_first_enrolment %>% dplyr::select(user_id, mooc, completed, experience_family,
interest_decline, interest_dementia, interest_diagnosis, interest_improve, interest_inherit,
interest_reduce_risk, interest_worry, age, gender, post_secondary_edu, country_income_group,
anzsic_div_q_occ)

d$completed <- as.factor(d$completed)

d %>% dplyr::group_by(mooc, experience_family) %>% count() #I wasn't given any experience info in
the 2020 data, but we have for all other years

d %>% dplyr::group_by(mooc, interest_decline) %>% count() #not asked in 2017
d %>% dplyr::group_by(mooc, interest_dementia) %>% count() #not asked in 2017
d %>% dplyr::group_by(mooc, interest_diagnosis) %>% count() #not asked in 2017
d %>% dplyr::group_by(mooc, interest_improve) %>% count() #not asked in 2017
d %>% dplyr::group_by(mooc, interest_inherit) %>% count() #not asked in 2017
d %>% dplyr::group_by(mooc, interest_reduce_risk) %>% count() #not asked in 2017
d %>% dplyr::group_by(mooc, interest_worry) %>% count() #not asked in 2017

#remove 2020 from experience_family data
d1 <- d %>% dplyr::filter(mooc != "2020_05")
#graph experience_family
ggplot(d1, aes(x = experience_family, fill = completed)) +
  geom_bar(position = "dodge")

#experience family does not look to be realted to completion - test this with a glm
d1$experience_family <- d1$experience_family %>% replace_na("0")
m1 <- glm(completed ~ experience_family, d1, family = "binomial")
summary(m1) #relationship is significant

d_stim <- data.frame(experience_family = factor(sample(c(0,1), 2, replace = F)))
```

```
#to obtain the correct CIs I have predicted using type = "link", giving mean and se on log scales, claculated CIs and then back-transformed

fit <- predict(m1, d_stim, type = "link", se.fit = T)

d_stim$emmean <- exp(fit$fit)/(1+exp(fit$fit))

d_stim$lower.CL <- exp(fit$fit-1.96*fit$se.fit)/(1+exp(fit$fit-1.96*fit$se.fit))

d_stim$upper.CL <- exp(fit$fit+1.96*fit$se.fit)/(1+exp(fit$fit+1.96*fit$se.fit))

d_stim

d_stim_p <- d_stim

d_stim_p <- d_stim_p %>% dplyr::rename("p" = "emmean")


ggplot(d_stim_p, aes(x = experience_family, y = p)) +

  geom_point() +

  geom_errorbar(aes(ymin=lower.CL, ymax=upper.CL), width = 0.2) +

  labs(title = "MOOC completion by participants' family experience with dementia", x = "Participant family experience with dementia", y = "Proportion completed") +

  scale_x_discrete(labels = c("No family experience sepcified", "Family experience specified"))


#calculate odds ratios

or_glm(data = d1, model = m1)


#see if this is still significant after adjusting for confounders

m1a <- gam(completed ~ experience_family + mooc + s(age, bs = "tp") + gender + post_secondary_edu + country_income_group + anzsic_div_q_occc, d1, family = "binomial")

summary(m1a) #relationship remains not significant after adjusting for confounders




#remove 2017 from interest data

d2 <- d %>% dplyr::filter(mooc != "2017_03")


#graph interest categories
```

```
d3 <- d2 %>% pivot_longer(c(interest_decline, interest_dementia, interest_diagnosis, interest_improve, interest_inherit, interest_reduce_risk, interest_worry), names_to = "interest_cat")


d3$value <- d3$value %>% replace_na("0")

ggplot(d3, aes(x = interest_cat, fill = value)) +

  geom_bar(position = "dodge") +

  facet_wrap(d3$completed) #doesn't look to be any associations

#test this statistically, adjusting for MOOC iteration of enrolment

d4 <- d3 %>% pivot_wider(names_from = interest_cat, values_from = value)

m2 <- glm(completed ~ interest_decline + interest_dementia + interest_diagnosis + interest_improve + interest_inherit + interest_reduce_risk + interest_worry + mooc, d4, family = "binomial")

summary(m2) #wow - interest_decline, intrest_diognosis, interest_improve, interest_inherit, interest_reduce_risk, interest_worry are all associated (to varying degrees, in varying directions) with completion


#adjust this model for other demographic factors that are associated with completion

m3 <- gam(completed ~ interest_decline + interest_dementia + interest_diagnosis + interest_improve + interest_inherit + interest_reduce_risk + interest_worry + mooc + s(age, bs = "tp") + gender + post_secondary_edu + country_income_group + anzsic_div_q_occ, d4, family = "binomial")

summary(m3)

#interest decline, diagnosis, improve and reduce risk still associated with completion



#what if you model each of these individually, so they don't cover the effects of oeach other?

#interest_decline

glm3a <- glm(completed ~ interest_decline, d4, family = "binomial")

summary(glm3a)


d_stim <- data.frame(interest_decline = factor(sample(c(0,1), 2, replace = F)))

#to obtain the correct CIs I have predicted using type = "link", giving mean and se on log scales, claculated CIs and then back-transformed
```

```
fit <- predict(glm3a, d_stim, type = "link", se.fit = T)

d_stim$emmean <- exp(fit$fit)/(1+exp(fit$fit))

d_stim$lower.CL <- exp(fit$fit-1.96*fit$se.fit)/(1+exp(fit$fit-1.96*fit$se.fit))

d_stim$upper.CL <- exp(fit$fit+1.96*fit$se.fit)/(1+exp(fit$fit+1.96*fit$se.fit))

d_stim

d_stim_p <- d_stim

d_stim_p <- d_stim_p %>% dplyr::rename("p" = "emmean")


ggplot(d_stim_p, aes(x = interest_decline, y = p)) +

  geom_point() +

  geom_errorbar(aes(ymin=lower.CL, ymax=upper.CL), width = 0.2) +

  labs(title = "MOOC completion by participants reasons for participating", x = "I feel my memory or other thinking skills are getting worse", y = "Proportion completed") +

  scale_x_discrete(labels = c("Affirmative", "Not affirmative"))


#calculate odds ratios

or_glm(data = d4, model = glm3a)


m3a <- gam(completed ~ interest_decline  + mooc + s(age, bs = "tp") + gender + post_secondary_edu + country_income_group + anzsic_div_q_occ, d4, family = "binomial")

summary(m3a)

#significant negative association



#interest_dementia

glm3b <- glm(completed ~ interest_dementia, d4, family = "binomial")

summary(glm3b)


d_stim <- data.frame(interest_dementia = factor(sample(c(0,1), 2, replace = F)))

#to obtain the correct CIs I have predicted using type = "link", giving mean and se on log scales, claculated CIs and then back-transformed

fit <- predict(glm3b, d_stim, type = "link", se.fit = T)
```

```
d_stim$emmean <- exp(fit$fit)/(1+exp(fit$fit))

d_stim$lower.CL <- exp(fit$fit-1.96*fit$se.fit)/(1+exp(fit$fit-1.96*fit$se.fit))

d_stim$upper.CL <- exp(fit$fit+1.96*fit$se.fit)/(1+exp(fit$fit+1.96*fit$se.fit))

d_stim

d_stim_p <- d_stim

d_stim_p <- d_stim_p %>% dplyr::rename("p" = "emmean")


ggplot(d_stim_p, aes(x = interest_dementia, y = p)) +

  geom_point() +

  geom_errorbar(aes(ymin=lower.CL, ymax=upper.CL), width = 0.2) +

  labs(title = "MOOC completion by participants reasons for participating", x = "I think I may be getting dementia", y = "Proportion completed") +

  scale_x_discrete(labels = c("Affirmative", "Not affirmative"))


#calculate odds ratios

or_glm(data = d4, model = glm3b)


m3b <- gam(completed ~ interest_dementia + mooc + s(age, bs = "tp") + gender + post_secondary_edu + country_income_group + anzsic_div_q_occ, d4, family = "binomial")

summary(m3b)

#significant negative association, but would not survive Bonferroni



#interest_diagnosis

glm3c <- glm(completed ~ interest_diagnosis, d4, family = "binomial")

summary(glm3c)


d_stim <- data.frame(interest_diagnosis = factor(sample(c(0,1), 2, replace = F)))

#to obtain the correct CIs I have predicted using type = "link", giving mean and se on log scales, claculated CIs and then back-transformed

fit <- predict(glm3c, d_stim, type = "link", se.fit = T)

d_stim$emmean <- exp(fit$fit)/(1+exp(fit$fit))
```

```
d_stim$lower.CL <- exp(fit$fit-1.96*fit$se.fit)/(1+exp(fit$fit-1.96*fit$se.fit))

d_stim$upper.CL <- exp(fit$fit+1.96*fit$se.fit)/(1+exp(fit$fit+1.96*fit$se.fit))

d_stim

d_stim_p <- d_stim

d_stim_p <- d_stim_p %>% dplyr::rename("p" = "emmean")


ggplot(d_stim_p, aes(x = interest_diagnosis, y = p)) +

  geom_point() +

  geom_errorbar(aes(ymin=lower.CL, ymax=upper.CL), width = 0.2) +

  labs(title = "MOOC completion by participants reasons for participating", x = "I want information to take to my doctor", y = "Proportion completed") +

  scale_x_discrete(labels = c("Affirmative", "Not affirmative"))


#calculate odds ratios

or_glm(data = d4, model = glm3c)


m3c <- gam(completed ~ interest_diagnosis + mooc + s(age, bs = "tp") + gender + post_secondary_edu + country_income_group + anzsic_div_q_occc, d4, family = "binomial")

summary(m3c)

#no association


#interest_improve

glm3d <- glm(completed ~ interest_improve, d4, family = "binomial")

summary(glm3d)


d_stim <- data.frame(interest_improve = factor(sample(c(0,1), 2, replace = F)))

#to obtain the correct CIs I have predicted using type = "link", giving mean and se on log scales, claculated CIs and then back-transformed

fit <- predict(glm3d, d_stim, type = "link", se.fit = T)

d_stim$emmean <- exp(fit$fit)/(1+exp(fit$fit))
```

```
d_stim$lower.CL <- exp(fit$fit-1.96*fit$se.fit)/(1+exp(fit$fit-1.96*fit$se.fit))

d_stim$upper.CL <- exp(fit$fit+1.96*fit$se.fit)/(1+exp(fit$fit+1.96*fit$se.fit))

d_stim

d_stim_p <- d_stim

d_stim_p <- d_stim_p %>% dplyr::rename("p" = "emmean")


ggplot(d_stim_p, aes(x = interest_improve, y = p)) +
  geom_point() +
  geom_errorbar(aes(ymin=lower.CL, ymax=upper.CL), width = 0.2) +
  labs(title = "MOOC completion by participants reasons for participating", x = "I want to improve my memory or thinking skills", y = "Proportion completed") +
  scale_x_discrete(labels = c("Affirmative", "Not affirmative"))


#calculate odds ratios

or_glm(data = d4, model = glm3d)


m3d <- gam(completed ~ interest_improve + mooc + s(age, bs = "tp") + gender + post_secondary_edu + country_income_group + anzsic_div_q_occ, d4, family = "binomial")

summary(m3d)

#singificant positive association




#interest_inherit

glm3e <- glm(completed ~ interest_inherit, d4, family = "binomial")

summary(glm3e)


d_stim <- data.frame(interest_inherit = factor(sample(c(0,1), 2, replace = F)))

#to obtain the correct CIs I have predicted using type = "link", giving mean and se on log scales, claculated CIs and then back-transformed

fit <- predict(glm3e, d_stim, type = "link", se.fit = T)

d_stim$emmean <- exp(fit$fit)/(1+exp(fit$fit))

d_stim$lower.CL <- exp(fit$fit-1.96*fit$se.fit)/(1+exp(fit$fit-1.96*fit$se.fit))
```

```
d_stim$upper.CL <- exp(fit$fit+1.96*fit$se.fit)/(1+exp(fit$fit+1.96*fit$se.fit))

d_stim

d_stim_p <- d_stim

d_stim_p <- d_stim_p %>% dplyr::rename("p" = "emmean")


ggplot(d_stim_p, aes(x = interest_inherit, y = p)) +
  geom_point() +
  geom_errorbar(aes(ymin=lower.CL, ymax=upper.CL), width = 0.2) +
  labs(title = "MOOC completion by participants reasons for participating", x = "I think I may inherit dementia from my parent or grandparent", y = "Proportion completed") +
  scale_x_discrete(labels = c("Affirmative", "Not affirmative"))


#calculate odds ratios

or_glm(data = d4, model = glm3e)


m3e <- gam(completed ~ interest_inherit + mooc + s(age, bs = "tp") + gender + post_secondary_edu + country_income_group + anzsic_div_q_occ, d4, family = "binomial")

summary(m3e)

#no association




#interest_reduce_risk

glm3f <- glm(completed ~ interest_reduce_risk, d4, family = "binomial")

summary(glm3f)


d_stim <- data.frame(interest_reduce_risk = factor(sample(c(0,1), 2, replace = F)))

#to obtain the correct CIs I have predicted using type = "link", giving mean and se on log scales, claculated CIs and then back-transformed

fit <- predict(glm3f, d_stim, type = "link", se.fit = T)

d_stim$emmean <- exp(fit$fit)/(1+exp(fit$fit))

d_stim$lower.CL <- exp(fit$fit-1.96*fit$se.fit)/(1+exp(fit$fit-1.96*fit$se.fit))
```

```
d_stim$upper.CL <- exp(fit$fit+1.96*fit$se.fit)/(1+exp(fit$fit+1.96*fit$se.fit))

d_stim

d_stim_p <- d_stim

d_stim_p <- d_stim_p %>% dplyr::rename("p" = "emmean")


ggplot(d_stim_p, aes(x = interest_reduce_risk, y = p)) +

  geom_point() +

  geom_errorbar(aes(ymin=lower.CL, ymax=upper.CL), width = 0.2) +

  labs(title = "MOOC completion by participants reasons for participating", x = "I want to reduce my risk of dementia", y = "Proportion completed") +

  scale_x_discrete(labels = c("Affirmative", "Not affirmative"))


#calculate odds ratios

or_glm(data = d4, model = glm3f)


m3f <- gam(completed ~ interest_reduce_risk + mooc + s(age, bs = "tp") + gender + post_secondary_edu + country_income_group + anzsic_div_q_occ, d4, family = "binomial")

summary(m3f)

#significant positive association




#interest_worry

glm3g <- glm(completed ~ interest_worry, d4, family = "binomial")

summary(glm3g)


d_stim <- data.frame(interest_worry = factor(sample(c(0,1), 2, replace = F)))

#to obtain the correct CIs I have predicted using type = "link", giving mean and se on log scales, claculated CIs and then back-transformed

fit <- predict(glm3g, d_stim, type = "link", se.fit = T)

d_stim$emmean <- exp(fit$fit)/(1+exp(fit$fit))

d_stim$lower.CL <- exp(fit$fit-1.96*fit$se.fit)/(1+exp(fit$fit-1.96*fit$se.fit))
```

```
d_stim$upper.CL <- exp(fit$fit+1.96*fit$se.fit)/(1+exp(fit$fit+1.96*fit$se.fit))

d_stim

d_stim_p <- d_stim

d_stim_p <- d_stim_p %>% dplyr::rename("p" = "emmean")


ggplot(d_stim_p, aes(x = interest_worry, y = p)) +

  geom_point() +

  geom_errorbar(aes(ymin=lower.CL, ymax=upper.CL), width = 0.2) +

  labs(title = "MOOC completion by participants reasons for participating", x = "I worry about my chances of getting dementia", y = "Proportion completed") +

  scale_x_discrete(labels = c("Affirmative", "Not affirmative"))


#calculate odds ratios

or_glm(data = d4, model = glm3g)


m3g <- gam(completed ~ interest_worry + mooc + s(age, bs = "tp") + gender + post_secondary_edu + country_income_group + anzsic_div_q_occ, d4, family = "binomial")

summary(m3g)

#no association



#see if there's a relationship between interest_inherit and experience_family

ggplot(d, aes(x = interest_inherit, fill = experience_family)) +

  geom_bar(position = "dodge")

d5 <- d

d5$experience_family <- d5$experience_family %>% replace_na("0") %>% as.factor()

d5$interest_inherit <- d5$interest_inherit %>% replace_na("0") %>% as.factor()

d5 <- d5 %>% na_if("not_asked")

m4 <- glm(interest_inherit ~ experience_family, d5, family = "binomial")

summary(m4) #family experience is associated with interest_inherit


#tabule interest data
```

```
d4$interest_decline <- factor(d4$interest_decline,
                    levels = c("1","0"),
                    labels = c("I feel my memory or other thinking skills are getting worse", "0"))
d4$interest_dementia <- factor(d4$interest_dementia,
                    levels = c("1","0"),
                    labels = c("I think I may be getting dementia", "0"))
d4$interest_diagnosis <- factor(d4$interest_diagnosis,
                    levels = c("1","0"),
                    labels = c("I want information to take to my doctor", "0"))
d4$interest_improve <- factor(d4$interest_improve,
                    levels = c("1","0"),
                    labels = c("I want to improve my memory or thinking skills", "0"))
d4$interest_inherit <- factor(d4$interest_inherit,
                    levels = c("1","0"),
                    labels = c("I think I may inherit dementia from my parent or grandparent", "0"))
d4$interest_reduce_risk <- factor(d4$interest_reduce_risk,
                    levels = c("1","0"),
                    labels = c("I want to reduce my risk of dementia", "0"))
d4$interest_worry <- factor(d4$interest_worry,
                    levels = c("1","0"),
                    labels = c("I worry about my chances of getting dementia", "0"))
d4$completed <- factor(d4$completed,
                    levels = c("1","0"),
                    labels = c("Completed", "Not completed"))

t1 <- table1::table1(~interest_decline + interest_dementia + interest_diagnosis + interest_improve + interest_inherit + interest_reduce_risk + interest_worry |completed, d4)
t1

#table for experience_family
d4$interest_worry <- factor(d4$interest_worry,
```

```
                   levels = c("1","0"),
                   labels = c("I worry about my chances of getting dementia", "0"))
d1$completed <- factor(d1$completed,
                   levels = c("1","0"),
                   labels = c("Completed", "Not completed"))
d1$experience_family <- factor(d1$experience_family,
                   levels = c("1","0"),
                   labels = c("Relatives living with dementia", "0"))

t2 <- table1::table1(~experience_family |completed, d1)
t2
```

###TOPIC MODELLING

```
```{r}
load("combined_first_completion")

#use tidytext to unnest tokens, keeping only english language words
a <- combined_first_completion %>%
  unnest_tokens(word, outcome_already_applied_text)

#use tidytext to re-nest these tokens into clean original responses (produces "data" column containing these)
b <- a %>%
  nest(word)

#dplyr::rename clean responses column "text"
b<- b %>%
```
```

```
  dplyr::mutate(outcome_already_applied_text_cleaned = purrr::map(b$data, unlist),
         outcome_already_applied_text_cleaned =
purrr::map_chr(outcome_already_applied_text_cleaned, paste, collapse = " "))  %>%
  dplyr::select(-data)

#correct NA values
b$outcome_already_applied_text_cleaned <- b$outcome_already_applied_text_cleaned %>%
  na_if("NA")

#remove NA values
b <- b %>% dplyr::filter(!is.na(outcome_already_applied_text_cleaned))

#dplyr::rename all_users_quiz_tm and save
combined_first_completion_tm <- b

save(combined_first_completion_tm, file = "combined_first_completion_tm")
```

Topic model cleaned responses to application question on feedback survey

```{r}
load("combined_first_completion_tm")

already_applied_topics <- combined_first_completion_tm

analysis_name <- "application_Q_tm"

# set the seed for the random number generator
set.seed(123)

thoughts.table <- function(x){
  a <- data.frame(rep(paste0("Topic ", x), length(thoughts[[x]])),
```

```
                meta$y[thoughts.index[[x]]], meta$PostingUserId[thoughts.index[[x]]])
  names(a) <- c("topic", "origin", "userid")
  return(a)
}

ft <- function(x){
  findThoughts(fit, texts = as.character(out$meta$X2),
              n = 6, topics = x)
}

# define stop words
rm <- c(stopwords("en"))

# K is the number of topics to fit the model to
K <- c(21)
#originally fit 5 different numbers of topics and dplyr::selected 21 as the best number based on theta, semantic coherance and exclusivity
#K <- c(5,10,15,21,30)

posts <- already_applied_topics
posts$X1 <- already_applied_topics$outcome_already_applied_text_cleaned


#Analysis preparation
processed <- textProcessor(posts$X1, metadata = posts, removestopwords = TRUE, removenumbers = FALSE, stem = FALSE, onlycharacter = T, striphtml = T, verbose = F)
out <- prepDocuments(processed$documents, processed$vocab, processed$meta)
docs <- out$documents
vocab <- out$vocab
meta <-out$meta
proc_list <- list(processed, out, docs, vocab, meta)
save(proc_list, file = paste0(analysis_name, "_processed.RData"))
```

```
load(paste0(analysis_name, "_processed.RData"))

processed <- proc_list[[1]]

out <- proc_list[[2]]

docs <- proc_list[[3]]

vocab <- proc_list[[4]]

meta <- proc_list[[5]]


#Analysis

#Structural topic models were fitted according to prevalence of tokens. Models were fitted for *k* = `r K` topics and the results are reproduced for each analysis in this document. The fitted models are saved to file so that output for any model can be reproduced without the need to run the analysis again.


fit <- manyTopics(out$documents, out$vocab, K = K,

        prevalence =~ 1, max.em.its = 75,

        data = out$meta, runs = 5, init.type = "Spectral",

        M = 20,

        frexw = 0.15,

        verbose = FALSE)


save(fit, file = paste0(analysis_name, "_stm0.RData"))


load(paste0(analysis_name, "_stm0.RData"))


#Calculate parameters for quality graphs

N <- 20  # number of top exemplars to compute theta from

m <- extractFit(fit, documents = docs, n = N)


#Plots

#Semantic coherence x Exclusivity

ggplot(m, aes(x = scale(m$semcoh), y = scale(m$exclusivity), colour = m$topic)) +
```

```
  facet_wrap(~ K) +
  geom_point(alpha = 4/5)

#Semantic coherence x mean Theta
ggplot(m, aes(x = scale(m$semcoh), y = scale(m$theta), colour = m$topic)) +
  facet_wrap(~ K) +
  geom_point(alpha = 4/5)

#top words
for(i in 1:length(fit$out)){
  print(summary(fit$out[[i]]))
}

#top topics plots
plot(fit$out[[1]])
#plot(fit$out[[2]])
#plot(fit$out[[3]])
#plot(fit$out[[4]])
#plot(fit$out[[5]])

#Exemplars
thr = 0.1

for(i in fit$out){
  ft <- findThoughts0(i, texts = as.character(out$meta$X1),
                    n = N, thresh = thr)
  reflections_consent <- lapply(ft$index, function(x) data.frame(meta$PostingUserId[x],
meta$Text[x]))
  j <- reshape2::melt(ft$index)

  reflections <- data.frame(j$L1,
```

```
                    meta$user_id[j$value],
                    meta$X1[j$value],
                    round(unlist(ft$theta), 2))

  names(reflections) <- c("topic", "user_id", "text", "theta")
  print(kable(reflections))
}

#used the 21 topic model
```

```{r}
#extract original responses from key examplars
a <- all_users_quiz %>% dplyr::filter(user_id == "77918")
print(a$outcome_already_applied_text)

a <- all_users_quiz %>% dplyr::filter(user_id == "562748")
print(a$outcome_already_applied_text)

a <- all_users_quiz %>% dplyr::filter(user_id == "229206")
print(a$outcome_already_applied_text)

a <- all_users_quiz %>% dplyr::filter(user_id == "253520")
print(a$outcome_already_applied_text)

a <- all_users_quiz %>% dplyr::filter(user_id == "141269")
print(a$outcome_already_applied_text)

```

Clean responses to the feedback survey question "What was the best thing about the Preventing Dementia MOOC?"

```{r}

load("combined_first_completion")

#use tidytext to unnest tokens, keeping only english language words

a <- combined_first_completion %>%

  unnest_tokens(word, impression_best)

#use tidytext to re-nest these tokens into clean original responses (produces "data" column containing these)

b <- a %>%

  nest(data = c(word))

#dplyr::rename clean responses column "text"

b<- b %>%

  dplyr::mutate(impression_best_cleaned = purrr::map(b$data, unlist),

        impression_best_cleaned = purrr::map_chr(impression_best_cleaned, paste, collapse = " ")) %>%

  dplyr::select(-data)

#correct NA values

b$impression_best_cleaned <- b$impression_best_cleaned %>%

  na_if("NA")

#remove NA values

b <- b %>% dplyr::filter(!is.na(impression_best_cleaned))

#dplyr::rename feedback_posts_tm and save

impression_best_tm <- b
```

```
save(impression_best_tm, file = "impression_best_tm")
```
```

Topic model cleaned responses to impression_best question on feedback survey

```
```{r}
load("impression_best_tm")

already_applied_topics <- impression_best_tm

analysis_name <- "impression_best_tm"

# set the seed for the random number generator
set.seed(123)

thoughts.table <- function(x){
  a <- data.frame(rep(paste0("Topic ", x), length(thoughts[[x]])),
                  meta$y[thoughts.index[[x]]], meta$PostingUserId[thoughts.index[[x]]])
  names(a) <- c("topic", "origin", "userid")
  return(a)
}

ft <- function(x){
  findThoughts(fit, texts = as.character(out$meta$X2),
               n = 6, topics = x)
}

# define stop words
rm <- c(stopwords("en"))

# K is the number of topics to fit the model to
#K <- c(5,10,15,21,30)
```

#originally fit 5 different numbers of topics and dplyr::selected 21 as the best number based on theta, semantic coherance and exclusivity

K <- c(21)

posts <- impression_best_tm

posts$X1 <- impression_best_tm$impression_best_cleaned

#Analysis preparation

processed <- textProcessor(posts$X1, metadata = posts, removestopwords = TRUE, removenumbers = FALSE, stem = FALSE, onlycharacter = T, striphtml = T, verbose = F)

out <- prepDocuments(processed$documents, processed$vocab, processed$meta)

docs <- out$documents

vocab <- out$vocab

meta <-out$meta

proc_list <- list(processed, out, docs, vocab, meta)

save(proc_list, file = paste0(analysis_name, "_processed.RData"))

load(paste0(analysis_name, "_processed.RData"))

processed <- proc_list[[1]]

out <- proc_list[[2]]

docs <- proc_list[[3]]

vocab <- proc_list[[4]]

meta <- proc_list[[5]]

#Analysis

#Structural topic models were fitted according to prevalence of tokens. Models were fitted for *k* = `r K` topics and the results are reproduced for each analysis in this document. The fitted models are saved to file so that output for any model can be reproduced without the need to run the analysis again.

fit <- manyTopics(out$documents, out$vocab, K = K,

    prevalence =~ 1, max.em.its = 75,

```
            data = out$meta, runs = 5, init.type = "Spectral",
            M = 20,
            frexw = 0.15,
            verbose = FALSE)

save(fit, file = paste0(analysis_name, "_stm0.RData"))

load(paste0(analysis_name, "_stm0.RData"))

#Calculate parameters for quality graphs
N <- 20  # number of top exemplars to compute theta from
m <- extractFit(fit, documents = docs, n = N)

#Plots
#Semantic coherence x Exclusivity
ggplot(m, aes(x = scale(m$semcoh), y = scale(m$exclusivity), colour = m$topic)) +
  facet_wrap(~ K) +
  geom_point(alpha = 4/5)

#Semantic coherence x mean Theta
ggplot(m, aes(x = scale(m$semcoh), y = scale(m$theta), colour = m$topic)) +
  facet_wrap(~ K) +
  geom_point(alpha = 4/5)

#top words
for(i in 1:length(fit$out)){
  print(summary(fit$out[[i]]))
}

#top topics plots
plot(fit$out[[1]])
```

```r
#plot(fit$out[[2]])

#plot(fit$out[[3]])

#plot(fit$out[[4]])

#plot(fit$out[[5]])


#Exemplars

thr = 0.1


for(i in fit$out){

  ft <- findThoughts0(i, texts = as.character(out$meta$X1),

                    n = N, thresh = thr)

  reflections_consent <- lapply(ft$index, function(x) data.frame(meta$PostingUserId[x],
meta$Text[x]))

  j <- reshape2::melt(ft$index)


  reflections <- data.frame(j$L1,

                         meta$user_id[j$value],

                         meta$X1[j$value],

                         round(unlist(ft$theta), 2))


  names(reflections) <- c("topic", "user_id", "text", "theta")

  print(kable(reflections))

}
```

```{r}

#pull out original text of exemplars


a <- combined_first_completion %>% dplyr::filter(user_id == "17345")

print(a$impression_best)


a <- combined_first_completion %>% dplyr::filter(user_id == "220252")
```

```
print(a$impression_best)

a <- combined_first_completion %>% dplyr::filter(user_id == "77116")

print(a$impression_best)

a <- combined_first_completion %>% dplyr::filter(user_id == "102149")

print(a$impression_best)

```

Clean responses to the feedback survey question "What was the worst thing about the Preventing Dementia MOOC?"

```{r}
load("combined_first_completion")



#use tidytext to unnest tokens, keeping only english language words

a <- combined_first_completion %>%

  unnest_tokens(word, impression_worst)


#use tidytext to re-nest these tokens into clean original responses (produces "data" column containing these)

b <- a %>%

  nest(data = c(word))


#dplyr::rename clean responses column "text"

b<- b %>%

  dplyr::mutate(impression_worst_cleaned = purrr::map(b$data, unlist),

        impression_worst_cleaned = purrr::map_chr(impression_worst_cleaned, paste, collapse = " ")) %>%

  dplyr::select(-data)


#correct NA values
```

~~~
b$impression_worst_cleaned <- b$impression_worst_cleaned %>%
  na_if("NA")

#remove NA values
b <- b %>% dplyr::filter(!is.na(impression_worst_cleaned))

#dplyr::rename feedback_posts_tm and save
impression_worst_tm <- b

save(impression_worst_tm, file = "impression_worst_tm")
```

Topic model cleaned responses to impression_best question on feedback survey
```{r}
load("impression_worst_tm")

already_applied_topics <- impression_worst_tm

analysis_name <- "impression_worst_tm"

# set the seed for the random number generator
set.seed(123)

thoughts.table <- function(x){
  a <- data.frame(rep(paste0("Topic ", x), length(thoughts[[x]])),
                  meta$y[thoughts.index[[x]]], meta$PostingUserId[thoughts.index[[x]]])
  names(a) <- c("topic", "origin", "userid")
  return(a)
}

ft <- function(x){
~~~

```
  findThoughts(fit, texts = as.character(out$meta$X2),
               n = 6, topics = x)
}

# define stop words
rm <- c(stopwords("en"))

# K is the number of topics to fit the model to
#K <- c(5,10,15,21,30)
#originally fit 5 different numbers of topics and dplyr::selected 21 as the best number based on theta, semantic coherance and exclusivity
K <- c(21)

posts <- impression_worst_tm
posts$X1 <- impression_worst_tm$impression_worst_cleaned



#Analysis preparation
processed <- textProcessor(posts$X1, metadata = posts, removestopwords = TRUE, removenumbers = FALSE, stem = FALSE, onlycharacter = T, striphtml = T, verbose = F)
out <- prepDocuments(processed$documents, processed$vocab, processed$meta)
docs <- out$documents
vocab <- out$vocab
meta <-out$meta
proc_list <- list(processed, out, docs, vocab, meta)
save(proc_list, file = paste0(analysis_name, "_processed.RData"))

load(paste0(analysis_name, "_processed.RData"))
processed <- proc_list[[1]]
out <- proc_list[[2]]
docs <- proc_list[[3]]
vocab <- proc_list[[4]]
```

meta <- proc_list[[5]]

#Analysis

#Structural topic models were fitted according to prevalence of tokens. Models were fitted for *k* = `r K` topics and the results are reproduced for each analysis in this document. The fitted models are saved to file so that output for any model can be reproduced without the need to run the analysis again.

```
fit <- manyTopics(out$documents, out$vocab, K = K,
        prevalence =~ 1, max.em.its = 75,
        data = out$meta, runs = 5, init.type = "Spectral",
        M = 20,
        frexw = 0.15,
        verbose = FALSE)

save(fit, file = paste0(analysis_name, "_stm0.RData"))

load(paste0(analysis_name, "_stm0.RData"))

#Calculate parameters for quality graphs
N <- 20  # number of top exemplars to compute theta from
m <- extractFit(fit, documents = docs, n = N)

#Plots
#Semantic coherence x Exclusivity
ggplot(m, aes(x = scale(m$semcoh), y = scale(m$exclusivity), colour = m$topic)) +
  facet_wrap(~ K) +
  geom_point(alpha = 4/5)

#Semantic coherence x mean Theta
ggplot(m, aes(x = scale(m$semcoh), y = scale(m$theta), colour = m$topic)) +
  facet_wrap(~ K) +
```

```
  geom_point(alpha = 4/5)

#top words
for(i in 1:length(fit$out)){
  print(summary(fit$out[[i]]))
}

#top topics plots
plot(fit$out[[1]])
#plot(fit$out[[2]])
#plot(fit$out[[3]])
#plot(fit$out[[4]])
#plot(fit$out[[5]])

#Exemplars
thr = 0.1

for(i in fit$out){
  ft <- findThoughts0(i, texts = as.character(out$meta$X1),
                     n = N, thresh = thr)
  reflections_consent <- lapply(ft$index, function(x) data.frame(meta$PostingUserId[x],
meta$Text[x]))
  j <- reshape2::melt(ft$index)

  reflections <- data.frame(j$L1,
                          meta$user_id[j$value],
                          meta$X1[j$value],
                          round(unlist(ft$theta), 2))

  names(reflections) <- c("topic", "user_id", "text", "theta")
  print(kable(reflections))
```

````
}
```

```{r}

a <- combined_first_completion %>% dplyr::filter(user_id == "561492")
print(a$impression_worst)

a <- combined_first_completion %>% dplyr::filter(user_id == "187223")
print(a$impression_worst)


a <- combined_first_completion %>% dplyr::filter(user_id == "553377")
print(a$impression_worst)

a <- combined_first_completion %>% dplyr::filter(user_id == "42941")
print(a$impression_worst)

a <- combined_first_completion %>% dplyr::filter(user_id == "106186")
print(a$impression_worst)

```
````
````
