# Supplementary material for: Educating the masses to address a global public health priority: The Preventing Dementia Massive Open Online Course (MOOC)
Source: PLoS One. 2022 May 4;17(5):e0267205. doi: 10.1371/journal.pone.0267205 (PMC9067672; doi:10.1371/journal.pone.0267205)
Supplement: S1 Table — (DOCX) [file pone.0267205.s002.docx]

**S1 Table. Associations between PDMOOC iteration and participant demographics.**

|  | **2016_07 (N=4,730)** | **2017_03 (N=5,979)** | **2018_05 (N=2,915)** | **2018_10 (N=8,509)** | **2019_05 (N=7,653)** | **2019_10 (N=10,235)** | **2020_05 (N=11,317)** |
| --- | --- | --- | --- | --- | --- | --- | --- |
| **Age** | | | | | | | |
| 25^th^ Quantile (confidence interval) | 41 (40.3 – 41.7) | 39 (38.1 – 39.9) | 38 (37.1 – 38.9) | 37 (36.5 – 37.5) | 36 (35.2 – 36.8) | 38 (37.3 – 38.7) | 33 (32.6 – 33.4) |
| p-value 25^th^ Quartile | *reference* | <0.001 | <0.001 | <0.001 | <0.001 | <0.001 | <0.001 |
| 50^th^ Quantile  (confidence interval) | 52 (51.4 – 52.6) | 52 (51.5 – 52.5) | 51 (50.3 – 51.7) | 50 (49.6 – 50.4) | 50 (49.6 – 50.4) | 53 (52.6 – 53.4) | 48 (47.6 – 48.4) |
| p-value 50^th^ Quartile | *reference* | - | 0.029 | <0.001 | <0.001 | 0.004 | <0.001 |
| 75^th^ Quantile  (confidence interval) | 59 (58.6 – 59.4) | 60 (59.7 – 60.3) | 59 (58.1 – 59.9) | 59 (58.5 – 59.5) | 59 (58.5 – 59.5) | 62 (61.8 – 62.2) | 60 (59.8 – 60.2) |
| p-value 75^th^ Quartile | *reference* | <0.001 | - | - | - | <0.001 | <0.001 |
| Missing, n (%) | 596 (12.6) | 184 (3.1) | 65 (2.2) | 213 (2.5) | 226 (3.0) | 500 (4.9) | 599 (5.3) |
| **Gender** | | | | | | | |
| Female, n (%) | 4178 (88.3) | 5109 (85.4) | 2526 (86.7) | 7502 (88.2) | 6686 (87.4) | 8519 (83.2) | 9582 (84.7) |
| Male, n (%) | 536 (11.3) | 858 (14.4) | 381 (13.1) | 985 (11.6) | 933 (12.2) | 1661 (16.2) | 1684 (14.9) |
| Proportion female (95% confidence interval) | 0.89  (0.88 – 0.90) | 0.86 (0.85 – 0.86) | 0.87 (0.86 – 0.88) | 0.88 (0.88 – 0.89) | 0.88 (0.87 – 0.88) | 0.84 (0.83 – 0.84) | 0.85 (0.84 – 0.86) |
| p-value | *reference* | <0.001 | 0.024* | - | - | <0.001* | <0.001* |
| Odds ratio - female  (95% confidence interval) | *reference* | 0.76 (0.68 – 0.86) | 0.85  (0.74 – 0.98) | 0.98 (0.87 – 0.1.09) | 0.92 (0.82 – 1.09) | 0.66  (0.59 – 0.73) | 0.73 (0.66 – 0.81) |
| Missing, n (%) | 16 (0.3) | 12 (0.2) | 8 (0.3) | 22 (0.3) | 34 (0.4) | 55 (0.5) | 51 (0.5) |
| **Occupation** | | | | | | | |
| Health/social occupation, n (%) | 3573 (75.5) | 3962 (66.3) | 2039 (69.9) | 5794 (68.1) | 4493 (58.7) | 5452 (53.3) | 7112 (62.8) |
| Non-health/social occupation, n (%) | 926 (19.6) | 1718 (28.7) | 733 (25.1) | 2300 (27.0) | 1716 (22.4) | 3225 (31.5) | 4041 (35.7) |
| Proportion health/social workers (95% confidence interval) | 0.79  (0.78 – 0.81) | 0.70 (0.69 – 0.71) | 0.74 (0.72 – 0.75) | 0.72  (0.71 – 0.73) | 0.72  (0.71 – 0.73) | 0.63 (0.62 – 0.64) | 0.64  (0.63-0.65) |
| p-value | *reference* | <0.001 | <0.001 | <0.001 | <0.001 | <0.001 | <0.001 |
| Odds ratio – health occupation  (95% confidence interval) | *reference* | 0.60  (0.55 – 0.66) | 0.72 (0.65 – 0.81) | 0.65 (0.60 – 0.71) | 0.68 (0.61 – 0.74) | 0.44 (0.40 – 0.48) | 0.46 (0.42 – 0.50) |
| Missing, n (%) | 231 (4.9) | 299 (5.0) | 143 (4.9) | 415 (4.9) | 1444 (18.9) | 1558 (15.2) | 164 (1.4) |
| **Education** | | | | | | | |
| Post-secondary education, n (%) | 3555 (75.2) | 4956 (82.9) | 2370 (81.3) | 6904 (81.1) | 5514 (72.1) | 7254 (70.9) | 9373 (82.8) |
| Secondary or lower education, n (%) | 1076 (22.7) | 1023 (17.1) | 545 (18.7) | 1603 (18.8) | 1055 (13.8) | 1398 (13.7) | 1690 (14.9) |
| Proportion post-secondary educated (95% confidence interval) | 0.77 (0.76 – 0.78) | 0.83 (0.82 – 0.84) | 0.81 (0.80 – 0.83) | 0.81 (0.80 – 0.82) | 0.84 (0.83 – 0.85) | 0.84 (0.83 – 0.85) | 0.85 (0.84 – 0.85) |
| p-value | *reference* | <0.001 | <0.001 | <0.001 | <0.001 | <0.001 | <0.001 |
| Odds ratio – Post-secondary education  (95% confidence interval) | *reference* | 1.47 (1.33 – 1.61) | 1.32 (1.17 – 1.48) | 1.30 (1.20 – 1.42) | 1.58 (1.44 – 1.74) | 1.57 (1.44 – 1.72) | 1.68 (1.54 – 1.83) |
| Missing, n (%) | 99 (2.1) | 0 (0) | 0 (0) | 2 (0.0) | 1084 (14.2) | 1583 (15.5) | 254 (2.2) |
| **Country of residence** | | | | | | | |
| High income country, n (%) | 4582 (96.9) | 5741 (96.0) | 2798 (96.0) | 8093 (95.1) | 6990 (91.3) | 9364 (91.5) | 10138 (89.6) |
| Low- or middle-income country, n (%) | 141 (3.0) | 227 (3.8) | 108 (3.7) | 402 (4.7) | 638 (8.3) | 842 (8.2) | 1157 (10.2) |
| Proportion high income country (95% confidence interval) | 0.97 (0.976– 0.97) | 0.96 (0.96 – 0.97) | 0.96 (0.96 – 0.97) | 0.95 (0.95 – 0.96) | 0.92 (0.91 – 0.92) | 0.92 (0.91 – 0.92) | 0.90 (0.89 – 0.90) |
| p-value | *reference* | 0.022* | - | <0.001 | <0.001 | <0.001 | <0.001 |
| Odds ratio – high income country  (95% confidence interval) | *reference* | 0.78 (0.63 – 0.96) | 0.80  (0.62 – 1.03) | 0.62 (0.51 – 0.75) | 0.34 (0.28 – 0.41) | 0.34 (0.28 – 0.41) | 0.27 (0.23 – 0.32) |
| Missing, n (%) | 7 (0.1) | 11 (0.2) | 9 (0.3) | 14 (0.2) | 25 (0.3) | 29 (0.3) | 22 (0.2) |
| *no longer significant after adjusting for confounders | | | | | | | |
