# Supplementary material for: Educating the masses to address a global public health priority: The Preventing Dementia Massive Open Online Course (MOOC)
Source: PLoS One. 2022 May 4;17(5):e0267205. doi: 10.1371/journal.pone.0267205 (PMC9067672; doi:10.1371/journal.pone.0267205)
Supplement: S2 Table — (DOCX) [file pone.0267205.s003.docx]

**S2 Table. Associations between completion of the PDMOOC and participant demographics, PDMOOC iteration, having a family history of dementia, and reasons for undertaking the PDMOOC.**

|  | **Not completed (N=17,482)** | **Completed (N=33,856)** | **p-value** | **Age comparisons (years)** | **Odds ratio  (confidence interval)** |
| --- | --- | --- | --- | --- | --- |
| **Age** |  |  | <0.001 | 25 vs 50 | 1.14 (1.13 – 1.15) |
| Mean (standard deviation) | 48.2 (15.2) | 49.2 (14.8) |  | 50 vs 70 | 0.99 (0.98 – 1.01) |
| Missing, n (%) | 901 (5.2) | 1482 (4.4) |  | 70 vs 90 | 0.85 (0.65 - 1.11) |

|  | **Not completed (N=17,482)**  **n (%)** | **Completed (N=33,856)**  **n (%)** | **Proportion completed  (confidence interval)** | **p-value** | **Odds ratio (confidence interval)** |
| --- | --- | --- | --- | --- | --- |
| **Gender** | | | | | |
| Male | 2328 (13.3) | 4710 (13.9) | 0.67 (0.66 - 0.68) | - | 1.05 (1.00 – 1.11) |
| Female | 15074 (86.2) | 29028 (85.7) | 0.66 (0.65 - 0.66) | *reference* | *reference* |
| Missing | 80 (0.5) | 118 (0.3) |  |  |  |
| **Occupation** | | | | | |
| Health occupation | 10608 (60.7) | 21817 (64.4) | 0.67 (0.67 – 0.68) | 0.002 | 1.14 (1.09 – 1.20) |
| Non-health occupation | 5005 (28.6) | 9654 (28.5) | 0.66 (0.65 – 0.67) | *reference* | *reference* |
| Missing | 1869 (10.7) | 2385 (7.0) |  |  |  |
| **Education** | | | | | |
| Post-secondary education | 13012 (74.4) | 26914 (79.5) | 0.67 (0.67 – 0.68) | <0.001 | 1.07 (1.02 – 1.11) |
| Lower level of education | 2987 (17.1) | 5403 (16.0) | 0.64 (0.63 – 0.65) | *reference* | *reference* |
| Missing | 1483 (8.5) | 1539 (4.5) |  |  |  |
| **Country of residence** | | | | | |
| High income | 16138 (92.3) | 31568 (93.2) | 0.66 (0.66 – 0.66) | <0.001 | 1.15 (1.07 – 1.24) |
| Low or middle income | 1302 (7.4) | 2213 (6.5) | 0.63 (0.61 – 0.65) | *reference* | *reference* |
| Missing | 42 (0.2) | 75 (0.2) |  |  |  |
| **PDMOOC iteration** | **n** | **n** |  |  |  |
| 2016_07 | 1797 | 2933 | 0.62 (0.61 – 0.63) | *reference* | *reference* |
| 2017_03 | 1734 | 4245 | 0.71 (0.70 – 0.72) | <0.001 | 1.50 (1.38 – 1.63) |
| 2018_05 | 665 | 2250 | 0.77 (0.76 – 0.79) | <0.001 | 2.07 (1.87 – 2.30) |
| 2018_10 | 3020 | 5489 | 0.65 (0.63 – 0.66) | 0.004 | 1.11 (1.03 – 1.20) |
| 2019_05 | 2553 | 5100 | 0.67 (0.66 – 0.68) | <0.001 | 1.22 (1.14 – 1.32) |
| 2019_10 | 4026 | 6209 | 0.61 (0.60 – 0.62) | - | 0.95 (0.88 – 1.01) |
| 2020_05 | 3687 | 7630 | 0.67 (0.67 – 0.68) | <0.001 | 1.27 (1.18 – 1.36) |

| **Reason for undertaking the PDMOOC**  *Data from all years excluding 2017* | **Completed (N=29,611)**  **n (%)** | **Not completed (N=15,748)**  **n (%)** | **Proportion completed  (confidence interval)** | **p-value** | **Odds ratio (confidence interval)** |
| --- | --- | --- | --- | --- | --- |
| **I feel my memory or other thinking skills are getting worse** |  |  |  |  |  |
| Affirmative | 6765 (22.8) | 3815 (24.2) | 0.64 (0.63 – 0.65) | <0.001 | 0.93 (0.89 – 0.97) |
| Not affirmative | 22846 (77.2) | 11933 (75.8) | 0.66 (0.65 – 0.66) | *reference* | *reference* |
| **I think I may be getting dementia** |  |  |  |  |  |
| Affirmative | 1152 (3.9) | 680 (4.3) | 0.63 (0.61 – 0.65) | 0.03 | 0.90 (0.81 – 0.99) |
| Not affirmative | 28459 (96.1) | 15068 (95.7) | 0.65 (0.65 – 0.66) | *reference* | *reference* |
| **I want information to take to my doctor** |  |  |  |  |  |
| Affirmative | 3883 (13.1) | 2131 (13.5) | 0.65 (0.63 – 0.66) | - | 0.96 (0.91 – 1.02) |
| Not affirmative | 25728 (86.9) | 13617 (86.5) | 0.65 (0.65 – 0.66) | *reference* | *reference* |
| **I want to improve my memory or thinking skills** |  |  |  |  |  |
| Affirmative | 20134 (68.0) | 10056 (63.9) | 0.67 (0.66 – 0.67) | <0.001 | 1.20 (1.56 – 1.25) |
| Not affirmative | 9477 (32.0) | 5692 (36.1) | 0.62 (0.62 – 0.63) | *reference* | *reference* |
| **I think I may inherit dementia from my parent or grandparent** |  |  |  |  |  |
| Affirmative | 5476 (18.5) | 2966 (18.8) | 0.65 (0.64 – 0.66) | - | 0.98 (0.93 – 1.03) |
| Not affirmative | 24135 (81.5) | 12782 (81.2) | 0.65 (0.65 – 0.66) | *reference* | *reference* |
| **I want to reduce my risk of dementia** |  |  |  |  |  |
| Affirmative | 20838 (70.4) | 10380 (65.9) | 0.67 (0.66 – 0.67) | <0.001 | 1.23 (1.18 – 1.28) |
| Not affirmative | 8773 (29.6) | 5368 (34.1) | 0.62 (0.61 – 0.63) | *reference* | *reference* |
| **I worry about my chances of getting dementia** |  |  |  |  |  |
| Affirmative | 12515 (42.3) | 6561 (41.7) | 0.66 (0.65 – 0.66) | - | 1.03 (0.99 – 1.07) |
| Not affirmative | 17096 (57.7) | 9187 (58.3) | 0.65 (0.64 – 0.66) | *reference* | *reference* |

| **Family history of dementia**  *Data from all years excluding 2020* | **Completed (N=26,226)**  **n (%)** | **Not completed (N=13,795)**  **n (%)** | **Proportion completed  (confidence interval)** | **p-value** | **Odds ratio (confidence interval)** |
| --- | --- | --- | --- | --- | --- |
| **Family history of dementia** |  |  |  |  |  |
| Affirmative | 9715 (37.0) | 4655 (33.7) | 0.68 (0.67 – 0.68) | <0.001* | 1.16 (1.11 – 1.21) |
| Not affirmative | 16511 (63.0) | 9140 (66.3) | 0.64 (0.64 – 0.65) | *reference* | *reference* |
| *no longer significant after adjusting for confounders | | | | | |
