# Supplementary material for: Educating the masses to address a global public health priority: The Preventing Dementia Massive Open Online Course (MOOC)
Source: PLoS One. 2022 May 4;17(5):e0267205. doi: 10.1371/journal.pone.0267205 (PMC9067672; doi:10.1371/journal.pone.0267205)
Supplement: S3 Table — (DOCX) [file pone.0267205.s004.docx]

**S3 Table. The five most common themes arising in each of the three structural topic models.**

| **Structural topic modelling topics** | | | **Interpretation from manual thematic analysis** | |
| --- | --- | --- | --- | --- |
| **Feedback survey question** | **Frequently co-occurring words** | **Most exclusive frequently co-occurring words** | **Theme identifier** | **Description** |
| If you have already applied your MOOC learning, please tell us how. | diet, healthy, eating, eat, exercising, regular, alcohol | alcohol, intake, foods, vegetables, eating, avoiding, drinks | Diet | Participants reported changing their food and drink consumption in a way that they believed would reduce their dementia risk |
|  | dementia, work, care, understanding, clients, understand, aged | care, understanding, aged, residents, facility, carers, unit | Tertiary prevention | Participants reported using risk-reduction strategies when caring for older people, particularly people living with dementia |
|  | exercise, physical, activity, increased, healthier, level, importance | increased, activity, physical, regime, exercise, level, healthier | Physical activity | Participants reported increasing their physical activity in a way that they believed would reduce their dementia risk |
|  | activities, increase, cognitive, study, looking, online, courses | activities, puzzles, increase, games, study, stimulating, online | Cognitive activity | Participants reported increasing their cognitive activity in a way that they believed would reduce their dementia risk |
|  | now, life, need, good, things, just, try | style, always, life, good, need, try, right | Additional motivating factor | Participants described dementia risk reduction as an additional motivating factor for maintaining or adopting an overall healthy lifestyle |
| What was the best thing about the Preventing Dementia MOOC? | dementia, risk, can, understanding, help, reduce, factors | risk, reduce, factors, ways, reducing, onset, developing | Knowledge and attitudes | Participants reported increased knowledge of dementia risk factors and increased positivity around dementia risk reduction |
|  | easy, informative, use, ease, follow, thing, best | informative, use, follow, enjoyable, interactive, layout, exists | Ease of use | Participants described the course as easy to navigate and the information as easy to understand |
|  | learning, time, new, available, something, overall, studying | new, studying, available, learning, spend, finishing, lockdown | Novelty | Participants enjoyed learning new, interesting, and relevant things through the course |
|  | information, content, presented, way, great, accessible, useful | accessible, presented, useful, manner, professional, content, great | Quality | Participants appreciated both the content and the presentation of that content as high-quality |
|  | online, understand, study, relevant, navigate, self, topics | self, paced, study, online, contents, convenient, materials | Accessibility | Participants appreciated the accessible and self-paced nature of this online course. |
| What was the worst thing about the Preventing Dementia MOOC? | nothing, end, fault, survey, comes, particular, coming | nothing, survey, report, brilliant, fault, comes, itâ | Lack of negative features | Participants reported that they did not identify any negative features of the course and that they were disappointed it had ended |
|  | think, anything, cant, find, dont, questions, negative | think, anything, cant, canâ, honestly, find, ask | Lack of negative features | Participants reported that they could not think of any negative features of the course |
|  | time, complete, work, trying, busy, weeks, due | complete, busy, finish, frame, fit, schedule, deadline | Time constraints | Participants reported having difficulty finding time to do the MOOC during the limited timeframe for completion |
|  | really, good, bad, enjoyed, say, maybe, wasnt | really, good, wasnt, comment, say, enjoyed, bad | Lack of negative features | Participants reported that they did not identify any negative features of the course, and re-iterated that they enjoyed the course |
|  | videos, read, able, reading, lot, access, found | read, reading, text, notes, transcripts, additional, download | Difficulty engaging | Participants reported having difficulty engaging with the course due to technical difficulties or preferred learning styles that that were not well catered for in the course. |
