# Supplementary material for: Educating the masses to address a global public health priority: The Preventing Dementia Massive Open Online Course (MOOC)
Source: PLoS One. 2022 May 4;17(5):e0267205. doi: 10.1371/journal.pone.0267205 (PMC9067672; doi:10.1371/journal.pone.0267205)
Supplement: S4 Table — (DOCX) [file pone.0267205.s005.docx]

**S4 Table: Associations between affirmation of the statement “I was satisfied with my MOOC learning experience” and participant demographics.** Note: *indicates significant individual finding was not significant in combined model; #indicates that finding was no longer significant after adjusting for multiple comparisons (6 models from one dataset).

|  | **Affirmed** | **Not affirmed** | **p-value** | **Age comparisons (years)** | **Odds ratio  (confidence interval)** |
| --- | --- | --- | --- | --- | --- |
| **Age** |  |  | 0.03977*# | 25 vs 50 | 1.40 (1.27 - 1.55) |
| Mean (standard deviation) | 52.69 (13.8) | 51.33 (14.72) |  | 50 vs 70 | 1.01 (1.06 - 0.97) |
| Missing, n (%) | 744 (4.42) | 35 (134.3) |  | 70 vs 90 | 1.06 (1.62 - 0.69) |

|  | **Affirmed** | **Not affirmed** | **Proportion affirmed  (confidence interval)** | **p-value** | **Odds ratio (confidence interval)** |
| --- | --- | --- | --- | --- | --- |
| **Gender** |  |  |  |  |  |
| Male | 2153 | 93 | 0.96 (0.95 - 0.97) | 0.00528 | 0.72 (0.58 - 0.91) |
| Female | 14625 | 457 | 0.97 (0.97 - 0.97) | *reference* | *reference* |
| Missing | 65 | 4 |  |  |  |
| **Occupation** |  |  |  |  |  |
| Health occupation | 10018 | 350 | 0.97 (0.96 - 0.97) | 0.00493 | 0.76 (0.62 - 0.92) |
| Non-health occupation | 5569 | 147 | 0.97 (0.97 - 0.98) | *reference* | *reference* |
| Missing | 1256 | 57 |  |  |  |
| **Education** |  |  |  |  |  |
| Post-secondary education | 13557 | 432 | 0.97 (0.97 - 0.97) | 0.60459 | 0.94 (0.72 - 1.19) |
| Lower level of education | 2481 | 74 | 0.97 (0.96 - 0.98) | *reference* | *reference* |
| Missing | 805 | 48 |  |  |  |
| **Country of residence** |  |  |  |  |  |
| High income | 15889 | 512 | 0.97 (0.97 - 0.97) | 0.04900*# | 1.39 (0.99 - 1.89) |
| Low or middle income | 918 | 41 | 0.96 (0.94 - 0.97) | *reference* | *reference* |
| Missing | 36 | 1 |  |  |  |
