# Supplementary material for: Educating the masses to address a global public health priority: The Preventing Dementia Massive Open Online Course (MOOC)
Source: PLoS One. 2022 May 4;17(5):e0267205. doi: 10.1371/journal.pone.0267205 (PMC9067672; doi:10.1371/journal.pone.0267205)
Supplement: S5 Table — (DOCX) [file pone.0267205.s006.docx]

**S5 Table: Associations between affirmation of the statement “My understanding of dementia prevention has improved” and participant demographics.**

|  | **Affirmed** | **Not affirmed** | **p-value** | **Age comparisons (years)** | **Odds ratio  (confidence interval)** |
| --- | --- | --- | --- | --- | --- |
| **Age** |  |  | 0.00172 | 25 vs 50 | 1.62 (1.48 - 1.78) |
| Mean (standard deviation) | 52.71 (13.74) | 49.68 (15.03) |  | 50 vs 70 | 1.30 (1.42 - 1.18) |
| Missing, n (%) | 736 (4.52) | 15 (279.85) |  | 70 vs 90 | 0.84 (3.01 - 0.23) |

|  | **Affirmed** | **Not affirmed** | **Proportion affirmed  (confidence interval)** | **p-value** | **Odds ratio (confidence interval)** |
| --- | --- | --- | --- | --- | --- |
| **Gender** |  |  |  |  |  |
| Male | 2098 | 36 | 0.98 (0.98 - 0.99) | 0.69825 | 0.93 (0.66 - 1.35) |
| Female | 14128 | 226 | 0.98 (0.98 - 0.99) | *reference* | *reference* |
| Missing | 65 | 1 |  |  |  |
| **Occupation** |  |  |  |  |  |
| Health occupation | 9682 | 161 | 0.98 (0.98 - 0.99) | 0.17226 | 0.82 (0.62 - 1.08) |
| Non-health occupation | 5398 | 74 | 0.99 (0.98 - 0.99) | *reference* | *reference* |
| Missing | 1211 | 28 |  |  |  |
| **Education** |  |  |  |  |  |
| Post-secondary education | 13117 | 208 | 0.98 (0.98 - 0.99) | 0.82574 | 1.04 (0.73 - 1.45) |
| Lower level of education | 2366 | 39 | 0.98 (0.98 - 0.99) | *reference* | *reference* |
| Missing | 808 | 16 |  |  |  |
| **Country of residence** |  |  |  |  |  |
| High income | 15375 | 242 | 0.98 (0.98 - 0.99) | 0.12178 | 1.44 (0.88 - 2.22) |
| Low or middle income | 883 | 20 | 0.98 (0.97 - 0.99) | *reference* | *reference* |
| Missing | 33 | 1 |  |  |  |
