# Supplementary material for: Educating the masses to address a global public health priority: The Preventing Dementia Massive Open Online Course (MOOC)
Source: PLoS One. 2022 May 4;17(5):e0267205. doi: 10.1371/journal.pone.0267205 (PMC9067672; doi:10.1371/journal.pone.0267205)
Supplement: S6 Table — (DOCX) [file pone.0267205.s007.docx]

**S6 Table: Associations between affirmation of the statement “The MOOC has given me the information I need to reduce my dementia risk” and participant demographics.**

|  | **Affirmed** | **Not affirmed** | **p-value** | **Age comparisons (years)** | **Odds ratio  (confidence interval)** |
| --- | --- | --- | --- | --- | --- |
| **Age** |  |  | 0.00003 | 25 vs 50 | 1.5 (1.39 - 1.62) |
| Mean (standard deviation) | 52.64 (13.68) | 52.8 (15.15) |  | 50 vs 70 | 0.72 (0.73 - 0.7) |
| Missing, n (%) | 715 (4.58) | 32 (78.57) |  | 70 vs 90 | 0.6 (0.88 - 0.41) |

|  | **Affirmed** | **Not affirmed** | **Proportion affirmed  (confidence interval)** | **p-value** | **Odds ratio (confidence interval)** |
| --- | --- | --- | --- | --- | --- |
| **Gender** |  |  |  |  |  |
| Male | 1960 | 167 | 0.92 (0.91 - 0.93) | 0.00000 | 0.64 (0.54 - 0.76) |
| Female | 13581 | 740 | 0.95 (0.94 - 0.95) | *reference* | *reference* |
| Missing | 63 | 3 |  |  |  |
| **Occupation** |  |  |  |  |  |
| Health occupation | 9309 | 511 | 0.95 (0.94 - 0.95) | 0.15012 | 1.11 (0.96 - 1.28) |
| Non-health occupation | 5144 | 314 | 0.94 (0.94 - 0.95) | *reference* | *reference* |
| Missing | 1151 | 85 |  |  |  |
| **Education** |  |  |  |  |  |
| Post-secondary education | 12562 | 730 | 0.95 (0.94 - 0.95) | 0.51789 | 0.94 (0.77 - 1.14) |
| Lower level of education | 2276 | 124 | 0.95 (0.94 - 0.96) | *reference* | *reference* |
| Missing | 766 | 56 |  |  |  |
| **Country of residence** |  |  |  |  |  |
| High income | 14717 | 863 | 0.94 (0.94 - 0.95) | 0.40586 | 0.88 (0.63 - 1.18) |
| Low or middle income | 856 | 44 | 0.95 (0.93 - 0.96) | *reference* | *reference* |
| Missing | 31 | 3 |  |  |  |
