# Supplementary material for: Educating the masses to address a global public health priority: The Preventing Dementia Massive Open Online Course (MOOC)
Source: PLoS One. 2022 May 4;17(5):e0267205. doi: 10.1371/journal.pone.0267205 (PMC9067672; doi:10.1371/journal.pone.0267205)
Supplement: S7 Table — (DOCX) [file pone.0267205.s008.docx]

**S7 Table: Associations between affirmation of the statement “The MOOC has had an impact on my behaviour and lifestyle choices” and participant demographics.**

|  | **Affirmed** | **Not affirmed** | **p-value** | **Age comparisons (years)** | **Odds ratio  (confidence interval)** |
| --- | --- | --- | --- | --- | --- |
| **Age** |  |  | 0.00003 | 25 vs 50 | 1.19 (1.12 - 1.27) |
| Mean (standard deviation) | 52.57 (13.65) | 53.17 (14.45) |  | 50 vs 70 | 0.79 (0.8 - 0.77) |
| Missing, n (%) | 638 (4.5) | 113 (27.09) |  | 70 vs 90 | 0.64 (0.83 - 0.5) |

|  | **Affirmed** | **Not affirmed** | **Proportion affirmed  (confidence interval)** | **p-value** | **Odds ratio (confidence interval)** |
| --- | --- | --- | --- | --- | --- |
| **Gender** |  |  |  |  |  |
| Male | 1755 | 378 | 0.82 (0.81 - 0.84) | 0.00000 | 0.74 (0.66 - 0.83) |
| Female | 12365 | 1966 | 0.86 (0.86 - 0.87) | *reference* | *reference* |
| Missing | 55 | 11 |  |  |  |
| **Occupation** |  |  |  |  |  |
| Health occupation | 8530 | 1296 | 0.87 (0.86 - 0.87) | 0.00011 | 1.20 (1.1 - 1.32) |
| Non-health occupation | 4620 | 845 | 0.85 (0.84 - 0.85) | *reference* | *reference* |
| Missing | 1025 | 214 |  |  |  |
| **Education** |  |  |  |  |  |
| Post-secondary education | 11394 | 1911 | 0.86 (0.85 - 0.86) | 0.0044 | 0.83 (0.72 - 0.94) |
| Lower level of education | 2108 | 292 | 0.88 (0.86 - 0.89) | *reference* | *reference* |
| Missing | 673 | 152 |  |  |  |
| **Country of residence** |  |  |  |  |  |
| High income | 13320 | 2273 | 0.85 (0.85 - 0.86) | 0.00000 | 0.55 (0.43 - 0.7) |
| Low or middle income | 825 | 78 | 0.91 (0.89 - 0.93) | *reference* | *reference* |
| Missing | 30 | 4 |  |  |  |
