# Supplementary material for: Educating the masses to address a global public health priority: The Preventing Dementia Massive Open Online Course (MOOC)
Source: PLoS One. 2022 May 4;17(5):e0267205. doi: 10.1371/journal.pone.0267205 (PMC9067672; doi:10.1371/journal.pone.0267205)
Supplement: S8 Table — (DOCX) [file pone.0267205.s009.docx]

**S8 Table: Associations between affirmation of the statement “I have already applied the knowledge I have gained from the MOOC” and participant demographics.**

|  | **Affirmed** | **Not affirmed** | **p-value** | **Age comparisons (years)** | **Odds ratio  (confidence interval)** |
| --- | --- | --- | --- | --- | --- |
| **Age** |  |  | 0.00001 | 25 vs 50 | 1.23 (1.17 - 1.29) |
| Mean (standard deviation) | 52.6 (13.55) | 52.77 (14.34) |  | 50 vs 70 | 0.83 (0.84 - 0.82) |
| Missing, n (%) | 559 (4.64) | 191 (12.53) |  | 70 vs 90 | 0.72 (0.9 - 0.58) |

|  | **Affirmed** | **Not affirmed** | **Proportion affirmed  (confidence interval)** | **p-value** | **Odds ratio (confidence interval)** |
| --- | --- | --- | --- | --- | --- |
| **Gender** |  |  |  |  |  |
| Male | 1511 | 624 | 0.71 (0.69 - 0.73) | 0.0139*# | 0.88 (0.8 - 0.98) |
| Female | 10497 | 3822 | 0.73 (0.73 - 0.74) | *reference* | *reference* |
| Missing | 52 | 14 |  |  |  |
| **Occupation** |  |  |  |  |  |
| Health occupation | 7374 | 2453 | 0.75 (0.74 - 0.76) | 0.00000 | 1.25 (1.16 - 1.34) |
| Non-health occupation | 3857 | 1602 | 0.71 (0.69 - 0.72) | *reference* | *reference* |
| Missing | 829 | 405 |  |  |  |
| **Education** |  |  |  |  |  |
| Post-secondary education | 9816 | 3485 | 0.74 (0.73 - 0.75) | 0.02617# | 1.12 (1.01 - 1.23) |
| Lower level of education | 1716 | 680 | 0.72 (0.7 - 0.73) | *reference* | *reference* |
| Missing | 528 | 295 |  |  |  |
| **Country of residence** |  |  |  |  |  |
| High income | 11317 | 4270 | 0.73 (0.72 - 0.73) | 0.00001 | 0.69 (0.58 - 0.81) |
| Low or middle income | 713 | 186 | 0.79 (0.77 - 0.82) | *reference* | *reference* |
| Missing | 30 | 4 |  |  |  |

*Indicates significant individual finding was not significant in combined model adjusted for other demographics.

#Indicates that finding was no longer significant after adjusting for multiple comparisons (6 models from one dataset).
