# Supplementary material for: Educating the masses to address a global public health priority: The Preventing Dementia Massive Open Online Course (MOOC)
Source: PLoS One. 2022 May 4;17(5):e0267205. doi: 10.1371/journal.pone.0267205 (PMC9067672; doi:10.1371/journal.pone.0267205)
Supplement: S9 Table — (DOCX) [file pone.0267205.s010.docx]

**S9 Table: Associations between affirmation of the statement “I would recommend the MOOC to others” and participant demographics.**

|  | **Affirmed** | **Not affirmed** | **p-value** | **Age comparisons (years)** | **Odds ratio  (confidence interval)** |
| --- | --- | --- | --- | --- | --- |
| **Age** |  |  | 0.00000 | 25 vs 50 | 2.87 (2.79 - 2.95) |
| Mean (standard deviation) | 52.71 (13.72) | 49.56 (16.9) |  | 50 vs 70 | 0.66 (0.68 - 0.63) |
| Missing, n (%) | 742 (4.55) | 7 (348.36) |  | 70 vs 90 | 0.77 (3.05 - 0.2) |

|  | **Affirmed** | **Not affirmed** | **Proportion affirmed  (confidence interval)** | **p-value** | **Odds ratio (confidence interval)** |
| --- | --- | --- | --- | --- | --- |
| **Gender** |  |  |  |  |  |
| Male | 2082 | 46 | 0.98 (0.97 - 0.98) | 0.00014* | 0.53 (0.38 - 0.74) |
| Female | 14163 | 165 | 0.99 (0.99 - 0.99) | *reference* | *reference* |
| Missing | 64 | 2 |  |  |  |
| **Occupation** |  |  |  |  |  |
| Health occupation | 9721 | 101 | 0.99 (0.99 - 0.99) | 0.18594 | 1.23 (0.9 - 1.67) |
| Non-health occupation | 5395 | 69 | 0.99 (0.98 - 0.99) | *reference* | *reference* |
| Missing | 1193 | 43 |  |  |  |
| **Education** |  |  |  |  |  |
| Post-secondary education | 13152 | 144 | 0.99 (0.99 - 0.99) | 0.47508 | 1.16 (0.76 - 1.69) |
| Lower level of education | 2372 | 30 | 0.99 (0.98 - 0.99) | *reference* | *reference* |
| Missing | 785 | 39 |  |  |  |
| **Country of residence** |  |  |  |  |  |
| High income | 15403 | 185 | 0.99 (0.99 - 0.99) | 0.00000 | 2.67 (1.75 - 3.93) |
| Low or middle income | 872 | 28 | 0.97 (0.96 - 0.98) | *reference* | *reference* |
| Missing | 34 | 0 |  |  |  |

*Indicates significant individual finding was not significant in combined model adjusted for other demographics.
